# The Weight of Genetic Drift: A Pedigree-Based Evaluation of the Breton Horse Population in Brazil

**DOI:** 10.1155/2024/4714077

**Published:** 2024-08-23

**Authors:** Bruno B. Medeiros, Kate M. C. Barcelos, Millena Oliveira Andrade, Michele Cristina da Paz Carvalho, Victoria Rocha Miranda, Kalebe de Oliveira Maia, Susana Reinhardt, Laura Patterson Rosa

**Affiliations:** ^1^ Department of Agriculture and Industry Sul Ross State University, Alpine, TX, USA; ^2^ Escola de Veterinária e Zootecnia Universidade Federal de Goias, Campus Samambaia, Goiânia, Brazil; ^3^ Superintendência do Serviço de Registro Genealógico da Raça Bretã Associação Brasileira de Criadores do Cavalo Bretāo, Sao Paulo, Brazil; ^4^ Department of Veterinary Clinical Sciences College of Veterinary Medicine Long Island University, New York, NY, USA

## Abstract

The genetic diversity of Breton horses in Brazil is a critical concern, mainly due to the small population size and low number of births per year. Given that the inbreeding was overlooked by breeders for multiple generations, we estimated the genetic diversity of this population utilizing pedigree-based measures of population diversity. A total of 1394 six-generation pedigrees representing the full population of registered Breton horses in Brazil defined a total population (TP, *N* = 2679), with horses born between 2000 and 2022, reproductively active and alive, as reported by the breed association, representing the reference population (RP, *N* = 731). Using the R package *PurgeR*, we estimated inbreeding coefficient (*F*_*ped*_), maternal inbreeding coefficient (*F*_*da* *m*_), paternal inbreeding coefficient (*F*_*sire*_), individual reproductive values, number of equivalents to complete generations (*t*), and unbiased ancestral inbreeding coefficient (*Fa*). We established the equivalent complete generations (ECG), effective population size (*Ne*), total number of founders (*Nf*), effective number of founders (*Nfe*), total number of ancestors (*Na*), effective number of ancestors (*Nae*), founder genomes (*Ng*), and the inbreeding coefficient estimated with effective population size (*Ne*) and generation numbers (*t*) (*F*_*Ne*:*t*_), as well as *Nfe*/*Nae* and *Nfe*/*Ng* ratios for the RP. The RP inbreeding levels have stabilized, although they are still significantly rising by generation (*t*), and the *Nfe*/*Ng* ratio strongly suggests genetic drift. Pedigree-based analysis demonstrates that only five stallions have sired 52.83% of the RP individuals, which along with the *Nae* value of 36.73 implies that the observed inbreeding can be arising from patrilines. Our results suggest that observed inbreeding is due to Popular Sire Effect, highlighting the importance of monitoring breeding schemes and genetic diversity to maintain health.

## 1. Introduction

Originally from the Brittany and Loire Atlantique regions of France, the Brazilian population of Breton draft horses (*Equus caballus*) is based on fewer than 180 individuals. The base population results from importations of animals and genetic material spanning from 1925 to early 2010's [1]. Due to the small number of founder individuals, a small population size of 1420 horses, and fewer than sixty-five births per year from 2004 to 2011 (personal communication, Brazilian Association of Breton Horse Breeders-BABHB), the current status of the Breton breed in Brazil is concerning. The deleterious potential of inbreeding and loss of genetic diversity presents a challenge to the health and overall breed maintenance in Brazil [2, 3].

A previous inbreeding estimation of the Breton horse in Brazil evaluating the maternal lineages through mitochondrial DNA (*mtDNA*) resulted in average genomic-based (*F*_*st*_) inbreeding estimates of 0.17, yet this approach presents limitations given the solely maternal perspective of mtDNA, especially if the inbreeding is mainly of paternal origin [4]. Pedigree analysis can be utilized as a low-cost, accessible tool when compared to genomic-based studies [5] to estimate measures of genetic diversity, inbreeding, differences in estimated and effective population, as well as signs of founder effect [2, 5]. Pedigree analysis successfully predicted inbreeding and its associations with health and fitness parameters such as fertility and growth in dogs, laboratory mice, sheep, and other animals [2, 3, 5, 6]. Pedigree-based evaluation was also effectively used in the Brazilian Sport Horse (BH) to measure genetic diversity [7, 8]. Furthermore, pedigree analysis can serve as baseline for future genomic-based assessments, providing approximations of parameters supporting evaluations of genetic diversity [3].

To assess inbreeding and other population diversity measurements, we performed a pedigree-based analysis of 1,394 individuals registered with BABHB, representing the complete Breton horse population in Brazil, through 6-generation pedigrees. We evaluated the resulting inbreeding coefficient (*F*_*ped*_) in the total and reproductively active or reference population, the estimated and effective number of founders and ancestors, and signs of founder effect.

## 2. Materials and Methods

### 2.1. Ethics Statement

Institutional Care and Use Committee (IACUC) ethical approval was not required as this research utilized nonidentifiable computational pedigree data collected voluntarily and provided by the Brazilian Association of Breton Horse Breeders.

### 2.2. Animals

One thousand, three hundred and ninety-four individuals and their respective pedigrees representing the registered population of Breton horses registered in Brazil and containing up to the sixth generation of ancestors were provided by BABHB and utilized in this study. This data was manually entered in a Microsoft Excel spreadsheet following the recommended guidelines by the *purgeR* package [9], resulting in 2679 total entries representing each individual and respective ancestors noted in the pedigrees.

We further divided the population in total (TP) and reference (RP) population, with the reference constituted of reproductively active, alive individuals as reported by the breed association, born from the year 2000 to 2022 (*N* = 731 horses), and the total population representing all individuals observed in the pedigrees provided by BABHB including horses ancestral to the reference population (*N* = 2679, or 1948 horses added to the RP). The RP represents the currently active Breton population.

### 2.3. Pedigree Analysis

The R package *purgeR* [9] was applied to the dataset using RStudio [10] to obtain measurements of inbreeding and population diversity as inbreeding coefficient (*F*_*ped*_), maternal inbreeding coefficient (*F*_*da* *m*_), paternal inbreeding coefficient (*F*_*sire*_), individual reproductive values, number of equivalents to complete generations (t), and unbiased ancestral inbreeding coefficient (*Fa*) (Sup.  Table 1). Using these results, we established for the RP: equivalent complete generations (*ECG*), effective population size (*Ne*), total number of founders (*Nf*), effective number of founders (*Nfe*), total number of ancestors (*Na*), effective number of ancestors (*Nae*), founder genomes (*Ng*), and the inbreeding coefficient estimated with effective population size (*Ne*) and generation numbers (*t*) (referred to here as *F*_*Net*_), following published guidelines [9]. We further compared *Nfe*/*Nae* and *Nfe*/*Ng* ratios to evaluate founder effects in this population [5]. Statistical analysis was performed using JMP Pro 16 (SAS Institute, Inc., Cary, NC). Year born and *F*_*ped*_ for both TP and RP were evaluated for normality using the Anderson–Darling test. Standard Least Squares was used to assess linear association of *F*_*ped*_ to year of birth and generation (*t*) for the TP and RP. Results were considered statistically significant at *p* ≤ 0.05.

## 3. Results and Discussion

**Figure 1:**
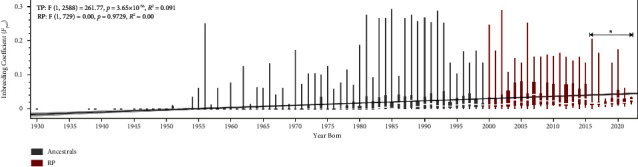
Inbreeding coefficient (*F*_*ped*_) increases significantly by year born (*p*=3.65 × 10^−56^,  *R*^2^=0.091) in the total population (TP) yet has stabilized in the reference population (RP). The asterisk marked section demonstrates the small decrease in average *F*_*ped*_ for individuals born from 2016 to 2022 (*N* = 127, mean = 0.030, SD ± 0.034).

While average *F*_*ped*_ inbreeding coefficients significantly increase by birth year in the TP (*F*(1,2588) = 261.77, *p* = 3.65 × 10^–56^), there is no association observed in the RP, where *F*_*pe*_*d* seems to have stabilized (*F*(1,729) = 0.001, *p*=0.97) (Figure 1). Still, in the RP, the *F*_*pe*_*d* significantly increases by generation (*t*) (*F*(1,729) = 65.78, *p* = 2.13 × 10^–15^), suggesting that inbreeding levels may be rising with each generation. The *F*_*ped*_ superficial stabilization in recent years is possibly due to a lower overall number of reproductively active animals or reflecting the increasing use of imported semen and embryos from 2006 to 2015, slowing down the rise in inbreeding [1]. Supporting the introduction of new genetics through imported germline, we observe a small decrease in the average *F*_*ped*_ of individuals born from 2016 to 2022 (*N* = 127, mean = 0.030, standard deviation/SD ± 0.034). A similar effect was observed in the BH population, where recent introduction of imported individuals in the breeding program has decreased the overall *F*_*ped*_ [8]. Furthermore, despite the RP effective population size (*Ne*) of 83.86 animals (SD ± 3.56) (Table 1) being lower than values observed in other Brazilian breeds [8, 11], the genetic diversity of the Breton horse in Brazil has not reached levels considered critical (*Ne* ≤ 50) [12, 13]. Thus, the observed RP *F*_*ped*_ stabilization is likely a product of artificial insemination and embryo transfer technologies utilized in the breeding program and the relatively low number of reproductive active animals. Still, preventative measures focusing on increasing genetic diversity are necessary to avoid reaching critical effective levels for this population, especially since there are no set limits for the number of annual offspring of a stallion [1].

The total number of ancestors (*Na*) is measured as 1697 horses, while the number of founders (*Nf*) was 366 individuals (Table 1). *Na* and *Nf* are directly dependent on the number of individuals evaluated and on the number of pedigree generations used in the analysis [11]. Studies of other horse populations as the BH (*Na* = 1736, RP = 8353) [8] and Mangalarga Marchador (*Na* = 1572, RP = 172,508) [11] utilizing complete pedigrees demonstrated similar *Na* values despite the higher number of RP individuals, suggesting that the Breton 6-generation pedigrees encompass the diversity of this population. Additionally, the *Nfe* = 107.03 (29.24% of *Nf*), the *Nae* = 36.73 (2.16% of *Na*), and *Ng* = 17.66 (SD ± 2.89) (Table 1) values support the historical accounts of the breed early history in Brazil [1] and suggest an overuse of few individuals.

While the *Nfe*/*Nae* ratio of 2.91 implies a bottleneck effect, the *Nfe*/*Ng* ratio of 6.06 strongly suggests genetic drift in this population [14–17]. Both observed ratios for the Breton population also indicate a stringent founder effect [5]. Comparatively, the Breton has higher *Nfe*/*Nae* ratios than other horse populations as the Crioulo (1.21) [18], BH (1.72) [8], Turkish Arab (1.81) [15], Pura Raza Espanola (1.78) [17], Lusitano (2.34) [19], Maremmano (2.47) [16]; yet lower than the Hanoverian (3.15) [20] and American Shire (3.7) [21]. These studies also noted strong signals of genetic drift in their populations, although *Nfe*/*Ng* reported ratios were lower than the Breton RP (6.06), as the BH (2.74) [8], Turkish Arab (4,16) [15], Lusitano (4.57) [19], and Maremmano (5.39) [16]. While a modest bottleneck effect is commonly observed in horse populations under human selection [22], our results suggest that genetic drift rather than a bottleneck effect is the most likely cause of loss of genetic diversity in the Breton horse population in Brazil.

Although *mtDNA* analysis of Brazilian Breton horses does not suggest a high *F*_*st*_ (17.68%) for the population when compared to other Brazilian equid populations, *mtDNA* is limited to maternal lines [4]. Our RP results demonstrated an average maternal inbreeding coefficient (*F*_*da* *m*_) ranging from 0% to 26.51% (mean = 2.71%, SD ± 3.60%) while the paternal inbreeding coefficients (*F*_*sire*_) range from 0% to 13.49% (mean = 1.80%, SD ± 1.87%), suggesting that higher inbreeding values arise from the maternal lines (Table 1). Still, pedigree analysis showed that of 729 stallions represented in the RP, just five (0.68%) have sired over half or 52.83% of the RP individuals. The most prolific sire was born in 1982 and had 54 offspring, a substantial number when compared to the most prolific dam which had only 12 offspring in her lifetime. Furthermore, individual #1205 of the RP demonstrated a reproductive value of 0.0020, the highest in our analysis, and sired 44 offspring in the RP population (Sup. Table 1). Even with modern equine reproductive technologies, males have a higher likelihood of genetic propagation than females, given the feasibility of germplasm collection, storage, and utilization [23]. In addition, the resulting *Nae* value of 36.73 also indicates an overuse of some individuals [14]. Likewise, the loss of genetic diversity noted in the Turkish Arab horse and BH is implied as caused by the restricted number of stallions used for breeding [8, 15]. Although the pedigree-based *F*_*da* *m*_ is higher than the *F*_*sire*_, increasing inbreeding levels in the Brazilian population of Breton horses can be patriline associated, which may have stemmed from the overuse of specific sires during the history of the breed, an effect commonly known as “Popular Sire Effect” [24], and the selection of their offspring. The Popular Sire Effect and its consequences are demonstrated by the Y chromosome loss of diversity through the domestication selection bias for stallions during modern human breeding practices [22]. Further evaluations of the Y chromosome diversity in this population of horses can provide support for improved breeding practices and patriline diversification.

The RP pedigree analysis demonstrated inbreeding coefficients (*F*_*ped*_) between 0.0 and 0.29 (mean = 0.036, SD ± 0.0399) and accounting for effective population size (*Ne*) and generation number (*t*), a mean inbreeding coefficient (*F*_*Ne*:*t*_) of 5.31% (Table 1). While the RP mean is higher than observations in Brazilian autochthonous breeds such as the BH (0.033) [8], Mangalarga Marchador (0.011) [11], and Crioulo (0.0088) [18], other foreign breeds have noted higher *F*_*ped*_ such as the Turkish Arab horse (0.046) [15], Mallorquí (0.047) [25], yet not higher than the *F*_*Ne*:*t*_ of 5.31%, except for observations in the Lusitano horse (0.1134) [19]. The variance between the noted *F*_*ped*_ values can be due to reproductive population diversity, breeding practices or pedigree completeness. A limitation of the pedigree analysis is the lack of information on complete generations (e.g., missing or unknown ancestors), as evidenced by the observed *F*_*ped*_ values of 0.00. While the RP population has a mean Equivalent Complete Generations (ECG) of 7.05 (SD ± 0.89), representing the pedigree completeness of this group [14], 1002 ancestral individuals (51.44%) have a *F*_*ped*_ of 0.00, likely due to the lack of pedigree ancestor information. Furthermore, comparisons between genomic and pedigree-based inbreeding in Arabian [26] and Mangalarga [27] horses found moderate to low correlation between genomic (*F*_*st*_) and pedigree-based (*F*_*ped*_) inbreeding coefficients. Therefore, the RP *F*_*ped*_ mean of 3.6% (SD ± 3.99%) may be underestimated in our population, and the *F*_*Ne*:*t*_ may represent the mean inbreeding in this population more accurately. The observed *F*_*ped*_ of 0% can be an artifact of limiting the pedigree entries to Brazilian-registered horses only or due to the lack of records for the early Breton populations. Thus, pedigree-based inbreeding coefficients may be a good baseline tool for analyzing inbreeding yet presents limitations in precisely measuring the genomic diversity at both individual and population levels.

Due to the suggested genetic drift that occurred in the population of Breton horses in Brazil, their management could benefit from increasing diversity through the introduction of new, genetically diverse individuals, especially regarding male lines. Since pedigree analysis is sensitive to the incomplete written records observed in real populations [14], and given the discrepancy between the *F*_*ped*_ results and previously reported *F*_*mtDNA*_ [4] for the Breton breed, further evaluations using nuclear genomic approaches are recommended to obtain more precise population inbreeding levels.

## 4. Conclusion

Our investigation highlighted the significant effects of genetic drift over many generations. Using pedigree data from the complete population of registered individuals, we noted that the RP inbreeding coefficient has apparently stabilized, although the inbreeding is rising by generation. This can be due to modern management practices not limiting progeny by individual males and the smaller influence of imported germplasm, an essential aspect in maintaining the genetic diversity of this geographically isolated population. We reiterate the importance of managing horse populations using genetic-based methodologies to avoid inbreeding depression and the loss of genetic diversity. Furthermore, the breed association can utilize current pedigree records to establish mating strategies with the goal of increasing the population genetic diversity. Results from pedigree and further genomic analysis can support improved genotype-based selection to maintain genetic diversity and expand the understanding of genetic drift impacts in domestic, small-population equine breeds such as the Breton horse in Brazil. Further studies assessing the genomic inbreeding and diversity of Y chromosome are recommended.

## Figures and Tables

**Table 1:** Inbreeding and populational parameters of the Breton horse reference population (RP) resulting from *purgeR* pedigree analysis.

Reference population (*N* = 731)	Result	Mean (95% CI)	SD ±
Inbreeding coefficient (*F*_*ped*_)	0%–28.99%	3.6% [3.32–3.90]	3.99%
Unbiased ancestral inbreeding coefficient (*Fa*)	0%–26.74%	8.06% [7.78–8.34]	3.90%
Maternal inbreeding coefficient (*F*_*da* *m*_)	0%–26.51%	2.71% [2.45–2.97]	3.60%
Paternal inbreeding coefficient (*F*_*sire*_)	0%–13.49%	1.80% [1.67–1.94]	1.87%
Equivalent complete generations (ECG)	7.05	—	0.89
Effective population size (*Ne*)	83.86	—	3.56
Inbreeding coefficient estimated with *Ne* and *t* (*F*_*Ne*:*t*_)	5.31%	—	—
Total number of founders (*Nf*)	366	—	—
Effective number of founders (*Nfe*)	107.03	—	—
Total number of ancestors (*Na*)	1697	—	—
Effective number of ancestors (*Nae*)	36.73	—	—
Founder genomes (*Ng*)	17.66	—	Standard error = 2.9
*Nfe*/*Nae* ratio	2.91	—	—
*Nfe*/*Ng* ratio	6.06	—	—

(—) There is no standard deviation (SD) and/or mean and CI for this data.

## Data Availability

The data supporting the findings of this study are available in the Supplementary Material of this article.

## Supplementary Materials

Supplementary Materials Supplemental Table 1. PurgeR data generated for 1394 individual BABHB pedigrees or 2679 individual entries and respective inbreeding coefficients (Fped, Fa, Fdam, and Fsire), reproductive values, and t results. Reference population is coded as a TRUE/FALSE character.
