# Supplementary material for: The Weight of Genetic Drift: A Pedigree-Based Evaluation of the Breton Horse Population in Brazil
Source: Vet Med Int. 2024 Aug 23;2024:4714077. doi: 10.1155/2024/4714077 (PMC11364472; doi:10.1155/2024/4714077)
Supplement: Supplementary Materials — Supplemental Table 1. PurgeR data generated for 1394 individual BABHB pedigrees or 2679 individual entries and respective inbreeding coefficients (Fped, Fa, Fdam, and Fsire), reproductive values, and t results. Reference population is coded as a TRUE/FALSE character. [file 4714077.f1.pdf]

Supplemental Table 1. PurgeR data generated for 1394 individual BABHB pedigrees or 2679 individual entries and respective Inbreeding Coefficients (Fped, Fa, Fdam and Fsire), reproductive values and t Results. Reference Population is coded as a TRUE/FALSE character.

Individual ID	Year of Birth	Sex	Sire ID	Dam ID	Reference Population	Reproductive Value	*t*	*Fped*	*Fa*	*Fdam*	*Fsire*
1	1950	Female	0	0	FALSE	0	0	0	0		
2	1960	Male	0	0	FALSE	0	0	0	0		
3	1965	Female	0	0	FALSE	0	0	0	0		
4	1930	Male	0	0	FALSE	0	0	0	0		
5	1957	Female	0	0	FALSE	0	0	0	0		
6	1950	Male	0	0	FALSE	0	0	0	0		
7	1965	Male	0	0	FALSE	0	0	0	0		
8	1966	Female	7	5	FALSE	0	1	0	0	0	0
9	1967	Female	0	0	FALSE	0	0	0	0		
10	1953	Male	0	0	FALSE	0	0	0	0		
11	1970	Male	0	0	FALSE	0	0	0	0		
12	1965	Female	6	1	FALSE	0	1	0	0	0	0
13	1945	Male	4	0	FALSE	0	0.5	0	0		0
14	1960	Male	2	3	FALSE	0	1	0	0	0	0
15	1977	Female	0	0	FALSE	0	0	0	0		
16	1960	Male	0	0	FALSE	0	0	0	0		
17	1965	Female	0	0	FALSE	0	0	0	0		
18	1947	Female	0	0	FALSE	0	0	0	0		
19	1965	Female	14	12	FALSE	0	2	0	0	0	0
20	1953	Male	13	8	FALSE	0	1.75	0	0	0	0
21	1955	Female	10	0	FALSE	0	0.5	0	0		0
22	1965	Male	16	15	FALSE	0	1	0	0	0	0
23	1965	Female	0	0	FALSE	0	0	0	0		
24	1965	Female	11	9	FALSE	0	1	0	0	0	0
25	1953	Male	0	0	FALSE	0	0	0	0		
26	1960	Male	0	0	FALSE	0	0	0	0		
27	1953	Male	0	0	FALSE	0	0	0	0		
28	1965	Female	0	0	FALSE	0	0	0	0		
29	1958	Male	0	0	FALSE	0	0	0	0		
30	1960	Male	0	0	FALSE	0	0	0	0		
31	1960	Female	0	0	FALSE	0	0	0	0		
32	1958	Female	0	0	FALSE	0	0	0	0		
33	1957	Male	0	0	FALSE	0	0	0	0		
34	1960	Female	0	0	FALSE	0	0	0	0		
35	1968	Female	0	0	FALSE	0	0	0	0		
36	1958	Female	0	0	FALSE	0	0	0	0		
37	1965	Male	13	17	FALSE	0	1.25	0	0	0	0
38	1960	Male	33	32	FALSE	0	1	0	0	0	0
39	1970	Male	0	0	FALSE	0	0	0	0		
40	1970	Female	26	28	FALSE	0	1	0	0	0	0
41	1960	Female	0	0	FALSE	0	0	0	0		
42	1949	Male	0	0	FALSE	0	0	0	0		
43	1965	Female	0	0	FALSE	0	0	0	0		
44	1951	Male	0	0	FALSE	0	0	0	0		
45	1952	Male	0	0	FALSE	0	0	0	0		
46	1965	Female	30	19	FALSE	0	2	0	0	0	0
47	1965	Female	0	0	FALSE	0	0	0	0		
48	1960	Male	25	23	FALSE	0	1	0	0	0	0
49	1970	Female	22	24	FALSE	0	2	0	0	0	0
50	1971	Male	27	18	FALSE	0	1	0	0	0	0
51	1960	Female	29	31	FALSE	0	1	0	0	0	0
52	1960	Male	20	21	FALSE	0	2.125	0	0	0	0

53	1956	Female	0	0	FALSE	0	0	0	0		
54	1965	Female	14	12	FALSE	0	2	0	0	0	0
55	1965	Female	30	36	FALSE	0	1	0	0	0	0
56	1958	Male	26	34	FALSE	0	1	0	0	0	0
57	1956	Female	0	0	FALSE	0	0	0	0		
58	1960	Female	20	35	FALSE	0	1.875	0	0	0	0
59	1975	Male	50	51	FALSE	0	2	0	0	0	0
60	1966	Male	0	0	FALSE	0	0	0	0		
61	1967	Female	0	0	FALSE	0	0	0	0		
62	1960	Female	37	41	FALSE	0	1.625	0	0	0	0
63	1975	Female	52	49	FALSE	0	3.0625	0	0	0	0
64	1958	Female	0	0	FALSE	0	0	0	0		
65	1949	Female	0	0	FALSE	0	0	0	0		
66	1957	Male	0	0	FALSE	0	0	0	0		
67	1965	Female	0	0	FALSE	0	0	0	0		
68	1956	Female	44	0	FALSE	0	0.5	0	0		0
69	1957	Male	42	43	FALSE	0	1	0	0	0	0
70	1959	Male	0	0	FALSE	0	0	0	0		
71	1952	Male	38	40	FALSE	0	2	0	0	0	0
72	1960	Male	39	0	FALSE	0	0.5	0	0		0
73	1954	Female	48	46	FALSE	0	2.5	0	0	0	0
74	1960	Male	45	47	FALSE	0	1	0	0	0	0
75	1968	Male	13	53	FALSE	0	1.25	0	0	0	0
76	1966	Male	52	57	FALSE	0	2.0625	0	0	0	0
77	1968	Female	70	63	FALSE	0	2.53125	0	0	0	0
78	1951	Male	69	55	FALSE	0	2	0	0	0	0
79	1950	Male	66	65	FALSE	0	1	0	0	0	0
80	1974	Male	20	68	FALSE	0	2.125	0	0	0	0
81	1962	Female	60	61	FALSE	0	1	0	0	0	0
82	1960	Female	56	64	FALSE	0	1.5	0	0	0	0
83	1960	Male	59	62	FALSE	0	2.8125	0	0	0	0
84	1960	Male	74	19	FALSE	0	2.5	0	0	0	0
85	1955	Male	74	54	FALSE	0	2.5	0	0	0	0
86	1965	Female	56	67	FALSE	0	1.5	0	0	0	0
87	1959	Female	71	73	FALSE	0	3.25	0	0	0	0
88	1965	Female	72	58	FALSE	0	2.1875	0	0	0	0
89	1975	Female	76	0	FALSE	0	1.53125	0	0		0
90	1968	Female	79	81	FALSE	0	2	0	0	0	0
91	1968	Female	78	87	FALSE	0	3.625	0.015625	0	0	0
92	1970	Male	83	82	FALSE	0	3.15625	0	0	0	0
93	1968	Female	85	86	FALSE	0	3	0	0	0	0
94	1975	Male	80	88	FALSE	0	3.15625	0.0625	0	0	0
95	1980	Male	75	77	FALSE	0	2.890625	0.015625	0	0	0
96	1958	Male	84	73	FALSE	0	3.5	0.0625	0	0	0
97	1938	Male	0	0	FALSE	0	0	0	0		
98	1973	Female	95	90	FALSE	0	3.4453125	0	0.0078125	0	0.015625
99	1960	Male	96	91	FALSE	0	4.5625	0.078125	0.0390625	0.015625	0.0625
100	1980	Male	94	89	FALSE	0	3.34375	0.0234375	0.03125	0	0.0625
101	1976	Female	92	93	FALSE	0	4.078125	0.03125	0	0	0
102	1948	Male	97	34	FALSE	0	1	0	0	0	0
103	1983	Female	99	101	FALSE	0	5.3203125	0.0166016	0.0726929	0.03125	0.078125
104	1985	Male	100	98	FALSE	0	4.3945313	0.0146484	0.0308838	0	0.0234375
105	1955	Female	0	0	FALSE	0	0	0	0		
106	1965	Female	0	0	FALSE	0	0	0	0		
107	1965	Female	102	82	FALSE	0	2.25	0.0625	0	0	0
108	1993	Male	104	103	FALSE	0	5.8574219	0.0010376	0.0665837	0.0166016	0.0146484

109	1959	Female	102	105	FALSE	0	1.5	0	0	0	0
110	1970	Female	102	106	FALSE	0	1.5	0	0	0	0
111	1950	Female	0	0	FALSE	0	0	0	0		
112	1950	Male	0	0	FALSE	0	0	0	0		
113	1970	Female	13	8	FALSE	0	1.75	0	0	0	0
114	1965	Female	0	0	FALSE	0	0	0	0		
115	1965	Female	0	0	FALSE	0	0	0	0		
116	1965	Female	108	107	FALSE	0	5.0537109	0.0239258	0.0650261	0.0625	0.0010376
117	1968	Male	56	109	FALSE	0	2.25	0.0625	0	0	0
118	1972	Female	85	110	FALSE	0	3	0	0	0	0
119	1958	Female	20	111	FALSE	0	1.875	0	0	0	0
120	1959	Male	112	113	FALSE	0	1.875	0	0	0	0
121	1965	Female	97	114	FALSE	0	1	0	0	0	0
122	1973	Female	56	115	FALSE	0	1.5	0	0	0	0
123	1977	Female	0	0	FALSE	0	0	0	0		
124	1978	Female	117	116	FALSE	0	4.6518555	0.0913086	0.074948	0.0239258	0.0625
125	1974	Male	92	118	FALSE	0	4.078125	0.0078125	0	0	0
126	1975	Male	0	0	FALSE	0	0	0	0		
127	1965	Male	120	119	FALSE	0	2.875	0.0625	0	0	0
128	1965	Female	56	121	FALSE	0	2	0	0	0	0
129	1967	Female	71	122	FALSE	0	2.75	0.03125	0	0	0
130	1980	Female	126	123	FALSE	0	1	0	0	0	0
131	1970	Male	125	124	FALSE	0	5.3649902	0.0673981	0.0836129	0.0913086	0.0078125
132	1968	Male	71	128	FALSE	0	3	0.03125	0	0	0
133	1967	Male	131	129	FALSE	0	5.0574951	0.0422974	0.0883128	0.03125	0.0673981
134	1982	Female	127	130	FALSE	0	2.9375	0	0.03125	0	0.0625
135	1970	Female	132	129	FALSE	0	3.875	0.171875	0.03125	0.03125	0.03125
136	1945	Male	0	0	FALSE	0	0	0	0		
137	1947	Female	0	0	FALSE	0	0	0	0		
138	1980	Female	0	0	FALSE	0	0	0	0		
139	1980	Male	133	134	FALSE	0	4.9974976	0.0010529	0.0790624	0	0.0422974
140	1985	Female	100	135	FALSE	0	4.609375	0	0.1258545	0.171875	0.0234375
141	1970	Male	0	0	FALSE	0	0	0	0		
142	1950	Male	136	137	FALSE	0	1	0	0	0	0
143	1950	Female	0	0	FALSE	0	0	0	0		
144	1965	Female	139	138	FALSE	0	3.4987488	0	0.040016	0	0.0010529
145	1967	Female	0	0	FALSE	0	0	0	0		
146	1970	Female	0	144	FALSE	0	2.2493744	0	0.020008	0	
147	1950	Female	142	143	FALSE	0	1.5	0	0	0	0
148	1975	Male	141	140	FALSE	0	3.3046875	0	0.0629272	0	0
149	1964	Male	0	0	FALSE	0	0	0	0		
150	1960	Female	0	0	FALSE	0	0	0	0		
151	1967	Female	0	0	FALSE	0	0	0	0		
152	1968	Female	127	145	FALSE	0	2.4375	0	0.03125	0	0.0625
153	1972	Male	127	144	FALSE	0	4.1868744	0.0669327	0.051258	0	0.0625
154	1979	Female	149	146	FALSE	0	2.1246872	0	0.010004	0	0
155	1955	Female	148	147	FALSE	0	3.4023438	0	0.0314636	0	0
156	1960	Male	0	0	FALSE	0	0	0	0		
157	1956	Male	20	150	FALSE	0	1.875	0	0	0	0
158	1960	Female	39	151	FALSE	0	1	0	0	0	0
159	1966	Female	0	0	FALSE	0	0	0	0		
160	1979	Female	153	154	FALSE	0	4.1557808	0.0708666	0.0623819	0	0.0669327
161	1963	Male	20	155	FALSE	0	3.5761719	0.015625	0.0157318	0	0
162	1962	Female	156	152	FALSE	0	2.21875	0	0.015625	0	0
163	1965	Female	0	0	FALSE	0	0	0	0		
164	1975	Male	157	0	FALSE	0	1.4375	0	0		0

165	1970	Female	156	158	FALSE	0	1.5	0	0	0	0
166	1980	Female	0	160	FALSE	0	2.5778904	0	0.0644139	0.0708666	
167	1961	Female	0	162	FALSE	0	1.609375	0	0.0078125	0	
168	1968	Female	0	0	FALSE	0	0	0	0		
169	1964	Female	161	159	FALSE	0	2.7880859	0	0.0155555	0	0.015625
170	1967	Male	0	0	FALSE	0	0	0	0		
171	1965	Male	0	0	FALSE	0	0	0	0		
172	1970	Male	20	163	FALSE	0	1.875	0	0	0	0
173	1980	Female	164	165	FALSE	0	2.46875	0	0	0	0
174	1970	Female	171	168	FALSE	0	1	0	0	0	0
175	1965	Male	20	167	FALSE	0	2.6796875	0.0234375	0.0039063	0	0
176	1980	Male	133	166	FALSE	0	4.8176928	0.0247414	0.0956443	0	0.0422974
177	1968	Female	170	169	FALSE	0	2.394043	0	0.0077778	0	0
178	1967	Female	0	0	FALSE	0	0	0	0		
179	1970	Male	175	177	FALSE	0	3.5368652	0.0337372	0.017515	0	0.0234375
180	1975	Female	172	174	FALSE	0	2.4375	0	0	0	0
181	1972	Male	0	0	FALSE	0	0	0	0		
182	1985	Female	176	173	FALSE	0	4.6432214	0.0020829	0.0590097	0	0.0247414
183	1965	Male	0	0	FALSE	0	0	0	0		
184	1950	Female	183	178	FALSE	0	1	0	0	0	0
185	1979	Female	179	180	FALSE	0	3.9871826	0.0407715	0.0253306	0	0.0337372
186	1980	Male	181	182	FALSE	0	3.3216107	0	0.0304849	0.0020829	0
187	1970	Female	186	185	FALSE	0	4.6543967	0.006901	0.0477771	0.0407715	0
188	1960	Male	0	184	FALSE	0	1	0	0	0	
189	1965	Female	188	187	FALSE	0	3.8271983	0	0.0271742	0.006901	0
190	1968	Female	0	0	FALSE	0	0	0	0		
191	1954	Male	0	189	FALSE	0	2.4135992	0	0.0135871	0	
192	1943	Female	0	0	FALSE	0	0	0	0		
193	1976	Male	127	190	FALSE	0	2.4375	0	0.03125	0	0.0625
194	1974	Female	0	0	FALSE	0	0	0	0		
195	1975	Male	0	0	FALSE	0	0	0	0		
196	1958	Female	191	192	FALSE	0	2.2067996	0	0.0067935	0	0
197	1954	Male	13	0	FALSE	0	0.75	0	0		0
198	1968	Male	60	0	FALSE	0	0.5	0	0		0
199	1969	Female	0	0	FALSE	0	0	0	0		
200	1968	Female	0	0	FALSE	0	0	0	0		
201	1995	Male	195	194	FALSE	0	1	0	0	0	0
202	1962	Female	193	196	FALSE	0	3.3221498	0.0024128	0.0190218	0	0
203	1968	Male	0	0	FALSE	0	0	0	0		
204	1958	Female	197	0	FALSE	0	0.875	0	0		0
205	1985	Female	0	0	FALSE	0	0	0	0		
206	1968	Female	0	0	FALSE	0	0	0	0		
207	1971	Male	203	200	FALSE	0	1	0	0	0	0
208	1955	Male	198	199	FALSE	0	1.25	0	0	0	0
209	1968	Male	0	0	FALSE	0	0	0	0		
210	1980	Female	0	0	FALSE	0	0	0	0		
211	1968	Female	0	0	FALSE	0	0	0	0		
212	1983	Male	0	0	FALSE	0	0	0	0		
213	1968	Male	201	202	FALSE	0	3.1610749	0	0.0106944	0.0024128	0
214	1985	Female	0	0	FALSE	0	0	0	0		
215	1972	Male	127	205	FALSE	0	2.4375	0	0.03125	0	0.0625
216	1967	Female	213	211	FALSE	0	2.5805374	0	0.0053472	0	0
217	1989	Female	212	214	FALSE	0	1	0	0	0	0
218	1971	Female	0	0	FALSE	0	0	0	0		
219	1962	Male	208	204	FALSE	0	2.0625	0	0	0	0
220	1971	Male	209	206	FALSE	0	1	0	0	0	0

221	1967	Female	207	210	FALSE	0	1.5	0	0	0	0
222	1960	Female	0	0	FALSE	0	0	0	0		
223	1965	Female	0	0	FALSE	0	0	0	0		
224	1965	Female	156	158	FALSE	0	1.5	0	0	0	0
225	1970	Female	0	0	FALSE	0	0	0	0		
226	1970	Male	0	0	FALSE	0	0	0	0		
227	1990	Male	120	221	FALSE	0	2.6875	0	0	0	0
228	1952	Male	219	216	FALSE	0	3.3215187	0.0010582	0.0026736	0	0
229	1958	Male	0	0	FALSE	0	0	0	0		
230	1976	Female	220	218	FALSE	0	1.5	0	0	0	0
231	1993	Female	215	217	FALSE	0	2.71875	0	0.015625	0	0
232	1974	Female	0	0	FALSE	0	0	0	0		
233	1956	Female	0	0	FALSE	0	0	0	0		
234	1962	Female	157	204	FALSE	0	2.375	0.03125	0	0	0
235	1965	Female	85	223	FALSE	0	2.25	0	0	0	0
236	1960	Female	157	224	FALSE	0	2.6875	0	0	0	0
237	1970	Female	175	155	FALSE	0	4.0410156	0.0081787	0.0293579	0	0.0234375
238	1968	Male	0	0	FALSE	0	0	0	0		
239	1946	Female	229	222	FALSE	0	1	0	0	0	0
240	1965	Male	0	0	FALSE	0	0	0	0		
241	1957	Female	0	0	FALSE	0	0	0	0		
242	1960	Male	0	0	FALSE	0	0	0	0		
243	1976	Female	226	225	FALSE	0	1	0	0	0	0
244	1957	Male	0	0	FALSE	0	0	0	0		
245	1976	Male	228	230	FALSE	0	3.4107594	0	0.0018645	0	0.0010582
246	1982	Female	227	231	FALSE	0	3.703125	0.0351563	0.0078125	0	0
247	1969	Male	20	232	FALSE	0	1.875	0	0	0	0
248	1964	Male	52	233	FALSE	0	2.0625	0	0	0	0
249	1975	Male	76	237	FALSE	0	4.0517578	0.0419922	0.0186483	0.0081787	0
250	1957	Female	0	0	FALSE	0	0	0	0		
251	1988	Male	245	246	FALSE	0	4.5569422	0.0036439	0.0222793	0.0351563	0
252	1962	Female	0	0	FALSE	0	0	0	0		
253	1960	Female	244	241	FALSE	0	1	0	0	0	0
254	1965	Female	242	235	FALSE	0	2.125	0	0	0	0
255	1977	Male	238	234	FALSE	0	2.1875	0	0.015625	0.03125	0
256	1978	Female	228	239	FALSE	0	3.1607594	0	0.0018645	0	0.0010582
257	1980	Male	240	236	FALSE	0	2.34375	0	0	0	0
258	1968	Female	0	0	FALSE	0	0	0	0		
259	1965	Female	0	0	FALSE	0	0	0	0		
260	1986	Female	139	243	FALSE	0	3.9987488	0	0.040016	0	0.0010529
261	1965	Male	0	0	FALSE	0	0	0	0		
262	1975	Male	59	0	FALSE	0	1.5	0	0		0
263	1958	Female	0	0	FALSE	0	0	0	0		
264	1968	Female	247	250	FALSE	0	1.9375	0	0	0	0
265	1948	Female	0	0	FALSE	0	0	0	0		
266	1975	Female	248	258	FALSE	0	2.03125	0	0	0	0
267	1964	Male	251	256	FALSE	0	4.8588508	0.0643881	0.0138532	0	0.0036439
268	1965	Female	0	0	FALSE	0	0	0	0		
269	1954	Female	261	259	FALSE	0	1	0	0	0	0
270	1960	Male	0	0	FALSE	0	0	0	0		
271	1955	Male	0	0	FALSE	0	0	0	0		
272		Female	255	252	FALSE	0	2.09375	0	0.0078125	0	0
273	1958	Male	0	0	FALSE	0	0	0	0		
274	1981	Male	249	260	FALSE	0	5.0252533	0.0092254	0.0499367	0	0.0419922
275	1982	Male	0	0	FALSE	0	0	0	0		
276	1970	Female	0	0	FALSE	0	0	0	0		

277	1960	Male	257	253	FALSE	0	2.671875	0	0	0	0
278	1952	Male	0	0	FALSE	0	0	0	0		
279	1965	Female	0	0	FALSE	0	0	0	0		
280	1951	Female	0	0	FALSE	0	0	0	0		
281	1958	Male	0	0	FALSE	0	0	0	0		
282	1958	Female	71	254	FALSE	0	3.0625	0	0	0	0
283	1961	Female	0	0	FALSE	0	0	0	0		
284	1960	Female	0	0	FALSE	0	0	0	0		
285	1987	Female	0	0	FALSE	0	0	0	0		
286	1963	Male	181	263	FALSE	0	1	0	0	0	0
287	1965	Female	0	0	FALSE	0	0	0	0		
288	1985	Female	0	0	FALSE	0	0	0	0		
289	1963	Female	0	0	FALSE	0	0	0	0		
290	1975	Female	262	276	FALSE	0	1.75	0	0	0	0
291	1960	Male	0	0	FALSE	0	0	0	0		
292	1964	Female	0	0	FALSE	0	0	0	0		
293	1969	Female	0	0	FALSE	0	0	0	0		
294	1960	Female	273	268	FALSE	0	1	0	0	0	0
295	1963	Male	277	282	FALSE	0	3.8671875	0	0	0	0
296	1955	Male	278	280	FALSE	0	1	0	0	0	0
297	1960	Female	0	0	FALSE	0	0	0	0		
298	1963	Male	0	0	FALSE	0	0	0	0		
299	1965	Female	0	0	FALSE	0	0	0	0		
300	1976	Female	0	0	FALSE	0	0	0	0		
301	1965	Female	270	279	FALSE	0	1	0	0	0	0
302	1967	Female	281	264	FALSE	0	1.96875	0	0	0	0
303	1973	Female	0	0	FALSE	0	0	0	0		
304	1947	Female	0	0	FALSE	0	0	0	0		
305	1946	Male	0	0	FALSE	0	0	0	0		
306	1973	Male	0	0	FALSE	0	0	0	0		
307	1974	Male	267	266	FALSE	0	4.4450504	0.0027462	0.0386747	0	0.0643881
308	1965	Male	0	0	FALSE	0	0	0	0		
309	1962	Female	271	265	FALSE	0	1	0	0	0	0
310	1963	Male	0	0	FALSE	0	0	0	0		
311		Female	274	272	FALSE	0	4.5595016	0.0061955	0.0332569	0	0.0092254
312	1965	Male	275	269	FALSE	0	1.5	0	0	0	0
313	1959	Female	0	0	FALSE	0	0	0	0		
314	1968	Female	0	0	FALSE	0	0	0	0		
315	1963	Female	0	0	FALSE	0	0	0	0		
316	1968	Female	0	0	FALSE	0	0	0	0		
317	1958	Female	20	283	FALSE	0	1.875	0	0	0	0
318	1971	Female	50	0	FALSE	0	1	0	0		0
319	1979	Male	52	285	FALSE	0	2.0625	0	0	0	0
320	1960	Female	38	284	FALSE	0	1.5	0	0	0	0
321	1973	Female	0	0	FALSE	0	0	0	0		
322	1979	Male	59	0	FALSE	0	1.5	0	0		0
323	1958	Male	71	54	FALSE	0	3	0	0	0	0
324	1970	Female	198	0	FALSE	0	0.75	0	0		0
325	1974	Female	92	0	FALSE	0	2.078125	0	0		0
326	1975	Female	0	0	FALSE	0	0	0	0		
327	1996	Female	0	0	FALSE	0	0	0	0		
328	1958	Male	0	0	FALSE	0	0	0	0		
329	1965	Female	244	287	FALSE	0	1	0	0	0	0
330	1976	Female	247	288	FALSE	0	1.9375	0	0	0	0
331	1962	Female	0	0	FALSE	0	0	0	0		
332	1955	Male	74	254	FALSE	0	2.5625	0.0625	0	0	0

333	1967	Female	0	0	FALSE	0	0	0	0		
334	1967	Male	0	0	FALSE	0	0	0	0		
335	1950	Female	305	304	FALSE	0	1	0	0	0	0
336	1968	Male	0	0	FALSE	0	0	0	0		
337	1973	Male	0	0	FALSE	0	0	0	0		
338	1970	Male	307	309	FALSE	0	3.7225252	0	0.0206573	0	0.0027462
339	1975	Female	0	0	FALSE	0	0	0	0		
340	1976	Male	0	0	FALSE	0	0	0	0		
341	1977	Female	0	0	FALSE	0	0	0	0		
342	1968	Female	286	289	FALSE	0	1.5	0	0	0	0
343	1979	Female	0	0	FALSE	0	0	0	0		
344	1973	Female	291	311	FALSE	0	3.2797508	0	0.0196232	0.0061955	0
345	1970	Male	308	297	FALSE	0	1	0	0	0	0
346	1978	Female	219	290	FALSE	0	2.90625	0	0	0	0
347	1970	Male	298	292	FALSE	0	1	0	0	0	0
348	1958	Male	286	299	FALSE	0	1.5	0	0	0	0
349	1956	Female	312	300	FALSE	0	1.75	0	0	0	0
350	1956	Male	310	293	FALSE	0	1	0	0	0	0
351	1974	Male	295	87	FALSE	0	4.5585938	0.0634766	0	0	0
352	1970	Male	257	294	FALSE	0	2.671875	0	0	0	0
353	1959	Female	296	302	FALSE	0	2.484375	0	0	0	0
354	1954	Female	74	301	FALSE	0	2	0	0	0	0
355	1975	Male	0	0	FALSE	0	0	0	0		
356	1968	Male	0	0	FALSE	0	0	0	0		
357	1968	Female	0	0	FALSE	0	0	0	0		
358	1942	Male	0	0	FALSE	0	0	0	0		
359	1972	Female	0	0	FALSE	0	0	0	0		
360	1971	Male	306	303	FALSE	0	1	0	0	0	0
361	1947	Male	13	313	FALSE	0	1.25	0	0	0	0
362	1977	Female	0	0	FALSE	0	0	0	0		
363	1972	Male	30	314	FALSE	0	1	0	0	0	0
364	1980	Female	20	315	FALSE	0	1.875	0	0	0	0
365	1972	Female	0	0	FALSE	0	0	0	0		
366	1960	Female	48	316	FALSE	0	1.5	0	0	0	0
367	1979	Male	0	0	FALSE	0	0	0	0		
368	1970	Female	0	0	FALSE	0	0	0	0		
369	1970	Male	0	0	FALSE	0	0	0	0		
370	1977	Male	76	321	FALSE	0	2.03125	0	0	0	0
371	1978	Female	83	107	FALSE	0	3.53125	0	0.03125	0.0625	0
372	1982	Male	39	324	FALSE	0	1.375	0	0	0	0
373	1956	Female	0	0	FALSE	0	0	0	0		
374	1960	Female	0	0	FALSE	0	0	0	0		
375	1970	Male	0	0	FALSE	0	0	0	0		
376	1982	Female	0	0	FALSE	0	0	0	0		
377	1961	Female	0	0	FALSE	0	0	0	0		
378	1981	Female	153	327	FALSE	0	3.0934372	0	0.0573799	0	0.0669327
379	1984	Female	156	326	FALSE	0	1	0	0	0	0
380	1962	Female	328	167	FALSE	0	1.8046875	0	0.0039063	0	0
381	1970	Female	328	0	FALSE	0	0.5	0	0		0
382	1979	Male	286	318	FALSE	0	2	0	0	0	0
383	1975	Female	0	0	FALSE	0	0	0	0		
384	1970	Female	197	250	FALSE	0	1.375	0	0	0	0
385	1954	Female	74	329	FALSE	0	2	0	0	0	0
386	1966	Male	332	87	FALSE	0	3.90625	0.0019531	0.03125	0	0.0625
387	1975	Male	208	330	FALSE	0	2.59375	0	0	0	0
388	1939	Male	322	331	FALSE	0	1.75	0	0	0	0

389	1975	Male	0	0	FALSE	0	0	0	0		
390	1975	Female	0	0	FALSE	0	0	0	0		
391	1967	Male	0	0	FALSE	0	0	0	0		
392	1965	Female	0	0	FALSE	0	0	0	0		
393	1968	Male	355	349	FALSE	0	1.875	0	0	0	0
394	1966	Female	350	353	FALSE	0	2.7421875	0	0	0	0
395	1966	Male	0	0	FALSE	0	0	0	0		
396	1966	Male	0	0	FALSE	0	0	0	0		
397	1975	Male	0	0	FALSE	0	0	0	0		
398	1956	Male	0	0	FALSE	0	0	0	0		
399	1984	Female	351	325	FALSE	0	4.3183594	0.0030518	0.0317383	0	0.0634766
400	1966	Female	323	354	FALSE	0	3.5	0	0	0	0
401	1967	Female	0	0	FALSE	0	0	0	0		
402	1990	Male	0	0	FALSE	0	0	0	0		
403	1971	Female	356	357	FALSE	0	1	0	0	0	0
404	1957	Female	0	0	FALSE	0	0	0	0		
405	1970	Female	0	0	FALSE	0	0	0	0		
406	1947	Female	0	0	FALSE	0	0	0	0		
407	1963	Female	352	320	FALSE	0	3.0859375	0	0	0	0
408	1977	Male	360	359	FALSE	0	1.5	0	0	0	0
409	1970	Male	0	0	FALSE	0	0	0	0		
410	1988	Female	0	0	FALSE	0	0	0	0		
411	1950	Male	358	333	FALSE	0	1	0	0	0	0
412	1950	Female	0	0	FALSE	0	0	0	0		
413	1963	Female	0	0	FALSE	0	0	0	0		
414	1950	Male	0	0	FALSE	0	0	0	0		
415	1972	Male	0	0	FALSE	0	0	0	0		
416	1956	Male	0	0	FALSE	0	0	0	0		
417	1954	Female	340	341	FALSE	0	1	0	0	0	0
418	1970	Female	0	0	FALSE	0	0	0	0		
419	1978	Female	337	339	FALSE	0	1	0	0	0	0
420	1960	Female	0	0	FALSE	0	0	0	0		
421	1965	Male	336	317	FALSE	0	1.9375	0	0	0	0
422	1955	Male	0	0	FALSE	0	0	0	0		
423	1966	Male	0	0	FALSE	0	0	0	0		
424	1989	Female	0	0	FALSE	0	0	0	0		
425	1956	Male	20	335	FALSE	0	2.375	0	0	0	0
426	1978	Female	334	0	FALSE	0	0.5	0	0		0
427	1979	Male	127	346	FALSE	0	3.890625	0.0078125	0.03125	0	0.0625
428	1957	Female	338	344	FALSE	0	4.501138	0.0025137	0.0201403	0	0
429	1979	Female	0	0	FALSE	0	0	0	0		
430	1980	Male	0	0	FALSE	0	0	0	0		
431	1975	Female	0	0	FALSE	0	0	0	0		
432	1961	Female	0	0	FALSE	0	0	0	0		
433	1959	Male	20	335	FALSE	0	2.375	0	0	0	0
434		Male	0	0	FALSE	0	0	0	0		
435	1983	Female	319	343	FALSE	0	2.03125	0	0	0	0
436	1976	Male	0	0	FALSE	0	0	0	0		
437	1962	Male	348	342	FALSE	0	2.5	0.125	0	0	0
438	1985	Male	0	0	FALSE	0	0	0	0		
439	1976	Female	347	266	FALSE	0	2.515625	0	0	0	0
440	1964	Male	345	185	FALSE	0	3.4935913	0	0.0325347	0.0407715	0
441	1965	Female	0	0	FALSE	0	0	0	0		
442	1963	Female	0	313	FALSE	0	0.5	0	0	0	
443	1974	Female	0	0	FALSE	0	0	0	0		
444	1975	Female	20	0	FALSE	0	1.375	0	0		0

445	1960	Female	0	0	FALSE	0	0	0	0		
446	1959	Male	27	222	FALSE	0	1	0	0	0	0
447	1975	Female	27	0	FALSE	0	0.5	0	0		0
448	1985	Female	20	362	FALSE	0	1.875	0	0	0	0
449	1965	Female	0	0	FALSE	0	0	0	0		
450	1957	Female	44	0	FALSE	0	0.5	0	0		0
451	1975	Male	59	368	FALSE	0	2	0	0	0	0
452	1953	Female	363	366	FALSE	0	2.25	0	0	0	0
453	1977	Male	59	365	FALSE	0	2	0	0	0	0
454	1980	Male	367	58	FALSE	0	1.9375	0	0	0	0
455	1965	Female	0	0	FALSE	0	0	0	0		
456	1975	Male	369	81	FALSE	0	1.5	0	0	0	0
457	1972	Female	132	371	FALSE	0	4.265625	0.0351563	0.03125	0	0.03125
458	1958	Female	102	373	FALSE	0	1.5	0	0	0	0
459	1978	Female	108	374	FALSE	0	3.9287109	0	0.0337761	0	0.0010376
460	1956	Female	0	152	FALSE	0	1.71875	0	0.015625	0	
461	1975	Female	39	152	FALSE	0	2.21875	0	0.015625	0	0
462	1960	Male	375	143	FALSE	0	1	0	0	0	0
463	1975	Male	0	0	FALSE	0	0	0	0		
464	1984	Female	156	376	FALSE	0	1	0	0	0	0
465	1963	Male	157	377	FALSE	0	1.9375	0	0	0	0
466	1970	Female	0	0	FALSE	0	0	0	0		
467	1955	Male	20	167	FALSE	0	2.6796875	0.0234375	0.0039063	0	0
468	1980	Male	52	381	FALSE	0	2.3125	0	0	0	0
469	1975	Male	372	384	FALSE	0	2.375	0	0	0	0
470	1981	Female	207	383	FALSE	0	1.5	0	0	0	0
471	1961	Female	0	0	FALSE	0	0	0	0		
472	1959	Female	71	385	FALSE	0	3	0	0	0	0
473	1950	Male	388	119	FALSE	0	2.8125	0	0	0	0
474	1978	Male	387	81	FALSE	0	2.796875	0.03125	0	0	0
475	1970	Male	355	392	FALSE	0	1	0	0	0	0
476	1968	Female	0	0	FALSE	0	0	0	0		
477	1969	Male	360	364	FALSE	0	2.4375	0	0	0	0
478	1988	Male	389	353	FALSE	0	2.2421875	0	0	0	0
479	1965	Male	0	0	FALSE	0	0	0	0		
480	1975	Female	391	390	FALSE	0	1	0	0	0	0
481	1942	Male	0	0	FALSE	0	0	0	0		
482	1975	Male	0	0	FALSE	0	0	0	0		
483	1968	Male	0	0	FALSE	0	0	0	0		
484	1975	Male	438	429	FALSE	0	1	0	0	0	0
485	1982	Female	0	0	FALSE	0	0	0	0		
486	1966	Male	421	379	FALSE	0	2.46875	0	0	0	0
487	1966	Male	437	439	FALSE	0	3.5078125	0	0.0625	0	0.125
488	1968	Female	0	0	FALSE	0	0	0	0		
489	1946	Female	0	0	FALSE	0	0	0	0		
490	1946	Female	0	0	FALSE	0	0	0	0		
491	1972	Male	0	0	FALSE	0	0	0	0		
492	1975	Male	0	0	FALSE	0	0	0	0		
493	1947	Female	440	417	FALSE	0	3.2467957	0	0.0162673	0	0
494	1968	Female	332	407	FALSE	0	3.8242188	0	0.03125	0	0.0625
495		Male	0	0	FALSE	0	0	0	0		
496	1978	Female	397	431	FALSE	0	1	0	0	0	0
497	1972	Male	395	401	FALSE	0	1	0	0	0	0
498	1994	Female	427	435	FALSE	0	3.9609375	0.0126953	0.0194092	0	0.0078125
499	1970	Male	398	404	FALSE	0	1	0	0	0	0
500	1970	Female	0	0	FALSE	0	0	0	0		

501	1973	Male	382	394	FALSE	0	3.3710938	0	0	0	0
502	1971	Male	386	400	FALSE	0	4.703125	0.0742188	0.016571	0	0.0019531
503	1973	Female	393	403	FALSE	0	2.4375	0	0	0	0
504	1980	Male	396	0	FALSE	0	0.5	0	0		0
505		Female	0	406	FALSE	0	0.5	0	0	0	
506	1980	Male	0	0	FALSE	0	0	0	0		
507	1958	Male	0	0	FALSE	0	0	0	0		
508	1970	Female	402	399	FALSE	0	3.1591797	0	0.0173466	0.0030518	0
509	1956	Female	0	0	FALSE	0	0	0	0		
510	1954	Male	0	0	FALSE	0	0	0	0		
511	1955	Female	0	0	FALSE	0	0	0	0		
512	1980	Male	408	419	FALSE	0	2.25	0	0	0	0
513	1983	Female	0	0	FALSE	0	0	0	0		
514	1957	Male	414	412	FALSE	0	1	0	0	0	0
515	1981	Male	411	24	FALSE	0	2	0	0	0	0
516	1980	Female	415	418	FALSE	0	1	0	0	0	0
517	1980	Female	0	0	FALSE	0	0	0	0		
518	1969	Female	423	413	FALSE	0	1	0	0	0	0
519	1967	Female	422	428	FALSE	0	3.250569	0	0.0113017	0.0025137	0
520	1990	Female	370	424	FALSE	0	2.015625	0	0	0	0
521	1961	Female	416	432	FALSE	0	1	0	0	0	0
522	1962	Male	0	0	FALSE	0	0	0	0		
523	1962	Female	361	406	FALSE	0	1.625	0	0	0	0
524	1984	Male	425	426	FALSE	0	2.4375	0	0	0	0
525	1975	Female	409	405	FALSE	0	1	0	0	0	0
526	1974	Female	0	0	FALSE	0	0	0	0		
527	1985	Female	0	0	FALSE	0	0	0	0		
528	1965	Male	434	378	FALSE	0	2.5467186	0	0.02869	0	0
529	1986	Male	0	0	FALSE	0	0	0	0		
530	1985	Male	0	0	FALSE	0	0	0	0		
531	1964	Female	433	420	FALSE	0	2.1875	0	0	0	0
532	1968	Female	0	0	FALSE	0	0	0	0		
533	1963	Male	436	380	FALSE	0	1.9023438	0	0.0019531	0	0
534	1975	Female	430	410	FALSE	0	1	0	0	0	0
535	1965	Female	14	441	FALSE	0	1.5	0	0	0	0
536	1978	Female	20	443	FALSE	0	1.875	0	0	0	0
537	1970	Female	48	445	FALSE	0	1.5	0	0	0	0
538	1976	Male	127	442	FALSE	0	2.6875	0	0.03125	0	0.0625
539	1970	Male	0	0	FALSE	0	0	0	0		
540	1970	Male	59	449	FALSE	0	2	0	0	0	0
541	1972	Female	83	455	FALSE	0	2.40625	0	0	0	0
542	1978	Male	451	444	FALSE	0	2.6875	0	0	0	0
543	1970	Female	85	109	FALSE	0	3	0	0	0	0
544	1968	Female	56	458	FALSE	0	2.25	0.0625	0	0	0
545	1972	Female	85	459	FALSE	0	4.2143555	0.0229492	0.0168881	0	0
546	1979	Female	125	129	FALSE	0	4.4140625	0.0234375	0.0195313	0.03125	0.0078125
547	1980	Male	456	461	FALSE	0	2.859375	0	0.0078125	0	0
548	1956	Male	375	90	FALSE	0	2	0	0	0	0
549	1988	Female	0	0	FALSE	0	0	0	0		
550	1971	Female	0	0	FALSE	0	0	0	0		
551	1980	Female	463	152	FALSE	0	2.21875	0	0.015625	0	0
552	1980	Male	94	162	FALSE	0	3.6875	0.0175781	0.0390625	0	0.0625
553	1974	Male	172	466	FALSE	0	1.9375	0	0	0	0
554	1962	Male	467	450	FALSE	0	2.5898438	0	0.0136261	0	0.0234375
555	1976	Male	468	464	FALSE	0	2.65625	0	0	0	0
556	1970	Female	0	0	FALSE	0	0	0	0		

557	1960	Male	20	470	FALSE	0	2.625	0	0	0	0
558	1974	Male	469	63	FALSE	0	3.71875	0.0078125	0	0	0
559	1985	Female	0	0	FALSE	0	0	0	0		
560	1969	Male	247	471	FALSE	0	1.9375	0	0	0	0
561	1957	Female	71	254	FALSE	0	3.0625	0	0	0	0
562	1958	Female	20	269	FALSE	0	2.375	0	0	0	0
563	1960	Male	71	385	FALSE	0	3	0	0	0	0
564	1964	Female	85	472	FALSE	0	3.75	0.0625	0	0	0
565	1985	Male	312	448	FALSE	0	2.6875	0	0	0	0
566	1970	Female	473	0	FALSE	0	1.90625	0	0		0
567	1958	Female	391	317	FALSE	0	1.9375	0	0	0	0
568	1963	Female	71	354	FALSE	0	3	0	0	0	0
569	1991	Female	0	0	FALSE	0	0	0	0		
570	1975	Female	0	0	FALSE	0	0	0	0		
571	1980	Female	478	0	FALSE	0	1.6210938	0	0		0
572	1957	Male	475	236	FALSE	0	2.84375	0	0	0	0
573	1971	Female	361	476	FALSE	0	1.625	0	0	0	0
574	1975	Female	479	428	FALSE	0	3.250569	0	0.0113017	0.0025137	0
575	1966	Male	0	0	FALSE	0	0	0	0		
576	1966	Male	0	0	FALSE	0	0	0	0		
577	1980	Female	453	520	FALSE	0	3.0078125	0	0	0	0
578	1975	Female	502	452	FALSE	0	4.4765625	0.0117188	0.04478	0	0.0742188
579	1969	Male	0	0	FALSE	0	0	0	0		
580	1965	Male	446	519	FALSE	0	3.1252845	0.0019694	0.0056508	0	0
581	1975	Female	528	518	FALSE	0	2.7733593	0	0.014345	0	0
582	1975	Female	514	0	FALSE	0	1	0	0		0
583	1969	Male	219	523	FALSE	0	2.84375	0.015625	0	0	0
584	1960	Female	0	0	FALSE	0	0	0	0		
585		Female	0	0	FALSE	0	0	0	0		
586	1969	Female	462	489	FALSE	0	1.5	0	0	0	0
587		Male	0	0	FALSE	0	0	0	0		
588	1980	Male	482	447	FALSE	0	1.25	0	0	0	0
589	1970	Female	478	511	FALSE	0	2.1210938	0	0	0	0
590	1972	Male	465	521	FALSE	0	2.46875	0	0	0	0
591	1982	Female	454	485	FALSE	0	1.96875	0	0	0	0
592	1984	Female	515	0	FALSE	0	1.5	0	0		0
593	1994	Female	529	442	FALSE	0	1.25	0	0	0	0
594	1972	Female	522	509	FALSE	0	1	0	0	0	0
595	1971	Male	483	532	FALSE	0	1	0	0	0	0
596	1972	Female	524	523	FALSE	0	3.03125	0.015625	0	0	0
597	1972	Male	487	531	FALSE	0	3.8476563	0.0039063	0.03125	0	0
598	1980	Female	492	480	FALSE	0	1.5	0	0	0	0
599	1980	Female	372	525	FALSE	0	2.1875	0	0	0	0
600	1983	Female	468	517	FALSE	0	2.15625	0	0	0	0
601	1973	Female	533	0	FALSE	0	1.4511719	0	0.0009766		0
602	1973	Female	481	518	FALSE	0	1.5	0	0	0	0
603	1973	Female	0	0	FALSE	0	0	0	0		
604		Female	0	0	FALSE	0	0	0	0		
605	1978	Male	491	488	FALSE	0	1	0	0	0	0
606	1950	Female	0	0	FALSE	0	0	0	0		
607	1982	Female	474	516	FALSE	0	2.8984375	0	0.015625	0	0.03125
608	1975	Male	0	0	FALSE	0	0	0	0		
609	1973	Female	484	264	FALSE	0	2.46875	0	0	0	0
610	1975	Female	495	513	FALSE	0	1	0	0	0	0
611	1980	Male	0	0	FALSE	0	0	0	0		
612	1975	Male	477	534	FALSE	0	2.71875	0	0	0	0

613	1973	Female	530	527	FALSE	0	1	0	0	0	0
614	1976	Female	0	0	FALSE	0	0	0	0		
615	1980	Female	486	503	FALSE	0	3.453125	0	0	0	0
616	1955	Male	161	496	FALSE	0	3.2880859	0	0.0155555	0	0.015625
617	1978	Female	0	0	FALSE	0	0	0	0		
618	1978	Female	497	526	FALSE	0	1.5	0	0	0	0
619	1970	Female	0	0	FALSE	0	0	0	0		
620	1965	Female	0	498	FALSE	0	2.4804688	0	0.015929	0.0126953	
621	1968	Male	96	494	FALSE	0	4.6621094	0.0380859	0.046875	0	0.0625
622	1970	Male	0	0	FALSE	0	0	0	0		
623	1978	Female	0	0	FALSE	0	0	0	0		
624	1975	Male	473	508	FALSE	0	3.9858398	0.0029297	0.0086733	0	0
625	1976	Male	0	0	FALSE	0	0	0	0		
626	1980	Male	473	493	FALSE	0	4.0296478	0.0093689	0.0081337	0	0
627		Male	0	0	FALSE	0	0	0	0		
628	1980	Male	504	505	FALSE	0	1.5	0	0	0	0
629	1981	Male	0	0	FALSE	0	0	0	0		
630	1980	Female	499	380	FALSE	0	2.4023438	0	0.0019531	0	0
631	1983	Female	0	0	FALSE	0	0	0	0		
632	1962	Male	0	0	FALSE	0	0	0	0		
633	1968	Female	0	0	FALSE	0	0	0	0		
634	1960	Female	507	500	FALSE	0	1	0	0	0	0
635	1982	Female	506	0	FALSE	0	0.5	0	0		0
636	1962	Female	510	490	FALSE	0	1	0	0	0	0
637	1975	Female	0	0	FALSE	0	0	0	0		
638	1985	Male	0	0	FALSE	0	0	0	0		
639	1963	Female	0	0	FALSE	0	0	0	0		
640	1975	Male	0	0	FALSE	0	0	0	0		
641	1964	Male	0	0	FALSE	0	0	0	0		
642	1975	Female	512	460	FALSE	0	2.984375	0	0.0078125	0	0
643	1975	Male	0	0	FALSE	0	0	0	0		
644	1968	Male	501	457	FALSE	0	4.8183594	0.0040283	0.0326538	0.0351563	0
645		Female	0	0	FALSE	0	0	0	0		
646	1986	Male	20	0	FALSE	0	1.375	0	0		0
647	1960	Female	0	0	FALSE	0	0	0	0		
648	1975	Female	120	0	FALSE	0	1.4375	0	0		0
649	1954	Male	48	535	FALSE	0	2.25	0	0	0	0
650	1970	Female	0	0	FALSE	0	0	0	0		
651	1970	Female	59	21	FALSE	0	2.25	0	0	0	0
652	1971	Female	56	537	FALSE	0	2.25	0	0	0	0
653	1975	Female	540	204	FALSE	0	2.4375	0	0	0	0
654	1975	Female	539	81	FALSE	0	1.5	0	0	0	0
655	1968	Male	83	107	FALSE	0	3.53125	0	0.03125	0.0625	0
656	1975	Female	85	107	FALSE	0	3.375	0	0.03125	0.0625	0
657	1976	Male	132	541	FALSE	0	3.703125	0	0.015625	0	0.03125
658	1970	Female	108	86	FALSE	0	4.6787109	0.0400391	0.0337761	0	0.0010376
659	1965	Female	71	544	FALSE	0	3.125	0.03125	0.03125	0.0625	0
660	1973	Female	92	543	FALSE	0	4.078125	0.0078125	0	0	0
661	1977	Female	132	545	FALSE	0	4.6071777	0.0045166	0.0353499	0.0229492	0.03125
662	1968	Male	100	546	FALSE	0	4.8789063	0.0009766	0.048233	0.0234375	0.0234375
663	1980	Female	463	111	FALSE	0	1	0	0	0	0
664	1981	Female	153	154	FALSE	0	4.1557808	0.0708666	0.0623819	0	0.0669327
665	1980	Female	157	551	FALSE	0	3.046875	0.0234375	0.0078125	0	0
666	1996	Male	161	549	FALSE	0	2.7880859	0	0.0155555	0	0.015625
667	1975	Female	157	165	FALSE	0	2.6875	0	0	0	0
668	1980	Male	76	237	FALSE	0	4.0517578	0.0419922	0.0186483	0.0081787	0

669	1980	Male	181	536	FALSE	0	1.9375	0	0	0	0
670	1989	Female	0	0	FALSE	0	0	0	0		
671	1975	Female	198	556	FALSE	0	1.25	0	0	0	0
672	1971	Male	208	0	FALSE	0	1.125	0	0		0
673	1960	Female	0	0	FALSE	0	0	0	0		
674	1977	Male	207	58	FALSE	0	2.4375	0	0	0	0
675	1975	Female	219	0	FALSE	0	1.53125	0	0		0
676	1977	Male	558	0	FALSE	0	2.359375	0	0.0039063		0.0078125
677	1970	Female	247	559	FALSE	0	1.9375	0	0	0	0
678	1955	Female	74	329	FALSE	0	2	0	0	0	0
679	1966	Male	563	561	FALSE	0	4.03125	0.1328125	0	0	0
680	1958	Male	0	0	FALSE	0	0	0	0		
681	1957	Female	0	0	FALSE	0	0	0	0		
682	1974	Male	386	564	FALSE	0	4.828125	0.0996094	0.047821	0.0625	0.0019531
683	1969	Male	388	567	FALSE	0	2.84375	0	0	0	0
684	1971	Male	411	317	FALSE	0	2.4375	0	0	0	0
685	1969	Female	83	568	FALSE	0	3.90625	0	0	0	0
686	1975	Female	191	566	FALSE	0	3.1599246	0.0027761	0.0067935	0	0
687	1976	Female	0	0	FALSE	0	0	0	0		
688	1938	Male	0	0	FALSE	0	0	0	0		
689	1961	Male	0	0	FALSE	0	0	0	0		
690	1968	Male	0	0	FALSE	0	0	0	0		
691	1980	Male	52	525	FALSE	0	2.5625	0	0	0	0
692	1972	Male	487	562	FALSE	0	3.9414063	0.0078125	0.03125	0	0
693	1970	Male	468	569	FALSE	0	2.15625	0	0	0	0
694	1957	Male	76	574	FALSE	0	3.6565345	0.0135367	0.0056508	0	0
695	1985	Female	27	571	FALSE	0	1.8105469	0	0	0	0
696	1978	Female	475	570	FALSE	0	1.5	0	0	0	0
697	1973	Female	0	0	FALSE	0	0	0	0		
698	1974	Female	0	0	FALSE	0	0	0	0		
699	1972	Male	0	0	FALSE	0	0	0	0		
700	1983	Male	644	596	FALSE	0	4.9248047	0.0021973	0.0260878	0.015625	0.0040283
701	1984	Female	0	0	FALSE	0	0	0	0		
702	1981	Female	0	0	FALSE	0	0	0	0		
703	1989	Male	0	0	FALSE	0	0	0	0		
704	1967	Male	0	0	FALSE	0	0	0	0		
705	1975	Male	0	0	FALSE	0	0	0	0		
706	1990	Male	638	631	FALSE	0	1	0	0	0	0
707	1972	Female	0	0	FALSE	0	0	0	0		
708	1989	Female	0	0	FALSE	0	0	0	0		
709		Female	628	0	FALSE	0	1.25	0	0		0
710	1968	Female	52	636	FALSE	0	2.5625	0	0	0	0
711	1968	Male	0	633	FALSE	0	0.5	0	0	0	
712	1969	Male	641	639	FALSE	0	1	0	0	0	0
713	1974	Male	0	0	FALSE	0	0	0	0		
714		Male	0	0	FALSE	0	0	0	0		
715	1983	Female	0	0	FALSE	0	0	0	0		
716	1949	Female	548	634	FALSE	0	2.5	0	0	0	0
717	1973	Female	575	609	FALSE	0	2.234375	0	0	0	0
718	1985	Female	454	635	FALSE	0	2.21875	0	0	0	0
719	1993	Female	0	0	FALSE	0	0	0	0		
720		Female	627	585	FALSE	0	1	0	0	0	0
721	1994	Female	0	0	FALSE	0	0	0	0		
722	1980	Male	640	637	FALSE	0	1	0	0	0	0
723	1973	Female	629	550	FALSE	0	1	0	0	0	0
724	1980	Female	583	642	FALSE	0	3.9140625	0.0029297	0.0117188	0	0.015625

725	1975	Female	579	589	FALSE	0	2.0605469	0	0	0	0
726	1952	Male	557	584	FALSE	0	2.3125	0	0	0	0
727	1980	Female	643	582	FALSE	0	1.5	0	0	0	0
728	1953	Male	0	0	FALSE	0	0	0	0		
729	1955	Male	0	0	FALSE	0	0	0	0		
730	1976	Female	587	603	FALSE	0	1	0	0	0	0
731	1990	Female	0	0	FALSE	0	0	0	0		
732	1954	Male	547	599	FALSE	0	3.5234375	0.0351563	0.0039063	0	0
733	1977	Female	161	636	FALSE	0	3.2880859	0	0.0155555	0	0.015625
734	1955	Female	0	0	FALSE	0	0	0	0		
735	1978	Male	127	601	FALSE	0	3.1630859	0.0083008	0.0317383	0	0.0625
736	1957	Female	179	610	FALSE	0	3.2684326	0	0.0253306	0	0.0337372
737	1979	Male	560	602	FALSE	0	2.71875	0	0	0	0
738	1979	Female	554	586	FALSE	0	3.0449219	0	0.006813	0	0
739	1985	Male	588	598	FALSE	0	2.375	0	0	0	0
740	1985	Male	597	581	FALSE	0	4.3105078	0.0018649	0.0246896	0	0.0039063
741	1980	Female	538	594	FALSE	0	2.84375	0	0.015625	0	0
742	1980	Male	553	613	FALSE	0	2.46875	0	0	0	0
743	1987	Male	557	592	FALSE	0	3.0625	0	0	0	0
744	1990	Male	0	0	FALSE	0	0	0	0		
745		Female	632	604	FALSE	0	1	0	0	0	0
746	1985	Male	608	617	FALSE	0	1	0	0	0	0
747	1982	Female	555	614	FALSE	0	2.328125	0	0	0	0
748	1975	Male	622	619	FALSE	0	1	0	0	0	0
749		Female	616	606	FALSE	0	2.644043	0	0.0077778	0	0
750	1987	Female	565	607	FALSE	0	3.7929688	0.0039063	0.0078125	0	0
751	1985	Female	360	600	FALSE	0	2.578125	0	0	0	0
752	1982	Female	612	593	FALSE	0	2.984375	0	0	0	0
753	1985	Female	605	591	FALSE	0	2.484375	0	0	0	0
754	1976	Female	542	577	FALSE	0	3.8476563	0.0351563	0	0	0
755	1984	Female	572	609	FALSE	0	3.65625	0.0078125	0	0	0
756	1970	Male	20	620	FALSE	0	3.1152344	0.0410156	0.0079645	0	0
757	1984	Male	597	256	FALSE	0	4.5042078	0.0015861	0.0184493	0	0.0039063
758	1984	Male	0	0	FALSE	0	0	0	0		
759	1982	Male	552	615	FALSE	0	4.5703125	0.0229492	0.027977	0	0.0175781
760	1963	Male	626	428	FALSE	0	5.2653929	0.0049854	0.0200149	0.0025137	0.0093689
761	1985	Female	580	160	FALSE	0	4.6405326	0.0021395	0.0682184	0.0708666	0.0019694
762	1985	Female	611	566	FALSE	0	1.953125	0	0	0	0
763	1964	Female	576	645	FALSE	0	1	0	0	0	0
764	1982	Female	624	618	FALSE	0	3.7429199	0	0.0057888	0	0.0029297
765	1986	Female	590	630	FALSE	0	3.4355469	0.0007324	0.0009766	0	0
766	1985	Female	625	623	FALSE	0	1	0	0	0	0
767	1965	Female	0	0	FALSE	0	0	0	0		
768	1977	Female	595	573	FALSE	0	2.3125	0	0	0	0
769	1982	Male	621	578	FALSE	0	5.5693359	0.055603	0.0695748	0.0117188	0.0380859
770	1975	Female	52	58	FALSE	0	3	0.125	0	0	0
771	1965	Female	48	647	FALSE	0	1.5	0	0	0	0
772	1973	Female	48	19	FALSE	0	2.5	0	0	0	0
773	1974	Female	56	0	FALSE	0	1	0	0		0
774	1975	Female	76	650	FALSE	0	2.03125	0	0	0	0
775	1978	Male	80	648	FALSE	0	2.78125	0.03125	0	0	0
776	1978	Female	92	652	FALSE	0	3.703125	0.0625	0	0	0
777	1975	Female	132	656	FALSE	0	4.1875	0.0351563	0.03125	0	0.03125
778	1970	Female	0	0	FALSE	0	0	0	0		
779	1975	Female	0	0	FALSE	0	0	0	0		
780	1980	Female	85	658	FALSE	0	4.5893555	0.0229492	0.0362314	0.0400391	0

781	1970	Female	655	658	FALSE	0	5.1049805	0.038269	0.0518564	0.0400391	0
782	1987	Female	92	116	FALSE	0	5.1049805	0.1047974	0.043698	0.0239258	0
783	1968	Female	92	659	FALSE	0	4.140625	0.0429688	0.0307617	0.03125	0
784	1978	Female	125	656	FALSE	0	4.7265625	0.1152344	0.0195313	0	0.0078125
785	1987	Female	662	661	FALSE	0	5.743042	0.0430012	0.0444346	0.0045166	0.0009766
786	1978	Male	157	663	FALSE	0	2.4375	0	0	0	0
787	1973	Female	161	550	FALSE	0	2.7880859	0	0.0155555	0	0.015625
788	1978	Male	161	653	FALSE	0	4.0068359	0.0161133	0.0155555	0	0.015625
789		Female	0	0	FALSE	0	0	0	0		
790	1978	Male	468	0	FALSE	0	1.65625	0	0		0
791	1979	Female	249	0	FALSE	0	2.5258789	0	0.0299287		0.0419922
792	1982	Male	249	134	FALSE	0	4.4946289	0.0283203	0.0455537	0	0.0419922
793	1985	Male	208	670	FALSE	0	1.625	0	0	0	0
794	1954	Male	76	671	FALSE	0	2.65625	0	0	0	0
795		Female	0	210	FALSE	0	0.5	0	0	0	
796	1966	Male	219	673	FALSE	0	2.03125	0	0	0	0
797	1977	Male	127	675	FALSE	0	3.203125	0.0078125	0.03125	0	0.0625
798	1982	Male	558	0	FALSE	0	2.359375	0	0.0039063		0.0078125
799	1984	Female	676	0	FALSE	0	1.6796875	0	0.0019531		0
800	1965	Female	0	0	FALSE	0	0	0	0		
801	1961	Male	649	678	FALSE	0	3.125	0	0	0	0
802	1972	Female	0	0	FALSE	0	0	0	0		
803	1960	Male	71	354	FALSE	0	3	0	0	0	0
804	1970	Female	680	317	FALSE	0	1.9375	0	0	0	0
805	1960	Female	323	681	FALSE	0	2.5	0	0	0	0
806	1977	Female	338	169	FALSE	0	4.2553056	0.0055575	0.0181064	0	0
807	1977	Female	0	0	FALSE	0	0	0	0		
808	1977	Female	0	0	FALSE	0	0	0	0		
809	1982	Female	565	205	FALSE	0	2.34375	0	0	0	0
810	1972	Female	0	0	FALSE	0	0	0	0		
811		Female	0	0	FALSE	0	0	0	0		
812	1984	Female	682	656	FALSE	0	5.1015625	0.0976563	0.0869585	0	0.0996094
813	1987	Female	433	651	FALSE	0	3.3125	0	0	0	0
814	1982	Male	502	98	FALSE	0	5.0742188	0	0.0486862	0	0.0742188
815	1982	Female	502	660	FALSE	0	5.390625	0.0302734	0.0486862	0.0078125	0.0742188
816	1968	Male	689	521	FALSE	0	1.5	0	0	0	0
817	1975	Male	690	521	FALSE	0	1.5	0	0	0	0
818	1995	Female	646	687	FALSE	0	1.6875	0	0	0	0
819	1975	Male	473	493	FALSE	0	4.0296478	0.0093689	0.0081337	0	0
820	1977	Male	497	63	FALSE	0	3.03125	0	0	0	0
821	1981	Female	0	0	FALSE	0	0	0	0		
822	1975	Female	679	685	FALSE	0	4.96875	0.0722656	0.0664063	0	0.1328125
823	1958	Female	688	511	FALSE	0	1	0	0	0	0
824	1984	Female	684	686	FALSE	0	3.7987123	0.0103714	0.0047754	0.0027761	0
825	1987	Female	644	596	FALSE	0	4.9248047	0.0021973	0.0260878	0.015625	0.0040283
826		Female	0	0	FALSE	0	0	0	0		
827	1978	Female	0	0	FALSE	0	0	0	0		
828	1985	Female	691	665	FALSE	0	3.8046875	0.0371094	0.0155334	0.0234375	0
829	1975	Female	693	677	FALSE	0	3.046875	0.015625	0	0	0
830		Female	580	0	FALSE	0	2.0626423	0	0.0038045		0.0019694
831	1988	Male	692	667	FALSE	0	4.3144531	0.0332031	0.0194092	0	0.0078125
832	1981	Female	0	0	FALSE	0	0	0	0		
833	1982	Female	611	0	FALSE	0	0.5	0	0		0
834	1980	Male	612	609	FALSE	0	3.59375	0.0078125	0	0	0
835		Female	0	0	FALSE	0	0	0	0		
836	1986	Female	427	696	FALSE	0	3.6953125	0	0.0194092	0	0.0078125

837	1987	Female	700	701	FALSE	0	3.4624023	0	0.0141139	0	0.0021973
838	1987	Female	626	753	FALSE	0	4.2570114	0.0050344	0.0087132	0	0.0093689
839	1966	Male	0	0	FALSE	0	0	0	0		
840	1989	Female	757	765	FALSE	0	4.9698773	0.0028568	0.0108572	0.0007324	0.0015861
841	1993	Male	669	750	FALSE	0	3.8652344	0.0175781	0.0058441	0.0039063	0
842	1989	Male	746	766	FALSE	0	2	0	0	0	0
843	1986	Female	657	715	FALSE	0	2.8515625	0	0.0078125	0	0
844	1990	Male	427	754	FALSE	0	4.8691406	0.0220947	0.0369873	0.0351563	0.0078125
845	1946	Female	0	0	FALSE	0	0	0	0		
846	1990	Female	759	764	FALSE	0	5.1566162	0.0063515	0.0280365	0	0.0229492
847	1969	Male	0	0	FALSE	0	0	0	0		
848	1992	Female	759	762	FALSE	0	4.2617188	0.0061035	0.0251421	0	0.0229492
849	1957	Female	0	0	FALSE	0	0	0	0		
850	1986	Male	644	741	FALSE	0	4.8310547	0.0008545	0.0260878	0	0.0040283
851	1980	Female	748	654	FALSE	0	2.25	0	0	0	0
852	1977	Female	699	697	FALSE	0	1	0	0	0	0
853	1993	Female	703	721	FALSE	0	1	0	0	0	0
854	1993	Female	705	762	FALSE	0	1.9765625	0	0	0	0
855	1995	Female	0	702	FALSE	0	0.5	0	0	0	
856	1973	Male	704	767	FALSE	0	1	0	0	0	0
857	1975	Female	0	0	FALSE	0	0	0	0		
858	1965	Female	758	0	FALSE	0	0.5	0	0		0
859	1974	Male	0	0	FALSE	0	0	0	0		
860	1980	Female	713	707	FALSE	0	1	0	0	0	0
861	1978	Female	713	755	FALSE	0	2.828125	0	0.0039063	0.0078125	0
862	1975	Female	714	709	FALSE	0	1.625	0	0	0	0
863	1975	Female	712	717	FALSE	0	2.6171875	0	0	0	0
864	1976	Male	706	708	FALSE	0	1.5	0	0	0	0
865	1998	Male	756	719	FALSE	0	2.5576172	0	0.0243267	0	0.0410156
866	1977	Female	0	0	FALSE	0	0	0	0		
867	1977	Male	0	0	FALSE	0	0	0	0		
868	1981	Female	711	508	FALSE	0	2.8295898	0	0.0086733	0	0
869	1965	Female	0	0	FALSE	0	0	0	0		
870	1965	Female	0	0	FALSE	0	0	0	0		
871	1987	Female	0	0	FALSE	0	0	0	0		
872	1957	Male	0	0	FALSE	0	0	0	0		
873	1983	Female	674	768	FALSE	0	3.375	0.0078125	0	0	0
874		Female	0	0	FALSE	0	0	0	0		
875	1985	Male	722	727	FALSE	0	2.25	0	0	0	0
876	1978	Female	0	0	FALSE	0	0	0	0		
877	1979	Female	683	763	FALSE	0	2.921875	0	0	0	0
878	1980	Female	692	723	FALSE	0	3.4707031	0	0.0194092	0	0.0078125
879	1980	Female	726	716	FALSE	0	3.40625	0	0	0	0
880	1980	Female	672	710	FALSE	0	2.84375	0	0	0	0
881	1988	Male	760	761	FALSE	0	5.9529628	0.040494	0.0475562	0.0021395	0.0049854
882		Male	0	0	FALSE	0	0	0	0		
883	1979	Male	0	0	FALSE	0	0	0	0		
884	1978	Female	0	0	FALSE	0	0	0	0		
885	1960	Female	728	734	FALSE	0	1	0	0	0	0
886	1968	Female	76	725	FALSE	0	3.0615234	0.0009766	0	0	0
887		Male	729	0	FALSE	0	0.5	0	0		0
888	1982	Male	0	0	FALSE	0	0	0	0		
889	1983	Female	0	0	FALSE	0	0	0	0		
890	1988	Female	735	718	FALSE	0	3.690918	0.0119019	0.0198878	0	0.0083008
891		Male	732	0	FALSE	0	2.2617188	0	0.0194626		0.0351563
892	1963	Male	0	0	FALSE	0	0	0	0		

893	1984	Female	626	730	FALSE	0	3.5148239	0	0.0087132	0	0.0093689
894	1984	Female	666	698	FALSE	0	2.394043	0	0.0077778	0	0
895	1988	Female	769	661	FALSE	0	6.0882568	0.0342388	0.080508	0.0045166	0.055603
896	1985	Female	737	738	FALSE	0	3.8818359	0.0081787	0.0034065	0	0
897	1985	Female	370	745	FALSE	0	2.515625	0	0	0	0
898	1985	Female	694	736	FALSE	0	4.4624836	0.0119221	0.0222208	0	0.0135367
899	1985	Male	338	733	FALSE	0	4.5053056	0.0055404	0.0181064	0	0
900		Female	0	0	FALSE	0	0	0	0		
901	1962	Male	0	0	FALSE	0	0	0	0		
902		Male	555	751	FALSE	0	3.6171875	0.0625	0	0	0
903	1986	Male	735	743	FALSE	0	4.112793	0.0238037	0.0198878	0	0.0083008
904	1985	Female	692	720	FALSE	0	3.4707031	0	0.0194092	0	0.0078125
905		Female	744	731	FALSE	0	1	0	0	0	0
906	1964	Male	735	353	FALSE	0	3.8237305	0.0059509	0.0198878	0	0.0083008
907	1986	Male	742	752	FALSE	0	3.7265625	0.0039063	0	0	0
908	1986	Female	740	664	FALSE	0	5.2331443	0.0228323	0.0776681	0.0708666	0.0018649
909	1986	Male	552	724	FALSE	0	4.8007813	0.0236816	0.035284	0.0029297	0.0175781
910	1990	Female	739	695	FALSE	0	3.0927734	0.03125	0	0	0
911		Male	668	749	FALSE	0	4.3479004	0.0335197	0.0338176	0	0.0419922
912	1987	Female	512	747	FALSE	0	3.2890625	0	0	0	0
913	1965	Female	71	772	FALSE	0	3.25	0	0	0	0
914	1973	Female	71	773	FALSE	0	2.5	0.03125	0	0	0
915	1967	Female	56	771	FALSE	0	2.25	0	0	0	0
916		Female	80	0	FALSE	0	1.5625	0	0		0
917	1976	Female	132	656	FALSE	0	4.1875	0.0351563	0.03125	0	0.03125
918	1976	Female	132	776	FALSE	0	4.3515625	0.0585938	0.046875	0.0625	0.03125
919	1975	Female	108	778	FALSE	0	3.9287109	0	0.0337761	0	0.0010376
920	1972	Female	655	543	FALSE	0	4.265625	0.0351563	0.015625	0	0
921	1974	Female	117	779	FALSE	0	2.125	0	0.03125	0	0.0625
922	1971	Female	0	0	FALSE	0	0	0	0		
923	1985	Female	100	660	FALSE	0	4.7109375	0.0019531	0.0308838	0.0078125	0.0234375
924	1981	Male	132	782	FALSE	0	5.0524902	0.041626	0.087583	0.1047974	0.03125
925	1975	Female	132	781	FALSE	0	5.0524902	0.041626	0.0596955	0.038269	0.03125
926	1977	Female	132	781	FALSE	0	5.0524902	0.041626	0.0596955	0.038269	0.03125
927	1968	Female	132	780	FALSE	0	4.7946777	0.0240479	0.0447996	0.0229492	0.03125
928	1980	Female	132	545	FALSE	0	4.6071777	0.0045166	0.0353499	0.0229492	0.03125
929	1981	Female	132	783	FALSE	0	4.5703125	0.109375	0.0518293	0.0429688	0.03125
930	1986	Female	662	777	FALSE	0	5.5332031	0.0427246	0.0572351	0.0351563	0.0009766
931	1979	Female	547	0	FALSE	0	1.9296875	0	0.0039063		0
932		Female	0	0	FALSE	0	0	0	0		
933	1980	Female	161	653	FALSE	0	4.0068359	0.0161133	0.0155555	0	0.015625
934	1986	Female	0	0	FALSE	0	0	0	0		
935		Female	179	789	FALSE	0	2.7684326	0	0.0253306	0	0.0337372
936	1982	Female	797	787	FALSE	0	3.9956055	0.0261841	0.0271869	0	0.0078125
937	1969	Male	277	800	FALSE	0	2.3359375	0	0	0	0
938	1982	Female	360	536	FALSE	0	2.4375	0	0	0	0
939	1970	Female	345	802	FALSE	0	1.5	0	0	0	0
940	1988	Female	792	809	FALSE	0	4.4191895	0.0140991	0.0362919	0	0.0283203
941	1988	Male	427	799	FALSE	0	3.7851563	0.0056152	0.0203857	0	0.0078125
942	1989	Female	427	651	FALSE	0	4.0703125	0.015625	0.0194092	0	0.0078125
943	1965	Female	803	805	FALSE	0	3.75	0.0625	0	0	0
944	1980	Female	803	407	FALSE	0	4.0429688	0.03125	0	0	0
945	1995	Female	0	0	FALSE	0	0	0	0		
946	1970	Female	0	0	FALSE	0	0	0	0		
947	1980	Female	0	0	FALSE	0	0	0	0		
948	1983	Male	370	807	FALSE	0	2.015625	0	0	0	0

949	1985	Male	396	808	FALSE	0	1	0	0	0	0
950	1960	Male	502	452	FALSE	0	4.4765625	0.0117188	0.04478	0	0.0742188
951	1980	Female	502	452	FALSE	0	4.4765625	0.0117188	0.04478	0	0.0742188
952	1975	Female	486	152	FALSE	0	3.453125	0.0117188	0.015625	0	0
953	1977	Female	468	810	FALSE	0	2.15625	0	0	0	0
954	1980	Female	684	0	FALSE	0	1.71875	0	0		0
955		Female	475	811	FALSE	0	1.5	0	0	0	0
956	1989	Female	819	795	FALSE	0	3.2648239	0	0.0087132	0	0.0093689
957	1969	Female	796	823	FALSE	0	2.515625	0	0	0	0
958		Female	0	0	FALSE	0	0	0	0		
959	1987	Female	644	98	FALSE	0	5.1318359	0.0014648	0.0221815	0	0.0040283
960	1993	Male	427	824	FALSE	0	4.8446686	0.0235091	0.0269578	0.0103714	0.0078125
961	1983	Male	644	545	FALSE	0	5.5163574	0.0031929	0.0380001	0.0229492	0.0040283
962	1989	Female	644	815	FALSE	0	6.1044922	0.0273743	0.0570182	0.0302734	0.0040283
963	1980	Male	817	770	FALSE	0	3.25	0	0.0625	0.125	0
964		Female	0	0	FALSE	0	0	0	0		
965	1978	Female	605	774	FALSE	0	2.515625	0	0	0	0
966	1979	Female	801	822	FALSE	0	5.046875	0.0258789	0.0669365	0.0722656	0
967	1983	Female	611	821	FALSE	0	1	0	0	0	0
968	1982	Female	668	609	FALSE	0	4.2602539	0.0164795	0.0299287	0	0.0419922
969	1984	Female	387	818	FALSE	0	3.140625	0.015625	0	0	0
970	1983	Male	597	256	FALSE	0	4.5042078	0.0015861	0.0184493	0	0.0039063
971	1985	Male	820	806	FALSE	0	4.6432778	0.0124893	0.0117816	0.0055575	0
972	1987	Female	788	603	FALSE	0	3.003418	0	0.0157091	0	0.0161133
973	1990	Female	769	784	FALSE	0	6.1479492	0.0301666	0.1269121	0.1152344	0.055603
974	1988	Male	732	738	FALSE	0	4.2841797	0.003418	0.0228691	0	0.0351563
975		Male	740	160	FALSE	0	5.2331443	0.0228323	0.0776681	0.0708666	0.0018649
976	1989	Male	0	0	FALSE	0	0	0	0		
977	1989	Male	427	755	FALSE	0	4.7734375	0.0126953	0.0233154	0.0078125	0.0078125
978	1989	Female	834	828	FALSE	0	4.6992188	0.0170898	0.0299394	0.0371094	0.0078125
979	1991	Female	814	825	FALSE	0	5.9995117	0.0083008	0.038457	0.0021973	0
980	1978	Female	684	835	FALSE	0	2.21875	0	0	0	0
981		Male	684	826	FALSE	0	2.21875	0	0	0	0
982		Male	739	695	FALSE	0	3.0927734	0.03125	0	0	0
983	1980	Male	624	829	FALSE	0	4.5163574	0.012085	0.0136013	0.015625	0.0029297
984	1985	Female	726	827	FALSE	0	2.15625	0	0	0	0
985	1985	Female	740	664	FALSE	0	5.2331443	0.0228323	0.0776681	0.0708666	0.0018649
986		Female	558	830	FALSE	0	3.8906961	0.001178	0.0058085	0	0.0078125
987	1990	Female	732	832	FALSE	0	2.7617188	0	0.0194626	0	0.0351563
988	1985	Male	798	833	FALSE	0	2.4296875	0	0.0019531	0	0
989	1967	Male	901	870	FALSE	0	1	0	0	0	0
990	1988	Female	864	889	FALSE	0	1.75	0	0	0	0
991	1988	Female	0	0	FALSE	0	0	0	0		
992	1988	Male	427	860	FALSE	0	3.4453125	0	0.0194092	0	0.0078125
993	1966	Male	208	863	FALSE	0	2.9335938	0	0	0	0
994	1988	Male	612	868	FALSE	0	3.7741699	0.0002441	0.0043366	0	0
995	1989	Female	612	894	FALSE	0	3.5563965	0.0080566	0.0038889	0	0
996	1992	Male	859	791	FALSE	0	2.2629395	0	0.0149643	0	0
997	1989	Female	831	874	FALSE	0	3.1572266	0	0.0259839	0	0.0332031
998	1989	Male	0	0	FALSE	0	0	0	0		
999	1991	Female	881	785	FALSE	0	6.8480024	0.0064511	0.0858248	0.0430012	0.040494
1000	1989	Female	737	879	FALSE	0	4.0625	0.0078125	0	0	0
1001	1989	Male	0	0	FALSE	0	0	0	0		
1002	1991	Male	850	812	FALSE	0	5.9663086	0.0147705	0.1015214	0.0976563	0.0008545
1003		Female	882	891	FALSE	0	2.1308594	0	0.0097313	0	0
1004	1984	Female	100	877	FALSE	0	4.1328125	0.0078125	0.0269775	0	0.0234375

1005	1968	Female	816	845	FALSE	0	1.75	0	0	0	0
1006	1992	Female	881	895	FALSE	0	7.0206098	0.0017229	0.0990574	0.0342388	0.040494
1007	1991	Female	0	0	FALSE	0	0	0	0		
1008	1969	Female	887	0	FALSE	0	0.75	0	0		0
1009	1991	Male	427	912	FALSE	0	4.5898438	0.0031738	0.0194092	0	0.0078125
1010	1991	Female	740	905	FALSE	0	3.6552539	0	0.0132542	0	0.0018649
1011	1988	Female	558	890	FALSE	0	4.704834	0.0128174	0.0196827	0.0119019	0.0078125
1012	1991	Male	245	893	FALSE	0	4.4627916	0.0013251	0.0052888	0	0
1013	1991	Female	909	837	FALSE	0	5.1315918	0.0119133	0.036122	0	0.0236816
1014	1992	Male	739	897	FALSE	0	3.4453125	0	0	0	0
1015	1992	Female	0	0	FALSE	0	0	0	0		
1016	1991	Female	899	843	FALSE	0	4.678434	0.001957	0.0156795	0	0.0055404
1017	1992	Female	0	0	FALSE	0	0	0	0		
1018		Male	902	838	FALSE	0	4.9370995	0.0053627	0.0381019	0.0050344	0.0625
1019	1971	Male	486	885	FALSE	0	2.734375	0	0	0	0
1020	1993	Male	0	0	FALSE	0	0	0	0		
1021		Female	903	813	FALSE	0	4.7126465	0.0275269	0.0216091	0	0.0238037
1022	1994	Female	907	836	FALSE	0	4.7109375	0.0047607	0.0116577	0	0.0039063
1023	1994	Male	831	910	FALSE	0	4.7036133	0.0005035	0.0416089	0.03125	0.0332031
1024	1995	Male	0	0	FALSE	0	0	0	0		
1025	1997	Female	909	898	FALSE	0	5.6316324	0.0163569	0.0460041	0.0119221	0.0236816
1026	1995	Female	841	908	FALSE	0	5.5491893	0.0115198	0.0610233	0.0228323	0.0175781
1027	1995	Male	842	846	FALSE	0	4.5783081	0	0.0171049	0.0063515	0
1028	1998	Female	911	904	FALSE	0	4.9093018	0.0137844	0.0428065	0	0.0335197
1029	1996	Male	844	848	FALSE	0	5.5654297	0.0168304	0.0446785	0.0061035	0.0220947
1030	1996	Male	888	900	FALSE	0	1	0	0	0	0
1031		Male	0	0	FALSE	0	0	0	0		
1032		Female	0	0	FALSE	0	0	0	0		
1033	1960	Female	0	0	FALSE	0	0	0	0		
1034	1967	Female	901	870	FALSE	0	1	0	0	0	0
1035	1991	Male	899	896	FALSE	0	5.1935708	0.0106385	0.0175519	0.0081787	0.0055404
1036	1960	Male	872	849	FALSE	0	1	0	0	0	0
1037	1980	Female	790	861	FALSE	0	3.2421875	0.0039063	0.0019531	0	0
1038	1980	Male	856	804	FALSE	0	2.46875	0	0	0	0
1039	1983	Female	865	853	FALSE	0	2.7788086	0	0.0121634	0	0
1040	1990	Male	906	765	FALSE	0	4.6296387	0.0139313	0.0137143	0.0007324	0.0059509
1041	1980	Male	794	855	FALSE	0	2.578125	0	0	0	0
1042	1981	Female	228	862	FALSE	0	3.4732594	0	0.0018645	0	0.0010582
1043	1981	Female	370	852	FALSE	0	2.515625	0	0	0	0
1044	1982	Female	676	858	FALSE	0	2.4296875	0	0.0019531	0	0
1045	1982	Female	0	0	FALSE	0	0	0	0		
1046	1982	Female	839	0	FALSE	0	0.5	0	0		0
1047	1982	Male	794	866	FALSE	0	2.328125	0	0	0	0
1048		Female	793	871	FALSE	0	1.8125	0	0	0	0
1049	1983	Female	864	428	FALSE	0	4.000569	0	0.0113017	0.0025137	0
1050	1984	Female	786	857	FALSE	0	2.21875	0	0	0	0
1051	1967	Female	892	869	FALSE	0	1	0	0	0	0
1052	1983	Male	558	854	FALSE	0	3.8476563	0.0024414	0.0039063	0	0.0078125
1053	1982	Male	0	0	FALSE	0	0	0	0		
1054	1984	Female	847	876	FALSE	0	1	0	0	0	0
1055	1972	Male	0	0	FALSE	0	0	0	0		
1056	1975	Female	0	0	FALSE	0	0	0	0		
1057	1985	Male	775	880	FALSE	0	3.8125	0.0195313	0.015625	0	0.03125
1058	1992	Male	909	840	FALSE	0	5.8853293	0.0142442	0.0359066	0.0028568	0.0236816
1059		Female	794	851	FALSE	0	3.453125	0.0078125	0	0	0
1060	1986	Male	477	886	FALSE	0	3.7495117	0.0161133	0.0004883	0.0009766	0

1061	1980	Female	867	884	FALSE	0	1	0	0	0	0
1062	1988	Female	245	873	FALSE	0	4.3928797	0.0024917	0.0048385	0.0078125	0
1063	1986	Female	427	860	FALSE	0	3.4453125	0	0.0194092	0	0.0078125
1064	1987	Male	883	878	FALSE	0	2.7353516	0	0.0097046	0	0
1065	1990	Male	875	182	FALSE	0	4.4466107	0	0.0304849	0.0020829	0
1066	1965	Female	0	0	FALSE	0	0	0	0		
1067	1975	Female	0	0	FALSE	0	0	0	0		
1068	1965	Female	0	0	FALSE	0	0	0	0		
1069	1978	Female	71	915	FALSE	0	3.125	0.03125	0	0	0
1070	1960	Female	649	235	FALSE	0	3.25	0.015625	0	0	0
1071	1977	Male	96	91	FALSE	0	4.5625	0.078125	0.0390625	0.015625	0.0625
1072	1980	Female	92	914	FALSE	0	3.828125	0.0390625	0.015625	0.03125	0
1073	1965	Female	0	0	FALSE	0	0	0	0		
1074	1979	Female	655	919	FALSE	0	4.7299805	0.0148315	0.0325131	0	0
1075	1973	Female	132	658	FALSE	0	4.8393555	0.0480957	0.0518564	0.0400391	0.03125
1076	1973	Female	92	116	FALSE	0	5.1049805	0.1047974	0.043698	0.0239258	0
1077	1982	Female	125	922	FALSE	0	3.0390625	0	0.0039063	0	0.0078125
1078	1978	Male	132	545	FALSE	0	4.6071777	0.0045166	0.0353499	0.0229492	0.03125
1079	1980	Male	132	545	FALSE	0	4.6071777	0.0045166	0.0353499	0.0229492	0.03125
1080	1980	Female	132	780	FALSE	0	4.7946777	0.0240479	0.0447996	0.0229492	0.03125
1081	1981	Female	132	921	FALSE	0	3.5625	0.046875	0.03125	0	0.03125
1082	1985	Male	100	927	FALSE	0	5.0692139	0.01651	0.0608626	0.0240479	0.0234375
1083	1984	Female	131	129	FALSE	0	5.0574951	0.0422974	0.0883128	0.03125	0.0673981
1084	1986	Female	131	129	FALSE	0	5.0574951	0.0422974	0.0883128	0.03125	0.0673981
1085	1987	Female	662	928	FALSE	0	5.743042	0.0430012	0.0444346	0.0045166	0.0009766
1086	1981	Female	153	932	FALSE	0	3.0934372	0	0.0573799	0	0.0669327
1087	1970	Female	0	0	FALSE	0	0	0	0		
1088	1980	Female	164	934	FALSE	0	1.71875	0	0	0	0
1089	1988	Male	668	787	FALSE	0	4.4199219	0.0670395	0.0377064	0	0.0419922
1090	1982	Female	555	536	FALSE	0	3.265625	0.03125	0	0	0
1091	1985	Female	558	185	FALSE	0	4.8529663	0.0225983	0.0364409	0.0407715	0.0078125
1092	1965	Female	242	0	FALSE	0	0.5	0	0		0
1093	1976	Female	52	239	FALSE	0	2.5625	0	0	0	0
1094	1990	Female	668	936	FALSE	0	5.0236816	0.0487175	0.0562583	0.0261841	0.0419922
1095	1978	Female	801	913	FALSE	0	4.1875	0.0390625	0	0	0
1096	1960	Female	679	235	FALSE	0	4.140625	0.078125	0.0664063	0	0.1328125
1097	1975	Female	481	381	FALSE	0	1.25	0	0	0	0
1098	1974	Female	338	939	FALSE	0	3.6112626	0	0.0103287	0	0
1099	1980	Female	803	400	FALSE	0	4.25	0.1875	0	0	0
1100	1974	Female	679	400	FALSE	0	4.765625	0.0859375	0.0664063	0	0.1328125
1101	1978	Female	408	0	FALSE	0	1.25	0	0		0
1102	1975	Male	478	946	FALSE	0	2.1210938	0	0	0	0
1103	1981	Female	501	129	FALSE	0	4.0605469	0	0.015625	0.03125	0
1104	1994	Female	427	520	FALSE	0	3.953125	0.0063477	0.0194092	0	0.0078125
1105	1986	Male	924	944	FALSE	0	5.5477295	0.0438576	0.0784066	0.03125	0.041626
1106	1998	Male	274	498	FALSE	0	5.4930954	0.027024	0.0452797	0.0126953	0.0092254
1107	1974	Female	679	685	FALSE	0	4.96875	0.0722656	0.0664063	0	0.1328125
1108	1974	Male	679	494	FALSE	0	4.9277344	0.0683594	0.0820313	0	0.1328125
1109	1984	Female	477	945	FALSE	0	2.21875	0	0	0	0
1110	1965	Male	501	920	FALSE	0	4.8183594	0.0040283	0.025116	0.0351563	0
1111	1988	Female	512	938	FALSE	0	3.34375	0.0625	0	0	0
1112		Female	512	947	FALSE	0	2.125	0	0	0	0
1113	1980	Female	501	918	FALSE	0	4.8613281	0.0020142	0.0513611	0.0585938	0
1114	1986	Female	814	929	FALSE	0	5.8222656	0.0259399	0.1021109	0.109375	0
1115	1987	Male	644	660	FALSE	0	5.4482422	0.0376587	0.0221815	0.0078125	0.0040283
1116	1988	Female	814	926	FALSE	0	6.0633545	0.0277424	0.0737614	0.041626	0

1117	1989	Female	814	923	FALSE	0	5.8925781	0.0117798	0.0407314	0.0019531	0
1118	1990	Female	814	925	FALSE	0	6.0633545	0.0277424	0.0737614	0.041626	0
1119	1985	Female	950	951	FALSE	0	5.4765625	0.2651367	0.0559739	0.0117188	0.0117188
1120	1986	Male	950	943	FALSE	0	5.1132813	0.0561523	0.059237	0.0625	0.0117188
1121	1981	Female	588	598	FALSE	0	2.375	0	0	0	0
1122	1981	Male	249	952	FALSE	0	4.7524414	0.0300903	0.043509	0.0117188	0.0419922
1123	1982	Female	94	953	FALSE	0	3.65625	0.0234375	0.03125	0	0.0625
1124	1983	Male	597	931	FALSE	0	3.8886719	0.0016479	0.0194702	0	0.0039063
1125	1985	Male	626	933	FALSE	0	5.0182419	0.0286455	0.0244222	0.0161133	0.0093689
1126	1985	Male	249	952	FALSE	0	4.7524414	0.0300903	0.043509	0.0117188	0.0419922
1127	1985	Female	628	954	FALSE	0	2.609375	0	0	0	0
1128		Male	692	520	FALSE	0	3.9785156	0.0097656	0.0194092	0	0.0078125
1129		Female	427	955	FALSE	0	3.6953125	0	0.0194092	0	0.0078125
1130	1989	Female	502	966	FALSE	0	5.875	0.0662231	0.0903215	0.0258789	0.0742188
1131	1991	Male	970	938	FALSE	0	4.4708539	0.0101718	0.0100031	0	0.0015861
1132	1990	Female	732	738	FALSE	0	4.2841797	0.003418	0.0228691	0	0.0351563
1133	1992	Female	735	972	FALSE	0	4.083252	0.0142574	0.0277423	0	0.0083008
1134	1994	Female	213	956	FALSE	0	4.2129494	0.0055612	0.0097038	0	0
1135	1994	Male	706	942	FALSE	0	3.5351563	0	0.0173655	0.015625	0
1136	1990	Female	814	741	FALSE	0	4.9589844	0.0026855	0.0321556	0	0
1137	1976	Female	684	958	FALSE	0	2.21875	0	0	0	0
1138	1988	Female	961	545	FALSE	0	5.8653564	0.2573338	0.0402607	0.0229492	0.0031929
1139	1991	Female	971	918	FALSE	0	5.4974201	0.0021238	0.063423	0.0585938	0.0124893
1140	1992	Male	104	825	FALSE	0	5.659668	0.010376	0.0366538	0.0021973	0.0146484
1141	1959	Male	692	957	FALSE	0	4.2285156	0.0020752	0.0194092	0	0.0078125
1142		Male	732	620	FALSE	0	4.0019531	0.0053101	0.0274271	0	0.0351563
1143	1997	Male	814	825	FALSE	0	5.9995117	0.0083008	0.038457	0.0021973	0
1144	1997	Female	814	959	FALSE	0	6.1030273	0.1296387	0.0361501	0.0014648	0
1145	1997	Female	104	962	FALSE	0	6.2495117	0.0013733	0.0639557	0.0273743	0.0146484
1146	1983	Female	963	965	FALSE	0	3.8828125	0.0195313	0.03125	0	0
1147		Female	740	964	FALSE	0	3.1552539	0	0.0132542	0	0.0018649
1148		Male	735	935	FALSE	0	3.9657593	0.0230269	0.0325531	0	0.0083008
1149		Male	512	980	FALSE	0	3.234375	0	0	0	0
1150		Female	982	916	FALSE	0	3.3276367	0.0004883	0.015625	0	0.03125
1151	1990	Female	981	610	FALSE	0	2.609375	0	0	0	0
1152	1997	Male	909	840	FALSE	0	5.8853293	0.0142442	0.0359066	0.0028568	0.0236816
1153	1992	Female	881	930	FALSE	0	6.743083	0.0064318	0.0918194	0.0427246	0.040494
1154	1993	Female	881	973	FALSE	0	7.050456	0.0026394	0.1196874	0.0301666	0.040494
1155	1994	Male	988	987	FALSE	0	3.5957031	0.005249	0.0107079	0	0
1156	1994	Male	976	871	FALSE	0	1	0	0	0	0
1157	1994	Female	977	764	FALSE	0	5.2581787	0.0120125	0.0207518	0	0.0126953
1158	1995	Male	759	986	FALSE	0	5.2305043	0.0071438	0.0286319	0.001178	0.0229492
1159	1997	Female	104	979	FALSE	0	6.1970215	0.069519	0.0457592	0.0083008	0.0146484
1160	1989	Female	1030	513	FALSE	0	1.5	0	0	0	0
1161	1988	Male	274	1042	FALSE	0	5.2492563	0.002375	0.0302829	0	0.0092254
1162	1994	Female	1002	101	FALSE	0	6.0222168	0.0503082	0.0730212	0.03125	0.0147705
1163	1989	Female	1041	985	FALSE	0	4.9056346	0.0034541	0.0493635	0.0228323	0
1164		Female	1038	783	FALSE	0	4.3046875	0.0009766	0.0362043	0.0429688	0
1165	1989	Female	1041	1050	FALSE	0	3.3984375	0.0039063	0	0	0
1166		Female	572	1059	FALSE	0	4.1484375	0.0039063	0.0039063	0.0078125	0
1167	1997	Female	1040	1006	FALSE	0	6.8251242	0.0057626	0.0640321	0.0017229	0.0139313
1168	1984	Female	1038	917	FALSE	0	4.328125	0	0.0326538	0.0351563	0
1169	1990	Female	1041	1054	FALSE	0	2.7890625	0	0	0	0
1170	1990	Male	669	1048	FALSE	0	2.875	0	0	0	0
1171	1987	Female	983	1061	FALSE	0	3.7581787	0	0.0127609	0	0.012085
1172	1992	Female	1057	968	FALSE	0	5.036377	0.0278015	0.040383	0.0164795	0.0195313

1173	1999	Male	909	999	FALSE	0	6.8243918	0.0165026	0.0749262	0.0064511	0.0236816
1174	1992	Female	1060	967	FALSE	0	3.3747559	0	0.0082968	0	0.0161133
1175	1993	Female	941	997	FALSE	0	4.4711914	0.0081406	0.0259352	0	0.0056152
1176		Female	759	1062	FALSE	0	5.4815961	0.0098812	0.0288012	0.0024917	0.0229492
1177	1993	Male	949	1044	FALSE	0	2.7148438	0	0.0009766	0	0
1178	1978	Female	1055	1056	FALSE	0	1	0	0	0	0
1179		Male	996	1039	FALSE	0	3.520874	0.0046721	0.0135639	0	0
1180		Female	903	1049	FALSE	0	5.056681	0.0039875	0.0272599	0	0.0238037
1181	1993	Female	1047	984	FALSE	0	3.2421875	0.0039063	0	0	0
1182	1965	Male	937	1034	FALSE	0	2.6679688	0	0	0	0
1183	1994	Female	1057	1046	FALSE	0	3.15625	0	0.0174255	0	0.0195313
1184	1994	Female	948	1063	FALSE	0	3.7304688	0.0031738	0.0097046	0	0
1185	1994	Female	974	991	FALSE	0	3.1420898	0	0.0131045	0	0.003418
1186	1972	Male	20	1005	FALSE	0	2.75	0	0	0	0
1187	1994	Male	1065	978	FALSE	0	5.5729147	0.0071462	0.0385012	0.0170898	0
1188	1994	Female	994	1045	FALSE	0	2.887085	0	0.0022899	0	0.0002441
1189	1994	Female	975	1003	FALSE	0	4.6820018	0.0014878	0.0542292	0	0.0228323
1190	1995	Male	992	995	FALSE	0	4.5008545	0.0064468	0.0156617	0.0080566	0
1191	1991	Female	1064	990	FALSE	0	3.2426758	0	0.0048523	0	0
1192		Female	1001	1007	FALSE	0	1	0	0	0	0
1193	1995	Female	1052	969	FALSE	0	4.4941406	0.0107422	0.0109816	0.015625	0.0024414
1194	1997	Female	1058	761	FALSE	0	6.262931	0.0102167	0.0599256	0.0021395	0.0142442
1195	1978	Female	1053	0	FALSE	0	0.5	0	0		0
1196	1996	Male	907	1010	FALSE	0	4.6909082	0.0019587	0.0085802	0	0.0039063
1197	1997	Male	1018	1011	FALSE	0	5.8209667	0.0181582	0.0377541	0.0128174	0.0053627
1198		Female	998	1017	FALSE	0	1	0	0	0	0
1199	1997	Female	1014	940	FALSE	0	4.932251	0.0101776	0.0249397	0.0140991	0
1200	1975	Female	161	1008	FALSE	0	3.1630859	0	0.0155555	0	0.015625
1201	1999	Female	1035	1016	FALSE	0	5.9360024	0.1289673	0.0228048	0.001957	0.0106385
1202	1969	Female	1019	266	FALSE	0	3.3828125	0.0039063	0	0	0
1203	1999	Female	1035	1013	FALSE	0	6.1625813	0.0092552	0.0378043	0.0119133	0.0106385
1204		Male	960	1021	FALSE	0	5.7786576	0.0416072	0.0491871	0.0275269	0.0235091
1205	2000	Male	960	1026	TRUE	0.002050581	6.196929	0.0248988	0.0608366	0.0115198	0.0235091
1206	2001	Male	1027	1025	TRUE	0.001367054	6.1049703	0.0209387	0.0393567	0.0163569	0
1207	1997	Female	1012	0	FALSE	0	2.7313958	0	0.0033034		0.0013251
1208	2002	Female	1023	1000	TRUE	0.001367054	5.3830566	0.0083618	0.024952	0.0078125	0.0005035
1209	2001	Female	1009	1028	TRUE	0.001367054	5.7495728	0.0110569	0.0392611	0.0137844	0.0031738
1210	1994	Female	850	1004	FALSE	0	5.4819336	0.0050049	0.0307496	0.0078125	0.0008545
1211	2002	Male	1029	1022	TRUE	0.001367054	6.1381836	0.03965	0.03856	0.0047607	0.0168304
1212	1985	Female	1024	1015	FALSE	0	1	0	0	0	0
1213	1982	Female	993	189	FALSE	0	4.380396	0.0006437	0.0135871	0	0
1214	1967	Female	0	0	FALSE	0	0	0	0		
1215	1968	Male	0	0	FALSE	0	0	0	0		
1216		Female	0	0	FALSE	0	0	0	0		
1217	1984	Female	1020	1037	FALSE	0	2.6210938	0	0.0029259	0.0039063	0
1218	1964	Male	1036	1033	FALSE	0	1.5	0	0	0	0
1219	1986	Male	427	1043	FALSE	0	4.203125	0.0063477	0.0194092	0	0.0078125
1220		Female	1031	1032	FALSE	0	1	0	0	0	0
1221	1960	Female	989	1051	FALSE	0	2	0	0	0	0
1222	1975	Male	27	1067	FALSE	0	1	0	0	0	0
1223	1970	Female	26	1066	FALSE	0	1	0	0	0	0
1224	1965	Female	71	1068	FALSE	0	2	0	0	0	0
1225	1965	Female	108	1073	FALSE	0	3.9287109	0	0.0337761	0	0.0010376
1226	1960	Female	56	458	FALSE	0	2.25	0.0625	0	0	0
1227	1984	Female	131	1074	FALSE	0	6.0474854	0.0809336	0.096119	0.0148315	0.0673981
1228	1984	Male	100	925	FALSE	0	5.1981201	0.0174866	0.0763958	0.041626	0.0234375

1229	1984	Female	100	784	FALSE	0	5.0351563	0.0009766	0.093235	0.1152344	0.0234375
1230	1984	Female	1079	782	FALSE	0	5.8560791	0.0443811	0.0918114	0.1047974	0.0045166
1231	1983	Female	1078	1072	FALSE	0	5.2176514	0.0581741	0.046892	0.0390625	0.0045166
1232	1988	Female	1078	784	FALSE	0	5.6668701	0.0676155	0.0861109	0.1152344	0.0045166
1233	1987	Female	125	1077	FALSE	0	4.5585938	0.2519531	0.0058594	0	0.0078125
1234	1975	Female	157	1087	FALSE	0	1.9375	0	0	0	0
1235	1987	Female	0	0	FALSE	0	0	0	0		
1236	1990	Female	547	1093	FALSE	0	3.7109375	0.0058594	0.0039063	0	0
1237	1960	Female	801	913	FALSE	0	4.1875	0.0390625	0	0	0
1238	1961	Female	71	354	FALSE	0	3	0	0	0	0
1239	1989	Male	477	378	FALSE	0	3.7654686	0.013258	0.02869	0	0
1240	1985	Male	682	656	FALSE	0	5.1015625	0.0976563	0.0869585	0	0.0996094
1241	1991	Female	427	378	FALSE	0	4.4920311	0.0759504	0.0480991	0	0.0078125
1242	1982	Female	502	1070	FALSE	0	4.9765625	0.0532227	0.0525925	0.015625	0.0742188
1243	1961	Female	514	147	FALSE	0	2.25	0	0	0	0
1244	1977	Female	502	452	FALSE	0	4.4765625	0.0117188	0.04478	0	0.0742188
1245	1960	Female	0	0	FALSE	0	0	0	0		
1246	1979	Female	801	494	FALSE	0	4.4746094	0.0361328	0.015625	0	0
1247	1979	Female	801	685	FALSE	0	4.515625	0.015625	0	0	0
1248	1987	Female	512	1090	FALSE	0	3.7578125	0	0.015625	0.03125	0
1249	1979	Male	501	1069	FALSE	0	4.2480469	0	0.015625	0.03125	0
1250	1981	Female	501	124	FALSE	0	5.0114746	0.0004196	0.0797066	0.0913086	0
1251	1980	Female	501	457	FALSE	0	4.8183594	0.0040283	0.0326538	0.0351563	0
1252	1982	Female	644	925	FALSE	0	5.9354248	0.093668	0.0676936	0.041626	0.0040283
1253	1982	Female	1110	1075	FALSE	0	5.8288574	0.0138741	0.0632506	0.0480957	0.0040283
1254	1985	Female	644	920	FALSE	0	5.5419922	0.0449829	0.0433913	0.0351563	0.0040283
1255	1986	Female	1108	1095	FALSE	0	5.5576172	0.0541992	0.0919228	0.0390625	0.0683594
1256	1983	Female	644	660	FALSE	0	5.4482422	0.0376587	0.0221815	0.0078125	0.0040283
1257	1983	Female	621	1096	FALSE	0	5.4013672	0.0598145	0.1112595	0.078125	0.0380859
1258	1990	Male	427	1109	FALSE	0	4.0546875	0.0126953	0.0194092	0	0.0078125
1259	1991	Female	626	1098	FALSE	0	4.8204552	0.0175974	0.0138775	0	0.0093689
1260	1985	Male	644	1075	FALSE	0	5.8288574	0.0798531	0.0670043	0.0480957	0.0040283
1261	1986	Female	644	1076	FALSE	0	5.9616699	0.0555	0.0902333	0.1047974	0.0040283
1262	1986	Male	814	1113	FALSE	0	5.9677734	0.0099792	0.050979	0.0020142	0
1263	1987	Female	814	929	FALSE	0	5.8222656	0.0259399	0.1021109	0.109375	0
1264	1987	Male	950	1099	FALSE	0	5.3632813	0.1074219	0.121737	0.1875	0.0117188
1265	1983	Male	1108	1107	FALSE	0	5.9482422	0.1831665	0.139328	0.0722656	0.0683594
1266		Male	213	1112	FALSE	0	3.6430374	0	0.0053472	0	0
1267	1984	Female	1071	1107	FALSE	0	5.765625	0.0330811	0.1240044	0.0722656	0.078125
1268	1981	Female	621	1100	FALSE	0	5.7138672	0.0480957	0.1149063	0.0859375	0.0380859
1269	1991	Male	814	1080	FALSE	0	5.9344482	0.0411091	0.0582281	0.0240479	0
1270	1980	Male	1102	1097	FALSE	0	2.6855469	0	0	0	0
1271	1987	Female	819	1086	FALSE	0	4.5615425	0.0220466	0.0374031	0	0.0093689
1272	1992	Female	769	1081	FALSE	0	5.565918	0.0107422	0.0989847	0.046875	0.055603
1273	1991	Female	1122	1127	FALSE	0	4.6809082	0.009491	0.0361451	0	0.0300903
1274	1991	Male	970	520	FALSE	0	4.2599164	0.0032756	0.0100031	0	0.0015861
1275	1993	Female	1125	1085	FALSE	0	6.3806419	0.0129225	0.0689466	0.0430012	0.0286455
1276	1992	Male	735	1123	FALSE	0	4.409668	0.0257568	0.0468653	0.0234375	0.0083008
1277	1993	Female	1128	1121	FALSE	0	4.1767578	0	0.0144926	0	0.0097656
1278	2001	Male	740	1094	TRUE	0.001367054	5.6670947	0.0132758	0.0643717	0.0487175	0.0018649
1279	1991	Female	971	1084	FALSE	0	5.8503864	0.0025846	0.0754993	0.0422974	0.0124893
1280	1992	Male	1126	1091	FALSE	0	5.8027039	0.0386951	0.0652529	0.0225983	0.0300903
1281	1994	Female	1115	1116	FALSE	0	6.7557983	0.0431027	0.0792312	0.0277424	0.0376587
1282	1997	Female	104	1114	FALSE	0	6.1083984	0.0648499	0.0852409	0.0259399	0.0146484
1283	1997	Female	1105	1119	FALSE	0	6.512146	0.0296698	0.2125477	0.2651367	0.0438576
1284	1990	Female	971	140	FALSE	0	5.6263264	0.0195824	0.0749891	0	0.0124893

1285	1987	Female	1122	1088	FALSE	0	4.2355957	0.0131226	0.0361451	0	0.0300903
1286	1989	Male	971	660	FALSE	0	5.3607014	0.001301	0.0159681	0.0078125	0.0124893
1287	1995	Male	909	840	FALSE	0	5.8853293	0.0142442	0.0359066	0.0028568	0.0236816
1288		Female	909	765	FALSE	0	5.1181641	0.013916	0.0299192	0.0007324	0.0236816
1289		Female	759	1147	FALSE	0	4.8627832	0.0036112	0.0317692	0	0.0229492
1290	1986	Female	644	877	FALSE	0	4.8701172	0.003418	0.0182753	0	0.0040283
1291	1999	Male	909	840	FALSE	0	5.8853293	0.0142442	0.0359066	0.0028568	0.0236816
1292	1996	Female	834	878	FALSE	0	4.5322266	0.0083008	0.0136108	0	0.0078125
1293	2001	Female	1143	1145	TRUE	0.001367054	7.1245117	0.0870342	0.0558399	0.0013733	0.0083008
1294	2003	Male	1140	1136	TRUE	0.001367054	6.3093262	0.038681	0.0407021	0.0026855	0.010376
1295	1997	Female	1131	1133	FALSE	0	5.2770529	0.0153645	0.0308387	0.0142574	0.0101718
1296	2001	Female	960	908	TRUE	0.001367054	6.0389065	0.0295526	0.0742801	0.0228323	0.0235091
1297	1996	Male	850	1118	FALSE	0	6.4472046	0.0266709	0.0631887	0.0277424	0.0008545
1298	1997	Female	1140	1117	FALSE	0	6.776123	0.0715218	0.0493404	0.0117798	0.010376
1299	1992	Female	909	1146	FALSE	0	5.3417969	0.0148315	0.0541505	0.0195313	0.0236816
1300	1986	Female	628	1137	FALSE	0	2.859375	0	0	0	0
1301	1989	Male	899	546	FALSE	0	5.459684	0.0035027	0.0330287	0.0234375	0.0055404
1302	1989	Male	899	918	FALSE	0	5.428434	0.0037593	0.0631343	0.0585938	0.0055404
1303	1999	Female	909	1103	FALSE	0	5.4306641	0.0005951	0.0368775	0	0.0236816
1304	1995	Male	909	1000	FALSE	0	5.4316406	0.0096436	0.0329713	0.0078125	0.0236816
1305	1988	Female	0	0	FALSE	0	0	0	0		
1306	1960	Female	0	0	FALSE	0	0	0	0		
1307	1990	Female	1057	1101	FALSE	0	3.53125	0	0.0174255	0	0.0195313
1308	2001	Female	1023	1154	TRUE	0.001367054	6.8770346	0.0064511	0.0820512	0.0026394	0.0005035
1309	2003	Female	1035	1130	TRUE	0.001367054	6.5342854	0.0007337	0.0892835	0.0662231	0.0106385
1310	1995	Female	1149	1151	FALSE	0	3.921875	0.03125	0	0	0
1311	1998	Female	1058	785	FALSE	0	6.8141856	0.0117726	0.0675822	0.0430012	0.0142442
1312		Female	841	1150	FALSE	0	4.5964355	0.0124207	0.0197126	0.0004883	0.0175781
1313	2001	Male	1040	1144	TRUE	0.001367054	6.366333	0.006644	0.0942784	0.1296387	0.0139313
1314	2001	Male	1040	1159	TRUE	0.001367054	6.4133301	0.0082101	0.0697758	0.069519	0.0139313
1315	2003	Male	1040	1114	TRUE	0.001367054	6.2259521	0.0014963	0.0764283	0.0259399	0.0139313
1316	1998	Female	1155	1134	FALSE	0	4.9043263	0.0015195	0.0155559	0.0055612	0.005249
1317	1999	Male	251	1157	FALSE	0	5.9075604	0.016344	0.0291785	0.0120125	0.0036439
1318	2000	Male	1027	761	TRUE	0.001367054	5.6094204	0.0039026	0.0436584	0.0021395	0
1319	2000	Male	251	1022	TRUE	0.001367054	5.6339398	0.0149617	0.0211025	0.0047607	0.0036439
1320	1999	Female	0	0	FALSE	0	0	0	0		
1321		Female	1158	1129	FALSE	0	5.4629084	0.0124765	0.0274902	0	0.0071438
1322	1990	Female	788	1059	FALSE	0	4.7299805	0.0099487	0.0196153	0.0078125	0.0161133
1323	2000	Male	1040	1139	TRUE	0.001367054	6.0635294	0.0037206	0.0464333	0.0021238	0.0139313
1324	2002	Female	1040	1084	TRUE	0.001367054	5.8435669	0.0005069	0.0771647	0.0422974	0.0139313
1325	2002	Female	1040	1083	TRUE	0.001367054	5.8435669	0.0005069	0.0771647	0.0422974	0.0139313
1326	1987	Female	864	1212	FALSE	0	2.25	0	0	0	0
1327	2005	Female	1173	1138	TRUE	0.001367054	7.3448741	0.0334404	0.1887131	0.2573338	0.0165026
1328	2008	Male	1205	1208	TRUE	0.001367054	6.7899928	0.0135524	0.0586629	0.0083618	0.0248988
1329	1970	Female	1215	1214	FALSE	0	1	0	0	0	0
1330	2006	Male	1173	1138	TRUE	0.001367054	7.3448741	0.0334404	0.1887131	0.2573338	0.0165026
1331	1986	Male	1141	1213	FALSE	0	5.3044558	0.0122211	0.0178331	0.0006437	0.0020752
1332	1991	Female	1124	1217	FALSE	0	4.2548828	0.0024109	0.012006	0	0.0016479
1333	1998	Female	909	1166	FALSE	0	5.4746094	0.0127563	0.0329637	0.0039063	0.0236816
1334		Female	1148	1220	FALSE	0	3.4828796	0	0.0274152	0	0.0230269
1335	2000	Male	1082	1162	TRUE	0.001367054	6.5457153	0.038833	0.0980118	0.0503082	0.01651
1336	1993	Female	1219	1111	FALSE	0	4.7734375	0.0083008	0.0440668	0.0625	0.0063477
1337	2001	Female	1152	1167	TRUE	0.001367054	7.3552268	0.0402919	0.0595325	0.0057626	0.0142442
1338	1994	Male	1060	1169	FALSE	0	4.2692871	0.0176086	0.0082968	0	0.0161133
1339	1994	Male	759	1165	FALSE	0	4.984375	0.0074463	0.0270952	0.0039063	0.0229492
1340	1995	Female	740	1171	FALSE	0	5.0343432	0.0039301	0.0196347	0	0.0018649

1341	1995	Female	983	1163	FALSE	0	5.710996	0.0100372	0.0390845	0.0034541	0.012085
1342	1995	Female	1089	1160	FALSE	0	3.9599609	0	0.0511091	0	0.0670395
1343	1996	Female	1170	1172	FALSE	0	4.9556885	0.0168152	0.0335309	0.0278015	0
1344	2003	Male	1197	1153	TRUE	0.001367054	7.2820248	0.0158104	0.0764437	0.0064318	0.0181582
1345	2003	Female	814	1210	TRUE	0.001367054	6.2780762	0.005188	0.0421434	0.0050049	0
1346	1997	Female	1018	1171	FALSE	0	5.3476391	0.0169337	0.0280106	0	0.0053627
1347	1999	Female	1187	1176	FALSE	0	6.5272554	0.0098865	0.0418851	0.0098812	0.0071462
1348	1997	Female	1219	1132	FALSE	0	5.2436523	0.010376	0.0259213	0.003418	0.0063477
1349	1997	Male	907	1188	FALSE	0	4.3068237	0.0322723	0.0030981	0	0.0039063
1350	1976	Female	786	1178	FALSE	0	2.71875	0	0	0	0
1351	1976	Male	0	0	FALSE	0	0	0	0		
1352		Female	740	1181	FALSE	0	4.7763476	0.0042858	0.0152074	0.0039063	0.0018649
1353		Male	1177	1200	FALSE	0	3.9389648	0.0025787	0.008266	0	0
1354	1998	Male	1135	1191	FALSE	0	4.388916	0.0322151	0.0111089	0	0
1355	1998	Male	907	1180	FALSE	0	5.3916217	0.0057529	0.0175225	0.0039875	0.0039063
1356	1999	Female	1179	1189	FALSE	0	5.1014379	0.0031273	0.0369044	0.0014878	0.0046721
1357	1999	Female	1057	1184	FALSE	0	4.7714844	0.0175781	0.0238493	0.0031738	0.0195313
1358	2001	Female	1027	1194	TRUE	0.001367054	6.4206195	0.0119151	0.0433175	0.0102167	0
1359	2000	Female	983	1174	TRUE	0.001367054	4.9455566	0.0090494	0.0169094	0	0.012085
1360	2001	Male	1190	1022	TRUE	0.001367054	5.605896	0.0511646	0.0191853	0.0047607	0.0064468
1361		Female	1156	1198	FALSE	0	2	0	0	0	0
1362	2001	Male	1161	1199	TRUE	0.001367054	6.0907537	0.0277774	0.0337247	0.0101776	0.002375
1363	1957	Female	0	0	FALSE	0	0	0	0		
1364	2001	Male	201	1195	TRUE	0.001367054	1.75	0	0	0	0
1365	2001	Female	907	1175	TRUE	0.001367054	5.098877	0.0058594	0.0188855	0.0081406	0.0039063
1366	1982	Female	1182	1221	FALSE	0	3.3339844	0.0625	0	0	0
1367	2001	Male	267	1185	TRUE	0.001367054	5.0004703	0.0020898	0.0452269	0	0.0643881
1368	2001	Female	1196	1104	TRUE	0.001367054	5.3220166	0.0056106	0.0180779	0.0063477	0.0019587
1369	2003	Male	1023	1203	TRUE	0.001367054	6.4330973	0.0100948	0.0444006	0.0092552	0.0005035
1370	1994	Female	850	1168	FALSE	0	5.5795898	0.0241699	0.0297869	0	0.0008545
1371		Male	1204	1192	FALSE	0	4.3893288	0	0.0443739	0	0.0416072
1372	2004	Male	1158	1183	TRUE	0.001367054	5.1933772	0.0115	0.0264984	0	0.0071438
1373	1996	Female	814	1168	FALSE	0	5.7011719	0.0170898	0.04067	0	0
1374	2005	Female	1206	1201	TRUE	0.001367054	7.0204863	0.0093955	0.1041512	0.1289673	0.0209387
1375	1992	Female	1120	1164	FALSE	0	5.7089844	0.0175781	0.0746043	0.0009766	0.0561523
1376	1982	Female	666	1207	FALSE	0	3.7597409	0.0034966	0.0094295	0	0
1377	1982	Male	1186	1202	FALSE	0	4.0664063	0.0234375	0.0019531	0.0039063	0
1378	1965	Female	1218	1092	FALSE	0	2	0	0	0	0
1379	2005	Male	1106	1193	TRUE	0.001367054	5.993618	0.0171319	0.0463429	0.0107422	0.027024
1380	2007	Female	1211	1209	TRUE	0.001367054	6.9438782	0.0406383	0.0632825	0.0110569	0.03965
1381	1978	Female	0	0	FALSE	0	0	0	0		
1382		Female	1142	1216	FALSE	0	3.0009766	0	0.0162958	0	0.0053101
1383	1973	Female	102	1223	FALSE	0	2	0	0	0	0
1384	1980	Female	655	1224	FALSE	0	3.765625	0.0039063	0.015625	0	0
1385	1964	Female	108	1226	FALSE	0	5.0537109	0.0185547	0.0650261	0.0625	0.0010376
1386	1968	Female	117	1225	FALSE	0	4.0893555	0.0092773	0.0481381	0	0.0625
1387	1973	Female	132	658	FALSE	0	4.8393555	0.0480957	0.0518564	0.0400391	0.03125
1388	1978	Male	131	129	FALSE	0	5.0574951	0.0422974	0.0883128	0.03125	0.0673981
1389	1982	Female	1078	541	FALSE	0	4.5067139	0.0022125	0.0198534	0	0.0045166
1390	1986	Male	133	920	FALSE	0	5.6615601	0.0595665	0.0885533	0.0351563	0.0422974
1391	1975	Female	1222	1234	FALSE	0	2.46875	0	0	0	0
1392	1994	Female	251	1235	FALSE	0	3.2784711	0	0.012921	0	0.0036439
1393	1959	Female	0	0	FALSE	0	0	0	0		
1394		Male	251	1236	FALSE	0	5.1339398	0.0092597	0.0177924	0.0058594	0.0036439
1395	1959	Female	649	279	FALSE	0	2.125	0	0	0	0
1396	1981	Female	801	91	FALSE	0	4.375	0.0175781	0.0078125	0.015625	0

1397	1988	Female	1071	1095	FALSE	0	5.375	0.0639648	0.0765991	0.0390625	0.078125
1398	1969	Male	83	1238	FALSE	0	3.90625	0	0	0	0
1399	1990	Male	1228	685	FALSE	0	5.5521851	0.0365677	0.0462732	0	0.0174866
1400	1983	Male	1071	685	FALSE	0	5.234375	0.0234375	0.0570679	0	0.078125
1401	1988	Male	502	1251	FALSE	0	5.7607422	0.0170898	0.0630553	0.0040283	0.0742188
1402	1990	Female	1228	1246	FALSE	0	5.8363647	0.003417	0.0718699	0.0361328	0.0174866
1403	1985	Female	1071	1250	FALSE	0	5.7869873	0.0110779	0.0971143	0.0004196	0.078125
1404	1983	Female	950	1244	FALSE	0	5.4765625	0.2651367	0.0559739	0.0117188	0.0117188
1405	1984	Female	950	1244	FALSE	0	5.4765625	0.2651367	0.0559739	0.0117188	0.0117188
1406	1982	Female	621	1245	FALSE	0	3.3310547	0	0.0415878	0	0.0380859
1407	1991	Male	814	1229	FALSE	0	6.0546875	0.0150452	0.0714034	0.0009766	0
1408	1986	Female	1108	1237	FALSE	0	5.5576172	0.0541992	0.0919228	0.0390625	0.0683594
1409	1991	Female	1240	815	FALSE	0	6.2460938	0.1049194	0.1268042	0.0302734	0.0976563
1410	1956	Female	0	0	FALSE	0	0	0	0		
1411	1982	Female	127	1243	FALSE	0	3.5625	0	0.03125	0	0.0625
1412	1997	Female	814	1229	FALSE	0	6.0546875	0.0150452	0.0714034	0.0009766	0
1413	1981	Female	801	822	FALSE	0	5.046875	0.0258789	0.0669365	0.0722656	0
1414	1998	Male	814	1229	FALSE	0	6.0546875	0.0150452	0.0714034	0.0009766	0
1415		Male	0	0	FALSE	0	0	0	0		
1416	1984	Female	1071	1246	FALSE	0	5.5185547	0.0345459	0.0826645	0.0361328	0.078125
1417	1990	Female	769	761	FALSE	0	6.1049343	0.0009984	0.0957606	0.0021395	0.055603
1418	1988	Female	792	750	FALSE	0	5.1437988	0.0158615	0.0421361	0.0039063	0.0283203
1419	1990	Male	735	743	FALSE	0	4.112793	0.0238037	0.0198878	0	0.0083008
1420	1991	Female	769	928	FALSE	0	6.0882568	0.0342388	0.080508	0.0045166	0.055603
1421	1990	Female	1262	1255	FALSE	0	6.7626953	0.0308189	0.1007947	0.0541992	0.0099792
1422	1991	Female	971	1233	FALSE	0	5.6009358	0.0009765	0.14023	0.2519531	0.0124893
1423	1988	Male	1249	1267	FALSE	0	6.0068359	0.0371399	0.0843041	0.0330811	0
1424	1992	Female	769	761	FALSE	0	6.1049343	0.0009984	0.0957606	0.0021395	0.055603
1425	1992	Female	739	1248	FALSE	0	4.0664063	0	0.0078125	0	0
1426	1987	Female	814	1253	FALSE	0	6.4515381	0.0226765	0.0624666	0.0138741	0
1427	1970	Male	756	651	FALSE	0	3.6826172	0.0058594	0.0243267	0	0.0410156
1428	1988	Female	814	1253	FALSE	0	6.4515381	0.0226765	0.0624666	0.0138741	0
1429	1994	Female	1239	1271	FALSE	0	5.1635056	0.0471994	0.0500963	0.0220466	0.013258
1430	1995	Male	740	1123	FALSE	0	4.9833789	0.0111939	0.0402318	0.0234375	0.0018649
1431	1990	Male	1262	1268	FALSE	0	6.8408203	0.043026	0.1089625	0.0480957	0.0099792
1432	1989	Female	1260	920	FALSE	0	6.0472412	0.0361557	0.0958694	0.0351563	0.0798531
1433	1989	Female	1265	1242	FALSE	0	6.4624023	0.0628357	0.1999951	0.0532227	0.1831665
1434	1989	Female	1265	1257	FALSE	0	6.6748047	0.1437531	0.2306966	0.0598145	0.1831665
1435	1998	Male	1258	1259	FALSE	0	5.4375714	0.0152156	0.0315444	0.0175974	0.0126953
1436	1992	Male	1228	1257	FALSE	0	6.2997437	0.0120339	0.1284827	0.0598145	0.0174866
1437	1991	Female	1115	103	FALSE	0	6.3842773	0.0578156	0.0735463	0.0166016	0.0376587
1438	1999	Female	740	972	FALSE	0	4.6569629	0.0061188	0.0211088	0	0.0018649
1439	1999	Female	1266	311	FALSE	0	5.1012695	0.0026801	0.0222968	0.0061955	0
1440	1991	Male	971	1231	FALSE	0	5.9304646	0.0024304	0.063231	0.0581741	0.0124893
1441	1992	Female	104	1254	FALSE	0	5.9682617	0.0018921	0.0657511	0.0449829	0.0146484
1442	1992	Female	104	1252	FALSE	0	6.164978	0.0172596	0.1000503	0.093668	0.0146484
1443	1995	Female	814	1253	FALSE	0	6.4515381	0.0226765	0.0624666	0.0138741	0
1444	1998	Female	104	962	FALSE	0	6.2495117	0.0013733	0.0639557	0.0273743	0.0146484
1445	1998	Female	1264	1091	FALSE	0	6.1081238	0.0001297	0.1371487	0.0225983	0.1074219
1446	1985	Female	1270	716	FALSE	0	3.5927734	0	0	0	0
1447	1984	Female	1071	966	FALSE	0	5.8046875	0.0394897	0.1026094	0.0258789	0.078125
1448	1994	Female	1125	1232	FALSE	0	6.342556	0.0044745	0.1001361	0.0676155	0.0286455
1449	1991	Female	1126	1227	FALSE	0	6.3999634	0.0055017	0.1207817	0.0809336	0.0300903
1450	1989	Female	732	1146	FALSE	0	4.703125	0.0018311	0.044548	0.0195313	0.0351563
1451	1992	Female	881	1085	FALSE	0	6.8480024	0.0064511	0.0858248	0.0430012	0.040494
1452	1994	Male	1131	1285	FALSE	0	5.3532248	0.0142091	0.0344332	0.0131226	0.0101718

1453		Female	1143	1282	FALSE	0	7.0539551	0.10779	0.0955007	0.0648499	0.0083008
1454	2001	Male	1143	1229	TRUE	0.001367054	6.517334	0.0158577	0.0702795	0.0009766	0.0083008
1455	1997	Male	841	1241	FALSE	0	5.1786327	0.0163219	0.0718579	0.0759504	0.0175781
1456	1997	Female	427	1277	FALSE	0	5.0336914	0.0088196	0.0266555	0	0.0078125
1457	1999	Female	1286	1284	FALSE	0	6.4935139	0.1352	0.0551757	0.0195824	0.001301
1458	2000	Female	1286	1284	TRUE	0.001367054	6.4935139	0.1352	0.0551757	0.0195824	0.001301
1459	1991	Male	909	1256	FALSE	0	6.1245117	0.0016708	0.0585675	0.0376587	0.0236816
1460	1985	Male	1141	0	FALSE	0	2.6142578	0	0.010722		0.0020752
1461	1996	Female	1280	1230	FALSE	0	6.8293915	0.0052921	0.1167704	0.0443811	0.0386951
1462	1998	Female	1140	1118	FALSE	0	6.8615112	0.0544951	0.0730534	0.0277424	0.010376
1463	2007	Female	1143	1282	TRUE	0.001367054	7.0539551	0.10779	0.0955007	0.0648499	0.0083008
1464	1998	Female	104	1281	FALSE	0	6.5751648	0.0413141	0.0819993	0.0431027	0.0146484
1465	1984	Female	1038	661	FALSE	0	4.5379639	0.0007019	0.0198534	0.0045166	0
1466	1993	Female	1301	1284	FALSE	0	6.5430052	0.0515657	0.0647594	0.0195824	0.0035027
1467	2003	Male	1269	1299	TRUE	0.001367054	6.6381226	0.0039563	0.0825612	0.0148315	0.0411091
1468	1993	Male	427	1300	FALSE	0	4.375	0.0063477	0.0194092	0	0.0078125
1469	1996	Male	1302	140	FALSE	0	6.0189045	0.0632836	0.0962554	0	0.0037593
1470	1996	Female	983	1010	FALSE	0	5.0858057	0.0039301	0.0193881	0	0.012085
1471	1998	Female	1058	1275	FALSE	0	7.1329856	0.014887	0.0653087	0.0129225	0.0142442
1472	1996	Female	1274	1039	FALSE	0	4.5193625	0.0053463	0.0127046	0	0.0032756
1473	2002	Female	1040	1229	TRUE	0.001367054	5.8323975	0.0063114	0.0607875	0.0009766	0.0139313
1474	1997	Female	994	1273	FALSE	0	5.2275391	0.0044717	0.0249364	0.009491	0.0002441
1475	2001	Female	1287	1263	TRUE	0.001367054	6.8537975	0.0022264	0.0875207	0.0259399	0.0142442
1476	1960	Female	0	0	FALSE	0	0	0	0		
1477	2002	Female	1023	1025	TRUE	0.001367054	6.1676228	0.012702	0.05185	0.0163569	0.0005035
1478	1997	Female	814	1290	FALSE	0	5.972168	0.0053711	0.0351585	0.003418	0
1479	2005	Female	960	1028	TRUE	0.001367054	5.8769852	0.0158813	0.052917	0.0137844	0.0235091
1480	1993	Male	909	1059	FALSE	0	5.1269531	0.0072021	0.0329713	0.0078125	0.0236816
1481	2002	Male	1040	1283	TRUE	0.001367054	6.5708923	0.000665	0.1316829	0.0296698	0.0139313
1482	1994	Female	909	1059	FALSE	0	5.1269531	0.0072021	0.0329713	0.0078125	0.0236816
1483	2001	Female	0	0	TRUE	0.001367054	0	0	0		
1484	2003	Female	1040	1084	TRUE	0.001367054	5.8435669	0.0005069	0.0771647	0.0422974	0.0139313
1485	2005	Female	1291	1308	TRUE	0.001367054	7.381182	0.0104691	0.0688061	0.0064511	0.0142442
1486	2009	Female	1205	1320	TRUE	0.001367054	4.0984645	0	0.0421103	0	0.0248988
1487	2008	Female	1313	1298	TRUE	0.001367054	7.571228	0.0695984	0.1088146	0.0715218	0.006644
1488	2007	Female	1294	1320	TRUE	0.001367054	4.1546631	0	0.0389044	0	0.038681
1489	1960	Female	1182	1306	FALSE	0	2.3339844	0	0	0	0
1490	2008	Female	1205	1308	TRUE	0.001367054	7.5369818	0.0141886	0.0860968	0.0064511	0.0248988
1491	1999	Male	1082	1162	FALSE	0	6.5457153	0.038833	0.0980118	0.0503082	0.01651
1492	2002	Male	1197	1153	TRUE	0.001367054	7.2820248	0.0158104	0.0764437	0.0064318	0.0181582
1493	2002	Female	1197	840	TRUE	0.001367054	6.395422	0.0079816	0.0344549	0.0028568	0.0181582
1494	1998	Male	1187	1154	FALSE	0	7.3116854	0.0082214	0.0836916	0.0026394	0.0071462
1495	2005	Female	1314	1293	TRUE	0.001367054	7.7689209	0.0872439	0.1077136	0.0870342	0.0082101
1496	2003	Male	1143	1210	TRUE	0.001367054	6.7407227	0.037426	0.0410196	0.0050049	0.0083008
1497	1997	Male	251	1307	FALSE	0	5.0440961	0.0103025	0.0216338	0	0.0036439
1498	2003	Female	1197	1194	TRUE	0.001367054	7.0419488	0.0117696	0.0623784	0.0102167	0.0181582
1499	1998	Male	1135	1305	FALSE	0	2.7675781	0	0.0086827	0	0
1500	2006	Female	1304	1166	TRUE	0.001367054	5.7900391	0.0099182	0.0250471	0.0039063	0.0096436
1501	2006	Female	1318	1325	TRUE	0.001367054	6.7264936	0.008806	0.0625116	0.0005069	0.0039026
1502	2009	Male	1323	1309	TRUE	0.001367054	7.2989074	0.0134878	0.0699664	0.0007337	0.0037206
1503	2002	Male	1027	1311	TRUE	0.001367054	6.6962469	0.0133812	0.0478321	0.0117726	0
1504	2003	Male	1012	1310	TRUE	0.001367054	5.1923333	0.0016036	0.0189284	0.03125	0.0013251
1505		Male	1190	1289	FALSE	0	5.6818188	0.0078658	0.0286366	0.0036112	0.0064468
1506	2004	Male	1190	1312	TRUE	0.001367054	5.548645	0.0097089	0.026948	0.0124207	0.0064468
1507	2005	Male	1278	1316	TRUE	0.001367054	6.2857105	0.0065747	0.0469224	0.0015195	0.0132758
1508	2012	Male	1205	1324	TRUE	0.001367054	7.0202479	0.0120457	0.0809266	0.0005069	0.0248988

1509		Female	1317	1321	FALSE	0	6.6852344	0.0307782	0.0423347	0.0124765	0.016344
1510	1998	Male	1187	1084	FALSE	0	6.3152049	0.0191974	0.0861235	0.0422974	0.0071462
1511	1994	Male	909	1322	FALSE	0	5.7653809	0.0198135	0.0437495	0.0099487	0.0236816
1512	2007	Female	1197	1337	TRUE	0.001367054	7.5880967	0.0164408	0.0763262	0.0402919	0.0181582
1513	1990	Female	769	1366	FALSE	0	5.4516602	0.0009766	0.0919046	0.0625	0.055603
1514	1992	Male	1331	1261	FALSE	0	6.6330629	0.0036822	0.0852808	0.0555	0.0122211
1515	1989	Male	1377	1247	FALSE	0	5.2910156	0.0023193	0.0204849	0.015625	0.0234375
1516	2008	Female	1318	1333	TRUE	0.001367054	6.5420149	0.0142757	0.0463451	0.0127563	0.0039026
1517	1991	Female	769	1366	FALSE	0	5.4516602	0.0009766	0.0919046	0.0625	0.055603
1518	1989	Female	834	1376	FALSE	0	4.6767454	0.0088584	0.0103528	0.0034966	0.0078125
1519	2006	Female	1335	1272	TRUE	0.001367054	7.0558167	0.0399272	0.1208512	0.0107422	0.038833
1520	1990	Male	1364	1381	FALSE	0	1.875	0	0	0	0
1521	2011	Female	1362	1380	TRUE	0.001367054	7.5173159	0.0160119	0.0809572	0.0406383	0.0277774
1522	1993	Female	1125	1366	FALSE	0	5.1761131	0.0008886	0.0574341	0.0625	0.0286455
1523	2009	Male	1369	1288	TRUE	0.001367054	6.7756307	0.0428074	0.048733	0.013916	0.0100948
1524	1994	Female	1125	1366	FALSE	0	5.1761131	0.0008886	0.0574341	0.0625	0.0286455
1525	2009	Female	1344	1279	TRUE	0.001367054	7.5662056	0.0252831	0.0844671	0.0025846	0.0158104
1526	2009	Female	1205	1374	TRUE	0.001367054	7.6087077	0.0124522	0.0983944	0.0093955	0.0248988
1527	2010	Female	1330	1279	TRUE	0.001367054	7.5976303	0.0196607	0.1468658	0.0025846	0.0334404
1528	2009	Female	1330	1303	TRUE	0.001367054	7.3877691	0.081022	0.1266468	0.0005951	0.0334404
1529	2004	Female	1286	1375	TRUE	0.001367054	6.5348429	0.0237257	0.0540597	0.0175781	0.001301
1530		Female	1148	1382	FALSE	0	4.4833679	0.0082753	0.0355631	0	0.0230269
1531	2010	Female	1379	1358	TRUE	0.001367054	7.2071188	0.0107064	0.0586986	0.0119151	0.0171319
1532	1996	Female	1125	1366	FALSE	0	5.1761131	0.0008886	0.0574341	0.0625	0.0286455
1533	2011	Male	1328	1327	TRUE	0.001367054	8.0674335	0.0090883	0.1436316	0.0334404	0.0135524
1534		Female	427	1334	FALSE	0	4.6867523	0.0392628	0.0331168	0	0.0078125
1535	1996	Male	1170	1326	FALSE	0	3.5625	0	0	0	0
1536	2004	Female	1297	1373	TRUE	0.001367054	7.0741882	0.0863297	0.0726196	0.0170898	0.0266709
1537	2007	Male	1315	1345	TRUE	0.001367054	7.2520142	0.0734531	0.0624615	0.005188	0.0014963
1538	2001	Male	1040	1373	TRUE	0.001367054	6.1654053	0.0027952	0.0422597	0.0170898	0.0139313
1539	2002	Female	1040	1370	TRUE	0.001367054	6.1046143	0.0106437	0.0403457	0.0241699	0.0139313
1540	1970	Female	473	1329	FALSE	0	2.90625	0	0	0	0
1541	2004	Male	1291	1347	TRUE	0.001367054	7.2062924	0.0292342	0.0504983	0.0098865	0.0142442
1542	1998	Male	1276	1332	FALSE	0	5.3322754	0.0136385	0.0429015	0.0024109	0.0257568
1543	2001	Female	1023	1340	TRUE	0.001367054	5.8689783	0.0134497	0.0327896	0.0039301	0.0005035
1544	2008	Male	1291	1336	TRUE	0.001367054	6.3293834	0.0222797	0.0508205	0.0083008	0.0142442
1545	2001	Female	1349	1346	TRUE	0.001367054	5.8272314	0.0068806	0.0398702	0.0169337	0.0322723
1546	2002	Female	1339	1292	TRUE	0.001367054	5.7583008	0.0081406	0.0280692	0.0083008	0.0074463
1547	2003	Male	1027	1347	TRUE	0.001367054	6.5527818	0.0355812	0.0342312	0.0098865	0
1548	2003	Male	1319	1348	TRUE	0.001367054	6.4387961	0.0267128	0.0358883	0.010376	0.0149617
1549	2003	Female	1354	1295	TRUE	0.001367054	5.8329845	0.009571	0.0443478	0.0153645	0.0322151
1550	2003	Female	1190	1359	TRUE	0.001367054	5.7232056	0.016222	0.0239067	0.0090494	0.0064468
1551	2004	Female	1204	1352	TRUE	0.001367054	6.2775026	0.0161836	0.0540879	0.0042858	0.0416072
1552	2005	Female	1367	1342	TRUE	0.001367054	5.4802156	0.0033352	0.0491656	0	0.0020898
1553	2005	Female	1354	1343	TRUE	0.001367054	5.6723022	0.0060093	0.0463742	0.0168152	0.0322151
1554	2005	Female	1205	1341	TRUE	0.001367054	6.9539625	0.0336794	0.066475	0.0100372	0.0248988
1555	1988	Male	1377	1213	FALSE	0	5.2234011	0.0074227	0.0197835	0.0006437	0.0234375
1556	2005	Female	1355	1296	TRUE	0.001367054	6.7152641	0.0150617	0.0624061	0.0295526	0.0057529
1557	2006	Female	1360	1357	TRUE	0.001367054	6.1886902	0.0337634	0.0551883	0.0175781	0.0511646
1558	2006	Male	1371	1361	TRUE	0.001367054	4.1946644	0	0.022187	0	0
1559	2006	Female	1353	1368	TRUE	0.001367054	5.6304907	0.0073708	0.0172053	0.0056106	0.0025787
1560	2007	Female	1372	1356	TRUE	0.001367054	6.1474075	0.0062427	0.038805	0.0031273	0.0115
1561	2007	Male	1338	1365	TRUE	0.001367054	5.684082	0.0138588	0.0251968	0.0058594	0.0176086
1562	1985	Female	616	1350	FALSE	0	4.003418	0.0161133	0.0077778	0	0
1563	1985	Female	1351	1363	FALSE	0	1	0	0	0	0
1564	1961	Female	649	1378	FALSE	0	3.125	0	0	0	0

1565	1980	Male	27	0	FALSE	0	0.5	0	0		0
1566	1968	Female	0	0	FALSE	0	0	0	0		
1567	1970	Female	92	1383	FALSE	0	3.578125	0.03125	0	0	0
1568	1968	Female	0	0	FALSE	0	0	0	0		
1569	1981	Male	132	0	FALSE	0	2	0	0.015625		0.03125
1570	1985	Female	1071	91	FALSE	0	5.09375	0.2929688	0.0648804	0.015625	0.078125
1571	1980	Female	132	656	FALSE	0	4.1875	0.0351563	0.03125	0	0.03125
1572	1981	Female	132	110	FALSE	0	3.25	0.03125	0.015625	0	0.03125
1573	1983	Female	99	1387	FALSE	0	5.7009277	0.0523071	0.1057969	0.0480957	0.078125
1574	1972	Female	117	1385	FALSE	0	4.6518555	0.1030273	0.0724371	0.0185547	0.0625
1575	1987	Female	125	1387	FALSE	0	5.4587402	0.0351715	0.0526353	0.0480957	0.0078125
1576	1988	Female	125	1387	FALSE	0	5.4587402	0.0351715	0.0526353	0.0480957	0.0078125
1577	1981	Female	132	1076	FALSE	0	5.0524902	0.041626	0.087583	0.1047974	0.03125
1578	1984	Female	1078	1384	FALSE	0	5.1864014	0.0444565	0.0295885	0.0039063	0.0045166
1579	1987	Male	125	546	FALSE	0	5.2460938	0.2636719	0.0251617	0.0234375	0.0078125
1580	1987	Female	662	777	FALSE	0	5.5332031	0.0427246	0.0572351	0.0351563	0.0009766
1581	1971	Female	156	152	FALSE	0	2.21875	0	0.015625	0	0
1582	1985	Female	547	1391	FALSE	0	3.6640625	0.0029297	0.0039063	0	0
1583	1985	Female	322	1393	FALSE	0	1.75	0	0	0	0
1584	1983	Female	351	1385	FALSE	0	5.8061523	0.0213623	0.0729254	0.0185547	0.0634766
1585	1982	Female	351	325	FALSE	0	4.3183594	0.0030518	0.0317383	0	0.0634766
1586	1970	Female	0	0	FALSE	0	0	0	0		
1587	1970	Female	0	0	FALSE	0	0	0	0		
1588	1968	Male	0	0	FALSE	0	0	0	0		
1589	1975	Female	1398	1395	FALSE	0	4.015625	0.015625	0	0	0
1590	1985	Female	684	202	FALSE	0	3.8798249	0.0129982	0.0106944	0.0024128	0
1591	1980	Female	501	118	FALSE	0	4.1855469	0	0	0	0
1592	1987	Female	1071	685	FALSE	0	5.234375	0.0234375	0.0570679	0	0.078125
1593	1985	Female	100	596	FALSE	0	4.1875	0.0234375	0.03479	0.015625	0.0234375
1594	1988	Female	644	103	FALSE	0	6.0693359	0.0238342	0.0623191	0.0166016	0.0040283
1595	1987	Female	950	1244	FALSE	0	5.4765625	0.2651367	0.0559739	0.0117188	0.0117188
1596	1986	Male	814	457	FALSE	0	5.6699219	0.0181885	0.0569969	0.0351563	0
1597	1978	Female	542	696	FALSE	0	3.09375	0	0	0	0
1598	1980	Male	644	1386	FALSE	0	5.4538574	0.0251656	0.0467597	0.0092773	0.0040283
1599	1987	Female	1071	1247	FALSE	0	5.5390625	0.034668	0.0648804	0.015625	0.078125
1600	1981	Male	132	596	FALSE	0	4.015625	0	0.0234375	0.015625	0.03125
1601	1991	Female	1400	1403	FALSE	0	6.5106812	0.1458397	0.0931421	0.0110779	0.0234375
1602	1990	Female	1228	1416	FALSE	0	6.3583374	0.0085063	0.1034506	0.0345459	0.0174866
1603	1995	Female	1399	1405	FALSE	0	6.5143738	0.0227695	0.1937094	0.2651367	0.0365677
1604	1990	Female	1228	1413	FALSE	0	6.1224976	0.0185785	0.0918148	0.0258789	0.0174866
1605	1988	Female	1071	966	FALSE	0	5.8046875	0.0394897	0.1026094	0.0258789	0.078125
1606	1993	Female	1401	1411	FALSE	0	5.6616211	0.0008545	0.0551587	0	0.0170898
1607	1990	Female	1262	1408	FALSE	0	6.7626953	0.0308189	0.1007947	0.0541992	0.0099792
1608	1988	Female	1265	1242	FALSE	0	6.4624023	0.0628357	0.1999951	0.0532227	0.1831665
1609	1999	Male	1407	1402	FALSE	0	6.9455261	0.0540786	0.0802078	0.003417	0.0150452
1610	1951	Male	694	736	FALSE	0	4.4624836	0.0119221	0.0222208	0	0.0135367
1611	1993	Female	1262	1405	FALSE	0	6.722168	0.0743408	0.1833597	0.2651367	0.0099792
1612	1989	Female	814	1254	FALSE	0	6.3081055	0.0125122	0.0675543	0.0449829	0
1613	1991	Female	1262	1406	FALSE	0	5.6494141	0.0120049	0.0510187	0	0.0099792
1614	1990	Female	1262	1406	FALSE	0	5.6494141	0.0120049	0.0510187	0	0.0099792
1615	2001	Female	104	962	TRUE	0.001367054	6.2495117	0.0013733	0.0639557	0.0273743	0.0146484
1616	2003	Female	1414	959	TRUE	0.001367054	6.5932617	0.07201	0.0544941	0.0014648	0.0150452
1617	1991	Female	814	1252	FALSE	0	6.5048218	0.0185099	0.1018535	0.093668	0
1618	1988	Female	1388	1256	FALSE	0	6.2528687	0.0611386	0.0929398	0.0376587	0.0422974
1619	1989	Male	700	1103	FALSE	0	5.4926758	0.0758209	0.0219264	0	0.0021973
1620	1976	Female	683	719	FALSE	0	2.421875	0	0	0	0

1621	1994	Female	104	1252	FALSE	0	6.164978	0.0172596	0.1000503	0.093668	0.0146484
1622	1995	Female	1115	1118	FALSE	0	6.7557983	0.0431027	0.0792312	0.0277424	0.0376587
1623	1995	Female	1115	1263	FALSE	0	6.6352539	0.0523758	0.0922035	0.0259399	0.0376587
1624	1960	Male	1415	736	FALSE	0	2.6342163	0	0.0126653	0	0
1625	1996	Female	104	962	FALSE	0	6.2495117	0.0013733	0.0639557	0.0273743	0.0146484
1626	1985	Female	676	1410	FALSE	0	2.1796875	0	0.0019531	0	0
1627	1985	Male	502	1413	FALSE	0	5.875	0.0662231	0.0903215	0.0258789	0.0742188
1628	1997	Female	1440	1434	FALSE	0	7.3026346	0.0251954	0.203397	0.1437531	0.0024304
1629	1989	Female	899	1389	FALSE	0	5.5060097	0.0031691	0.0227842	0.0022125	0.0055404
1630	1989	Male	899	896	FALSE	0	5.1935708	0.0106385	0.0175519	0.0081787	0.0055404
1631	1990	Female	850	812	FALSE	0	5.9663086	0.0147705	0.1015214	0.0976563	0.0008545
1632	1996	Female	1390	1432	FALSE	0	6.8544006	0.1700155	0.135702	0.0361557	0.0595665
1633	1994	Female	1264	1449	FALSE	0	6.8816223	0.005769	0.1708503	0.0055017	0.1074219
1634		Female	881	785	FALSE	0	6.8480024	0.0064511	0.0858248	0.0430012	0.040494
1635	1994	Male	1125	1417	FALSE	0	6.5615881	0.0067947	0.0745158	0.0009984	0.0286455
1636	1995	Male	1125	1420	FALSE	0	6.5532494	0.0026385	0.0821793	0.0342388	0.0286455
1637	1994	Female	1419	1418	FALSE	0	5.6282959	0.035728	0.0502737	0.0158615	0.0238037
1638	1996	Female	1125	1417	FALSE	0	6.5615881	0.0067947	0.0745158	0.0009984	0.0286455
1639	1998	Female	1286	140	FALSE	0	5.9850382	0.0166271	0.0715514	0	0.001301
1640	2005	Female	1286	1284	TRUE	0.001367054	6.4935139	0.1352	0.0551757	0.0195824	0.001301
1641	2005	Female	1414	1441	TRUE	0.001367054	7.0114746	0.0787144	0.0764465	0.0018921	0.0150452
1642	2003	Female	1143	1409	TRUE	0.001367054	7.1228027	0.0588112	0.132429	0.1049194	0.0083008
1643	1998	Female	974	1392	FALSE	0	4.7813254	0.0028529	0.0195649	0	0.003418
1644	1991	Female	104	1426	FALSE	0	6.4230347	0.0806446	0.0644032	0.0226765	0.0146484
1645	1990	Female	850	101	FALSE	0	5.4545898	0.02005	0.029085	0.03125	0.0008545
1646	1988	Female	1126	1446	FALSE	0	5.1726074	0.0004101	0.0361451	0	0.0300903
1647	1994	Female	1431	1404	FALSE	0	7.1586914	0.0753174	0.2267851	0.2651367	0.043026
1648	2003	Male	1435	1429	TRUE	0.001367054	6.3005385	0.0522922	0.0706056	0.0471994	0.0152156
1649	1997	Female	1120	1138	FALSE	0	6.4893188	0.0298486	0.1996485	0.2573338	0.0561523
1650	2004	Female	1394	1439	TRUE	0.001367054	6.1176047	0.0120383	0.0259022	0.0026801	0.0092597
1651	1997	Female	1140	1254	FALSE	0	6.6008301	0.0699615	0.0665359	0.0449829	0.010376
1652	1997	Male	1436	1242	FALSE	0	6.6381531	0.053371	0.1209933	0.0532227	0.0120339
1653	1992	Female	909	765	FALSE	0	5.1181641	0.013916	0.0299192	0.0007324	0.0236816
1654		Female	0	0	FALSE	0	0	0	0		
1655	1999	Male	1423	1433	FALSE	0	7.2346191	0.0803757	0.1842885	0.0628357	0.0371399
1656	1970	Female	1038	783	FALSE	0	4.3046875	0.0009766	0.0362043	0.0429688	0
1657	1996	Female	909	1059	FALSE	0	5.1269531	0.0072021	0.0329713	0.0078125	0.0236816
1658	2001	Female	1040	1091	TRUE	0.001367054	5.7413025	0.0148153	0.0428351	0.0225983	0.0139313
1659	1984	Female	1038	101	FALSE	0	4.2734375	0.0009766	0.015625	0.03125	0
1660	1997	Male	909	1059	FALSE	0	5.1269531	0.0072021	0.0329713	0.0078125	0.0236816
1661	1999	Female	1058	1153	FALSE	0	7.3142061	0.010897	0.0736499	0.0064318	0.0142442
1662	1996	Female	1302	1422	FALSE	0	6.5146849	0.0386327	0.1038629	0.0009765	0.0037593
1663	1996	Female	1058	1451	FALSE	0	7.3666658	0.0113038	0.0706807	0.0064511	0.0142442
1664	1996	Female	1125	1154	FALSE	0	7.0343489	0.0389626	0.0871895	0.0026394	0.0286455
1665	2005	Female	1291	1091	TRUE	0.001367054	6.3691478	0.0178537	0.0539275	0.0225983	0.0142442
1666	1998	Male	1058	1417	FALSE	0	6.9951318	0.0053417	0.0731513	0.0009984	0.0142442
1667	1998	Male	1023	1424	FALSE	0	6.4042738	0.0075471	0.0693774	0.0009984	0.0005035
1668	2004	Female	1297	1159	TRUE	0.001367054	7.322113	0.0750617	0.1001357	0.069519	0.0266709
1669	2004	Female	1297	1443	TRUE	0.001367054	7.4493713	0.1012992	0.0859505	0.0226765	0.0266709
1670	2012	Male	1297	1453	TRUE	0.001367054	7.7505798	0.0910081	0.1405855	0.10779	0.0266709
1671	2001	Female	1040	1298	TRUE	0.001367054	6.7028809	0.0076851	0.0723939	0.0715218	0.0139313
1672	2002	Female	1040	1462	TRUE	0.001367054	6.745575	0.0063049	0.075511	0.0544951	0.0139313
1673	2002	Female	1040	959	TRUE	0.001367054	5.8807373	0.0107417	0.0255342	0.0014648	0.0139313
1674	2002	Female	1040	1412	TRUE	0.001367054	6.3421631	0.0044289	0.0564144	0.0150452	0.0139313
1675	2000	Male	1023	1000	TRUE	0.001367054	5.3830566	0.0083618	0.024952	0.0078125	0.0005035
1676	1995	Female	1459	1084	FALSE	0	6.5910034	0.0314949	0.0935076	0.0422974	0.0016708

1677	1998	Female	0	0	FALSE	0	0	0	0		
1678	1994	Male	909	1450	FALSE	0	5.7519531	0.0166931	0.0522138	0.0018311	0.0236816
1679	2002	Female	1040	1461	TRUE	0.001367054	6.7295151	0.0101728	0.0744496	0.0052921	0.0139313
1680	2002	Male	1027	1194	TRUE	0.001367054	6.4206195	0.0119151	0.0433175	0.0102167	0
1681	2011	Female	1317	1479	TRUE	0.001367054	6.8922728	0.0311776	0.0565018	0.0158813	0.016344
1682	1996	Female	1480	1406	FALSE	0	5.2290039	0.0003471	0.0407619	0	0.0072021
1683	2007	Female	1318	1279	TRUE	0.001367054	6.7299034	0.0105213	0.0626397	0.0025846	0.0039026
1684	2009	Male	1205	1483	TRUE	0.001367054	4.0984645	0	0.0421103	0	0.0248988
1685	1989	Female	1460	1213	FALSE	0	4.4973269	0.0061106	0.0124721	0.0006437	0
1686	2007	Female	1318	765	TRUE	0.001367054	5.5224836	0.0037445	0.0245495	0.0007324	0.0039026
1687	2003	Female	1197	999	TRUE	0.001367054	7.3344846	0.0161406	0.0734745	0.0064511	0.0181582
1688	2003	Male	1197	840	TRUE	0.001367054	6.395422	0.0079816	0.0344549	0.0028568	0.0181582
1689	2007	Female	1140	1473	TRUE	0.001367054	6.7460327	0.04261	0.0566824	0.0063114	0.010376
1690	2003	Female	1140	1478	TRUE	0.001367054	6.815918	0.0672188	0.0434951	0.0053711	0.010376
1691	2004	Male	1197	840	TRUE	0.001367054	6.395422	0.0079816	0.0344549	0.0028568	0.0181582
1692	1999	Female	1286	1466	FALSE	0	6.9518533	0.0907656	0.0651171	0.0515657	0.001301
1693	2000	Male	1187	1424	TRUE	0.001367054	6.8389245	0.008369	0.0710178	0.0009984	0.0071462
1694	2000	Female	1023	1176	TRUE	0.001367054	6.0926047	0.0111995	0.0402447	0.0098812	0.0005035
1695	2006	Female	1291	1176	TRUE	0.001367054	6.6834627	0.0471884	0.0440185	0.0098812	0.0142442
1696	2001	Female	1286	1466	TRUE	0.001367054	6.9518533	0.0907656	0.0651171	0.0515657	0.001301
1697	1981	Female	1182	1476	FALSE	0	2.3339844	0	0	0	0
1698	2000	Female	1304	1448	TRUE	0.001367054	6.8870983	0.0104639	0.0732297	0.0044745	0.0096436
1699	1992	Female	850	1168	FALSE	0	5.5795898	0.0241699	0.0297869	0	0.0008545
1700	2002	Female	1027	1176	TRUE	0.001367054	6.0299521	0.0663502	0.0277514	0.0098812	0
1701	2010	Female	1323	1309	TRUE	0.001367054	7.2989074	0.0134878	0.0699664	0.0007337	0.0037206
1702	2009	Male	1197	840	TRUE	0.001367054	6.395422	0.0079816	0.0344549	0.0028568	0.0181582
1703	2002	Female	1455	1470	TRUE	0.001367054	6.1322192	0.0162979	0.0551245	0.0039301	0.0163219
1704	2010	Female	1205	1473	TRUE	0.001367054	7.0146632	0.0154457	0.075468	0.0063114	0.0248988
1705	2013	Female	1205	1464	TRUE	0.001367054	7.3860469	0.0105538	0.1020732	0.0413141	0.0248988
1706	2005	Female	1206	1471	TRUE	0.001367054	7.6189779	0.0474121	0.0693474	0.014887	0.0209387
1707	2011	Female	1205	1136	TRUE	0.001367054	6.5779567	0.0169171	0.0594877	0.0026855	0.0248988
1708	2009	Male	1205	1484	TRUE	0.001367054	7.0202479	0.0120457	0.0809266	0.0005069	0.0248988
1709	2008	Female	1469	1458	TRUE	0.001367054	7.2562092	0.0935644	0.1681818	0.1352	0.0632836
1710	2009	Female	1469	1457	TRUE	0.001367054	7.2562092	0.0935644	0.1681818	0.1352	0.0632836
1711	1988	Female	125	1465	FALSE	0	5.3080444	0.0258827	0.0141769	0.0007019	0.0078125
1712	2006	Male	1481	1445	TRUE	0.001367054	7.3395081	0.0441696	0.1347604	0.0001297	0.000665
1713	2013	Female	1454	1475	TRUE	0.001367054	7.6855657	0.0503962	0.0872875	0.0022264	0.0158577
1714	1989	Male	1377	1413	FALSE	0	5.5566406	0.0011597	0.058214	0.0258789	0.0234375
1715	1989	Male	1377	1447	FALSE	0	5.9355469	0.0005798	0.081696	0.0394897	0.0234375
1716	1989	Female	1377	1251	FALSE	0	5.4423828	0.0023193	0.0309477	0.0040283	0.0234375
1717	1989	Male	1377	1396	FALSE	0	5.2207031	0	0.0252991	0.0175781	0.0234375
1718	2016	Female	1508	1327	TRUE	0.001367054	8.182561	0.0158758	0.1539202	0.0334404	0.0120457
1719	2006	Female	1197	1375	TRUE	0.001367054	6.7649755	0.0024699	0.0730489	0.0175781	0.0181582
1720	2008	Female	1344	1442	TRUE	0.001367054	7.7235014	0.0258458	0.1033143	0.0172596	0.0158104
1721	2000	Female	0	0	TRUE	0.001367054	0	0	0		
1722	2011	Female	1505	1509	TRUE	0.001367054	7.1835266	0.035853	0.0540435	0.0307782	0.0078658
1723	2011	Male	1205	1495	TRUE	0.001367054	7.9829249	0.0125601	0.1348904	0.0872439	0.0248988
1724	2008	Male	1492	1437	TRUE	0.001367054	7.8331511	0.0142475	0.1090776	0.0578156	0.0158104
1725	2010	Female	1205	1498	TRUE	0.001367054	7.6194389	0.0230972	0.0788172	0.0117696	0.0248988
1726	2009	Female	1467	1333	TRUE	0.001367054	7.056366	0.071789	0.0657452	0.0127563	0.0039563
1727	2015	Female	1504	1309	TRUE	0.001367054	6.8633093	0.0036419	0.0552267	0.0007337	0.0016036
1728	2014	Female	1205	1380	TRUE	0.001367054	7.5704036	0.0466036	0.0927849	0.0406383	0.0248988
1729	2014	Male	1502	1500	TRUE	0.001367054	7.5444732	0.0103261	0.0586137	0.0099182	0.0134878
1730	2012	Female	1507	1488	TRUE	0.001367054	6.2201868	0.0051007	0.0460465	0	0.0065747
1731	2008	Female	1205	1358	TRUE	0.001367054	7.3087743	0.0179523	0.0694685	0.0119151	0.0248988
1732	2010	Female	1379	1501	TRUE	0.001367054	7.3600558	0.0115946	0.066724	0.008806	0.0171319

1733	2010	Female	1369	1463	TRUE	0.001367054	7.7435262	0.0124346	0.1235219	0.10779	0.0100948
1734	1995	Female	1058	1366	FALSE	0	5.6096568	0.0009323	0.0560696	0.0625	0.0142442
1735	2011	Male	1507	1298	TRUE	0.001367054	7.5309168	0.0102488	0.0852609	0.0715218	0.0065747
1736	2016	Male	1379	1486	TRUE	0.001367054	6.0460413	0.0174069	0.0523956	0	0.0171319
1737	2010	Male	1379	1308	TRUE	0.001367054	7.4353263	0.010716	0.0753269	0.0064511	0.0171319
1738	2002	Female	1197	1336	TRUE	0.001367054	6.2972021	0.0204043	0.0536143	0.0083008	0.0181582
1739	2011	Female	1328	1303	TRUE	0.001367054	7.1103284	0.0115689	0.0544355	0.0005951	0.0135524
1740	2003	Male	1510	765	TRUE	0.001367054	5.8753759	0.0049152	0.0526879	0.0007324	0.0191974
1741	2004	Female	814	1370	TRUE	0.001367054	6.3269043	0.0115356	0.0509615	0.0241699	0
1742	2006	Female	1314	1373	TRUE	0.001367054	7.057251	0.0525908	0.0672389	0.0170898	0.0082101
1743	2009	Female	1314	1345	TRUE	0.001367054	7.3457031	0.0651109	0.0622629	0.005188	0.0082101
1744	2010	Male	1297	1345	TRUE	0.001367054	7.3626404	0.1348806	0.0676436	0.005188	0.0266709
1745	1986	Female	1078	1489	FALSE	0	4.4705811	4.768E-05	0.0198534	0	0.0045166
1746	2004	Female	1197	1336	TRUE	0.001367054	6.2972021	0.0204043	0.0536143	0.0083008	0.0181582
1747	2005	Male	1291	1347	TRUE	0.001367054	7.2062924	0.0292342	0.0504983	0.0098865	0.0142442
1748	2005	Female	1197	1336	TRUE	0.001367054	6.2972021	0.0204043	0.0536143	0.0083008	0.0181582
1749	1999	Female	1452	1341	FALSE	0	6.5321104	0.0173889	0.0484412	0.0100372	0.0142091
1750	2015	Male	1430	1485	TRUE	0.001367054	7.1822804	0.0264095	0.0647651	0.0104691	0.0111939
1751	2006	Female	1510	1482	TRUE	0.001367054	6.721079	0.0061956	0.0718018	0.0072021	0.0191974
1752	2009	Female	1496	1412	TRUE	0.001367054	7.3977051	0.0868206	0.0811423	0.0150452	0.037426
1753	2009	Female	1496	1373	TRUE	0.001367054	7.2209473	0.074173	0.0669876	0.0170898	0.037426
1754	2014	Male	1430	1358	TRUE	0.001367054	6.7019992	0.0185659	0.0528459	0.0119151	0.0111939
1755	2007	Male	1291	1347	TRUE	0.001367054	7.2062924	0.0292342	0.0504983	0.0098865	0.0142442
1756	2001	Male	1497	1472	TRUE	0.001367054	5.7817293	0.009695	0.0248482	0.0053463	0.0103025
1757	2004	Female	1491	1272	TRUE	0.001367054	7.0558167	0.0399272	0.1208512	0.0107422	0.038833
1758	2009	Female	1291	1347	TRUE	0.001367054	7.2062924	0.0292342	0.0504983	0.0098865	0.0142442
1759	2003	Female	1278	1341	TRUE	0.001367054	6.6890454	0.0492846	0.0627612	0.0100372	0.0132758
1760	2003	Male	1354	1438	TRUE	0.001367054	5.5229394	0.0066071	0.0350322	0.0061188	0.0322151
1761	2004	Male	1499	1425	TRUE	0.001367054	4.4169922	0.0020294	0.0082476	0	0
1762		Male	1338	1456	FALSE	0	5.6514893	0.0112391	0.0304997	0.0088196	0.0176086
1763	1988	Female	1377	1411	FALSE	0	4.8144531	0.027832	0.0282974	0	0.0234375
1764	2008	Female	1503	1025	TRUE	0.001367054	7.1639396	0.0493141	0.0610908	0.0163569	0.0133812
1765	2012	Female	1507	1490	TRUE	0.001367054	7.9113462	0.0208251	0.0761262	0.0141886	0.0065747
1766	2012	Female	1507	1477	TRUE	0.001367054	7.2266667	0.0142754	0.0585409	0.012702	0.0065747
1767	2015	Female	1533	1528	TRUE	0.001367054	8.7276013	0.1006572	0.1744111	0.081022	0.0090883
1768	2012	Female	1561	1512	TRUE	0.001367054	7.6360894	0.0115958	0.0651093	0.0164408	0.0138588
1769	2013	Female	1507	1557	TRUE	0.001367054	7.2372003	0.0127126	0.0701384	0.0337634	0.0065747
1770	2013	Male	1379	1560	TRUE	0.001367054	7.0705128	0.0182211	0.0537432	0.0062427	0.0171319
1771	2012	Female	1467	1516	TRUE	0.001367054	7.5900687	0.0403073	0.073075	0.0142757	0.0039563
1772	1990	Female	657	1563	FALSE	0	3.3515625	0	0.0078125	0	0
1773	1994	Female	1555	1428	FALSE	0	6.8374696	0.0042548	0.055393	0.0226765	0.0074227
1774	2002	Male	1494	1532	TRUE	0.001367054	7.2438992	0.0137823	0.0747483	0.0008886	0.0082214
1775	2016	Female	1430	1525	TRUE	0.001367054	7.2747923	0.013938	0.0792949	0.0252831	0.0111939
1776	2007	Female	1548	1194	TRUE	0.001367054	7.3508635	0.0149614	0.0655862	0.0102167	0.0267128
1777	2013	Female	1468	1519	TRUE	0.001367054	6.7154083	0.0038638	0.0907934	0.0399272	0.0063477
1778	2015	Female	1430	1559	TRUE	0.001367054	6.3069348	0.0272039	0.0377123	0.0073708	0.0111939
1779	1997	Female	1514	1130	FALSE	0	7.2540314	0.0091909	0.1196062	0.0662231	0.0036822
1780	2017	Male	1430	1527	TRUE	0.001367054	7.2905046	0.0135489	0.1073072	0.0196607	0.0111939
1781	1960	Male	0	0	FALSE	0	0	0	0		
1782	1996	Female	1515	1397	FALSE	0	6.3330078	0.0472412	0.0792105	0.0639648	0.0023193
1783	2016	Male	1533	1457	TRUE	0.001367054	8.2804737	0.0166911	0.1671653	0.1352	0.0090883
1784	1995	Female	1515	1095	FALSE	0	5.7392578	0.0771484	0.0309096	0.0390625	0.0023193
1785	1998	Female	0	0	FALSE	0	0	0	0		
1786	2009	Female	1541	1327	TRUE	0.001367054	8.2755832	0.0432047	0.1470496	0.0334404	0.0292342
1787	2009	Male	1291	1529	TRUE	0.001367054	7.2100861	0.0042112	0.063071	0.0237257	0.0142442
1788	1997	Female	1515	1421	FALSE	0	7.0268555	0.0323601	0.075632	0.0308189	0.0023193

1789	2015	Male	1362	1512	TRUE	0.001367054	7.8394252	0.0115856	0.0760388	0.0164408	0.0277774
1790	1998	Female	1515	1095	FALSE	0	5.7392578	0.0771484	0.0309096	0.0390625	0.0023193
1791	2012	Male	1537	1487	TRUE	0.001367054	8.4116211	0.1061264	0.1510832	0.0695984	0.0734531
1792	1997	Male	1125	1517	FALSE	0	6.234951	0.0008292	0.0725798	0.0009766	0.0286455
1793	2013	Female	1523	1526	TRUE	0.001367054	8.1921692	0.0325222	0.0995378	0.0124522	0.0428074
1794	1996	Male	1520	1518	FALSE	0	4.2758727	0.0004883	0.0095598	0.0088584	0
1795	1998	Female	1511	1522	FALSE	0	6.470747	0.0257114	0.0604839	0.0008886	0.0198135
1796	2018	Male	1430	1521	TRUE	0.001367054	7.2503474	0.0140485	0.0733241	0.0160119	0.0111939
1797	2010	Female	1314	1536	TRUE	0.001367054	7.7437592	0.0475638	0.1150465	0.0863297	0.0082101
1798	2009	Male	1558	1552	TRUE	0.001367054	5.83744	0.0034083	0.0372619	0.0033352	0
1799	2009	Female	1542	1554	TRUE	0.001367054	7.143119	0.0235567	0.0769352	0.0336794	0.0136385
1800	2019	Male	1507	1553	TRUE	0.001367054	6.9790064	0.0129098	0.0526467	0.0060093	0.0065747
1801	1974	Male	386	1564	FALSE	0	4.515625	0.0263672	0.016571	0	0.0019531
1802	2006	Female	1547	1493	TRUE	0.001367054	7.4741019	0.008765	0.0553779	0.0079816	0.0355812
1803	2000	Male	844	1530	TRUE	0.001367054	5.6762543	0.0326812	0.0509044	0.0082753	0.0220947
1804	1964	Male	0	0	FALSE	0	0	0	0		
1805	1991	Male	899	1562	FALSE	0	5.2543618	0.0378542	0.0236561	0.0161133	0.0055404
1806	2012	Female	1544	1458	TRUE	0.001367054	7.4114486	0.0102499	0.127442	0.1352	0.0222797
1807	2007	Female	1547	840	TRUE	0.001367054	6.7613295	0.007105	0.0411387	0.0028568	0.0355812
1808	2014	Female	1430	1531	TRUE	0.001367054	7.0952488	0.0175053	0.059876	0.0107064	0.0111939
1809	2007	Female	1547	1493	TRUE	0.001367054	7.4741019	0.008765	0.0553779	0.0079816	0.0355812
1810	2009	Female	1297	1539	TRUE	0.001367054	7.2759094	0.0803264	0.0693672	0.0106437	0.0266709
1811	2008	Male	1197	1543	TRUE	0.001367054	6.8449725	0.0103981	0.0505125	0.0134497	0.0181582
1812	1964	Female	0	0	FALSE	0	0	0	0		
1813	2016	Female	1504	1559	TRUE	0.001367054	6.411412	0.0051604	0.0224755	0.0073708	0.0016036
1814	2003	Female	1535	1474	TRUE	0.001367054	5.3950195	0.0031357	0.0146483	0.0044717	0
1815	2014	Female	1538	1444	TRUE	0.001367054	7.2074585	0.0354408	0.055089	0.0013733	0.0027952
1816	1979	Male	1427	1540	FALSE	0	4.2944336	0.0263062	0.0150218	0	0.0058594
1817	2007	Female	1545	1550	TRUE	0.001367054	6.7752185	0.0337498	0.0431087	0.016222	0.0068806
1818	2007	Female	1317	1546	TRUE	0.001367054	6.8329306	0.0115148	0.0405134	0.0081406	0.016344
1819	1997	Female	1390	1513	FALSE	0	6.5566101	0.0094719	0.1178182	0.0009766	0.0595665
1820	2011	Male	1561	1524	TRUE	0.001367054	6.4300976	0.0074084	0.048489	0.0008886	0.0138588
1821	2007	Female	1506	1534	TRUE	0.001367054	6.1176987	0.0426845	0.0537373	0.0392628	0.0097089
1822	2011	Female	1507	1554	TRUE	0.001367054	7.6198365	0.0305687	0.075552	0.0336794	0.0065747
1823	2011	Male	1379	1551	TRUE	0.001367054	7.1355603	0.02511	0.0660385	0.0161836	0.0171319
1824	2011	Female	1507	1549	TRUE	0.001367054	7.0593475	0.015133	0.0533414	0.009571	0.0065747
1825	2011	Male	1561	1556	TRUE	0.001367054	7.1996731	0.0452458	0.0576171	0.0150617	0.0138588
1826	2012	Female	1561	1560	TRUE	0.001367054	6.9157448	0.0082034	0.0417559	0.0062427	0.0138588
1827	2012	Female	1507	1553	TRUE	0.001367054	6.9790064	0.0129098	0.0526467	0.0060093	0.0065747
1828	2012	Female	1561	1025	TRUE	0.001367054	6.6578572	0.0165755	0.0501574	0.0163569	0.0138588
1829	1945	Female	0	0	FALSE	0	0	0	0		
1830	1973	Male	0	0	FALSE	0	0	0	0		
1831	1965	Male	0	0	FALSE	0	0	0	0		
1832	1984	Female	100	98	FALSE	0	4.3945313	0.0146484	0.0308838	0	0.0234375
1833	1985	Male	100	1568	FALSE	0	2.671875	0	0.0269775	0	0.0234375
1834	1989	Male	100	1566	FALSE	0	2.671875	0	0.0269775	0	0.0234375
1835	1981	Female	132	777	FALSE	0	4.59375	0.2753906	0.0482788	0.0351563	0.03125
1836	1994	Male	104	103	FALSE	0	5.8574219	0.0010376	0.0665837	0.0166016	0.0146484
1837	1979	Female	132	118	FALSE	0	4	0.015625	0.015625	0	0.03125
1838	1990	Female	657	1387	FALSE	0	5.2712402	0.1431427	0.0565415	0.0480957	0
1839	1988	Male	100	1075	FALSE	0	5.0915527	0.03302	0.0757066	0.0480957	0.0234375
1840	1976	Male	132	780	FALSE	0	4.7946777	0.0240479	0.0447996	0.0229492	0.03125
1841	1986	Female	125	1387	FALSE	0	5.4587402	0.0351715	0.0526353	0.0480957	0.0078125
1842	1986	Female	1078	541	FALSE	0	4.5067139	0.0022125	0.0198534	0	0.0045166
1843	1985	Male	100	926	FALSE	0	5.1981201	0.0174866	0.0763958	0.041626	0.0234375
1844	1983	Female	100	925	FALSE	0	5.1981201	0.0174866	0.0763958	0.041626	0.0234375

1845	1991	Male	104	1573	FALSE	0	6.0477295	0.0317726	0.098825	0.0523071	0.0146484
1846	1990	Female	1579	140	FALSE	0	5.9277344	0.0532227	0.2040268	0	0.2636719
1847	1991	Male	1228	1571	FALSE	0	5.6928101	0.0849075	0.0789271	0.0351563	0.0174866
1848	1988	Female	657	1578	FALSE	0	5.4447632	0.1021919	0.0441773	0.0444565	0
1849	1987	Male	662	457	FALSE	0	5.5722656	0.0437012	0.0572351	0.0351563	0.0009766
1850	1992	Male	1390	920	FALSE	0	5.9635925	0.2885723	0.0965385	0.0351563	0.0595665
1851	1980	Male	127	675	FALSE	0	3.203125	0.0078125	0.03125	0	0.0625
1852	1996	Male	251	0	FALSE	0	2.7784711	0	0.012921		0.0036439
1853	1956	Male	257	236	FALSE	0	3.515625	0.25	0	0	0
1854	1990	Male	1228	1396	FALSE	0	5.7865601	0.0115128	0.0588999	0.0175781	0.0174866
1855	1985	Female	351	1574	FALSE	0	5.6052246	0.0165405	0.115739	0.1030273	0.0634766
1856	1984	Female	351	1574	FALSE	0	5.6052246	0.0165405	0.115739	0.1030273	0.0634766
1857	1983	Male	351	1567	FALSE	0	5.0683594	0.0089111	0.0473633	0.03125	0.0634766
1858	1987	Female	351	1574	FALSE	0	5.6052246	0.0165405	0.115739	0.1030273	0.0634766
1859	1987	Female	351	1385	FALSE	0	5.8061523	0.0213623	0.0729254	0.0185547	0.0634766
1860	1985	Female	351	1567	FALSE	0	5.0683594	0.0089111	0.0473633	0.03125	0.0634766
1861	1985	Female	351	325	FALSE	0	4.3183594	0.0030518	0.0317383	0	0.0634766
1862	1986	Female	351	1385	FALSE	0	5.8061523	0.0213623	0.0729254	0.0185547	0.0634766
1863	1987	Male	351	1567	FALSE	0	5.0683594	0.0089111	0.0473633	0.03125	0.0634766
1864	1985	Female	351	1385	FALSE	0	5.8061523	0.0213623	0.0729254	0.0185547	0.0634766
1865	1988	Female	351	1567	FALSE	0	5.0683594	0.0089111	0.0473633	0.03125	0.0634766
1866	1986	Female	351	1567	FALSE	0	5.0683594	0.0089111	0.0473633	0.03125	0.0634766
1867	1980	Female	351	1574	FALSE	0	5.6052246	0.0165405	0.115739	0.1030273	0.0634766
1868	1986	Female	351	1574	FALSE	0	5.6052246	0.0165405	0.115739	0.1030273	0.0634766
1869	1987	Female	351	325	FALSE	0	4.3183594	0.0030518	0.0317383	0	0.0634766
1870	1988	Male	351	1584	FALSE	0	6.182373	0.2765503	0.0781032	0.0213623	0.0634766
1871	1990	Male	351	1585	FALSE	0	5.4384766	0.267395	0.0490849	0.0030518	0.0634766
1872	1983	Female	408	0	FALSE	0	1.25	0	0		0
1873	1991	Male	1071	685	FALSE	0	5.234375	0.0234375	0.0570679	0	0.078125
1874	1982	Male	502	660	FALSE	0	5.390625	0.0302734	0.0486862	0.0078125	0.0742188
1875	1985	Male	1565	1098	FALSE	0	3.0556313	4.972E-08	0.0051643	0	0
1876	1985	Male	1071	685	FALSE	0	5.234375	0.0234375	0.0570679	0	0.078125
1877	1980	Female	501	98	FALSE	0	4.4082031	0.0007324	0.0039063	0	0
1878	1990	Male	1228	822	FALSE	0	6.0834351	0.0361347	0.1132097	0.0722656	0.0174866
1879	1989	Female	1569	1591	FALSE	0	4.0927734	0.0039063	0.0078125	0	0
1880	1991	Female	814	1577	FALSE	0	6.0633545	0.0277424	0.0871247	0.041626	0
1881	1988	Female	1071	1246	FALSE	0	5.5185547	0.0345459	0.0826645	0.0361328	0.078125
1882	1991	Female	1228	1247	FALSE	0	5.8568726	0.018795	0.0540857	0.015625	0.0174866
1883	1989	Male	1228	822	FALSE	0	6.0834351	0.0361347	0.1132097	0.0722656	0.0174866
1884	1990	Female	1228	1251	FALSE	0	6.0082397	0.0478106	0.0645485	0.0040283	0.0174866
1885	1982	Female	502	596	FALSE	0	4.8671875	0	0.0525925	0.015625	0.0742188
1886	1991	Female	814	923	FALSE	0	5.8925781	0.0117798	0.0407314	0.0019531	0
1887	1991	Male	1228	1246	FALSE	0	5.8363647	0.003417	0.0718699	0.0361328	0.0174866
1888	1983	Male	644	926	FALSE	0	5.9354248	0.093668	0.0676936	0.041626	0.0040283
1889	1984	Female	644	928	FALSE	0	5.7127686	0.0704441	0.0381287	0.0045166	0.0040283
1890	1984	Male	644	1591	FALSE	0	5.5019531	0.1323547	0.0182753	0	0.0040283
1891	1985	Male	644	101	FALSE	0	5.4482422	0.0391235	0.0339003	0.03125	0.0040283
1892	1988	Male	814	812	FALSE	0	6.0878906	0.0636597	0.1124045	0.0976563	0
1893	1986	Male	644	1075	FALSE	0	5.8288574	0.0798531	0.0670043	0.0480957	0.0040283
1894	1986	Male	644	815	FALSE	0	6.1044922	0.0273743	0.0570182	0.0302734	0.0040283
1895	1986	Male	644	1577	FALSE	0	5.9354248	0.0965977	0.0810569	0.041626	0.0040283
1896	1986	Male	814	925	FALSE	0	6.0633545	0.0277424	0.0737614	0.041626	0
1897	1987	Male	644	1587	FALSE	0	3.4091797	0	0.0182753	0	0.0040283
1898	1987	Male	814	1075	FALSE	0	5.9567871	0.0372963	0.0730721	0.0480957	0
1899	1987	Male	814	1577	FALSE	0	6.0633545	0.0277424	0.0871247	0.041626	0
1900	1988	Female	1071	1107	FALSE	0	5.765625	0.0330811	0.1240044	0.0722656	0.078125

1901	1988	Male	644	98	FALSE	0	5.1318359	0.0014648	0.0221815	0	0.0040283
1902	1988	Male	644	101	FALSE	0	5.4482422	0.0391235	0.0339003	0.03125	0.0040283
1903	1971	Not Recorded	1588	642	FALSE	0	2.4921875	0	0.0039063	0	0
1904	1990	Male	814	1573	FALSE	0	6.3875732	0.0391254	0.1006281	0.0523071	0
1905	1989	Male	644	1586	FALSE	0	3.4091797	0	0.0182753	0	0.0040283
1906	1989	Male	814	1577	FALSE	0	6.0633545	0.0277424	0.0871247	0.041626	0
1907	2002	Male	1399	1592	TRUE	0.001367054	6.39328	0.1434002	0.0801583	0.0234375	0.0365677
1908	1990	Male	814	926	FALSE	0	6.0633545	0.0277424	0.0737614	0.041626	0
1909	1990	Male	814	1229	FALSE	0	6.0546875	0.0150452	0.0714034	0.0009766	0
1910	1990	Male	104	815	FALSE	0	5.8925781	0.0010376	0.0612828	0.0302734	0.0146484
1911	1989	Male	1400	1250	FALSE	0	6.1229248	0.0104294	0.0796303	0.0004196	0.0234375
1912	1987	Female	644	1386	FALSE	0	5.4538574	0.0251656	0.0467597	0.0092773	0.0040283
1913	1985	Male	950	541	FALSE	0	4.4414063	0	0.027987	0	0.0117188
1914	1983	Male	692	667	FALSE	0	4.3144531	0.0332031	0.0194092	0	0.0078125
1915	1985	Male	644	546	FALSE	0	5.6162109	0.0437317	0.0395308	0.0234375	0.0040283
1916	1984	Male	950	1244	FALSE	0	5.4765625	0.2651367	0.0559739	0.0117188	0.0117188
1917	1983	Male	1071	822	FALSE	0	5.765625	0.0330811	0.1240044	0.0722656	0.078125
1918	1988	Male	644	660	FALSE	0	5.4482422	0.0376587	0.0221815	0.0078125	0.0040283
1919	1989	Female	351	1589	FALSE	0	5.2871094	0.0330811	0.0395508	0.015625	0.0634766
1920	1988	Female	351	1589	FALSE	0	5.2871094	0.0330811	0.0395508	0.015625	0.0634766
1921	1985	Male	1071	822	FALSE	0	5.765625	0.0330811	0.1240044	0.0722656	0.078125
1922	1988	Female	814	1113	FALSE	0	5.9677734	0.0099792	0.050979	0.0020142	0
1923	1991	Male	1071	966	FALSE	0	5.8046875	0.0394897	0.1026094	0.0258789	0.078125
1924	1990	Male	769	928	FALSE	0	6.0882568	0.0342388	0.080508	0.0045166	0.055603
1925	1995	Female	1399	1404	FALSE	0	6.5143738	0.0227695	0.1937094	0.2651367	0.0365677
1926	1995	Male	1399	1595	FALSE	0	6.5143738	0.0227695	0.1937094	0.2651367	0.0365677
1927	1990	Male	769	457	FALSE	0	5.9174805	0.0105286	0.0933084	0.0351563	0.055603
1928	1988	Male	1265	1257	FALSE	0	6.6748047	0.1437531	0.2306966	0.0598145	0.1831665
1929	1994	Female	769	930	FALSE	0	6.5512695	0.0203743	0.1094118	0.0427246	0.055603
1930	1991	Female	1115	1261	FALSE	0	6.7049561	0.1900568	0.0998651	0.0555	0.0376587
1931	1994	Female	1401	1571	FALSE	0	5.9741211	0.0761719	0.0721876	0.0351563	0.0170898
1932	1995	Female	1399	1406	FALSE	0	5.4416199	0.0065324	0.0613683	0	0.0365677
1933	1992	Female	1401	1416	FALSE	0	6.6396484	0.0406647	0.0967111	0.0345459	0.0170898
1934	1991	Male	769	930	FALSE	0	6.5512695	0.0203743	0.1094118	0.0427246	0.055603
1935	1991	Male	971	1575	FALSE	0	6.051009	0.0029894	0.0550396	0.0351715	0.0124893
1936	1995	Female	769	1085	FALSE	0	6.656189	0.022357	0.1034172	0.0430012	0.055603
1937	1992	Female	971	1583	FALSE	0	4.1966389	4.972E-08	0.0120619	0	0.0124893
1938	1995	Female	1264	1227	FALSE	0	6.7053833	0.0105004	0.1926775	0.0809336	0.1074219
1939	1991	Male	1262	1095	FALSE	0	6.0776367	0.0271606	0.049756	0.0390625	0.0099792
1940	1992	Male	769	784	FALSE	0	6.1479492	0.0301666	0.1269121	0.1152344	0.055603
1941	1991	Female	700	1591	FALSE	0	5.5551758	0.0665436	0.0141139	0	0.0021973
1942	1990	Male	971	1231	FALSE	0	5.9304646	0.0024304	0.063231	0.0581741	0.0124893
1943	1992	Female	1125	1232	FALSE	0	6.342556	0.0044745	0.1001361	0.0676155	0.0286455
1944	1989	Male	1400	1403	FALSE	0	6.5106812	0.1458397	0.0931421	0.0110779	0.0234375
1945	1991	Male	427	1597	FALSE	0	4.4921875	0.0166016	0.0194092	0	0.0078125
1946	1996	Female	1125	1085	FALSE	0	6.3806419	0.0129225	0.0689466	0.0430012	0.0286455
1947	1998	Female	1399	1599	FALSE	0	6.5456238	0.0784101	0.0892239	0.034668	0.0365677
1948	1994	Male	1125	761	FALSE	0	5.8293873	0.0128197	0.06129	0.0021395	0.0286455
1949	1998	Female	1125	1085	FALSE	0	6.3806419	0.0129225	0.0689466	0.0430012	0.0286455
1950	1995	Male	1125	785	FALSE	0	6.3806419	0.0129225	0.0689466	0.0430012	0.0286455
1951	1985	Female	1600	1107	FALSE	0	5.4921875	0.0512695	0.0786552	0.0722656	0
1952	1995	Female	1125	761	FALSE	0	5.8293873	0.0128197	0.06129	0.0021395	0.0286455
1953	1987	Male	700	1389	FALSE	0	5.7157593	0.0282159	0.0251249	0.0022125	0.0021973
1954	1988	Male	644	741	FALSE	0	4.8310547	0.0008545	0.0260878	0	0.0040283
1955	1988	Male	700	1591	FALSE	0	5.5551758	0.0665436	0.0141139	0	0.0021973
1956	1996	Male	1125	761	FALSE	0	5.8293873	0.0128197	0.06129	0.0021395	0.0286455

1957	2002	Male	1399	1599	TRUE	0.001367054	6.5456238	0.0784101	0.0892239	0.034668	0.0365677
1958	1989	Female	814	1252	FALSE	0	6.5048218	0.0185099	0.1018535	0.093668	0
1959	1987	Female	950	1406	FALSE	0	4.9038086	0.0278015	0.0487809	0	0.0117188
1960	1990	Female	1262	1405	FALSE	0	6.722168	0.0743408	0.1833597	0.2651367	0.0099792
1961	1989	Male	1262	1404	FALSE	0	6.722168	0.0743408	0.1833597	0.2651367	0.0099792
1962	1991	Female	1262	1244	FALSE	0	6.222168	0.0743408	0.0582117	0.0117188	0.0099792
1963	2000	Female	1264	1119	TRUE	0.001367054	6.4199219	0.24646	0.2611758	0.2651367	0.1074219
1964	1989	Female	1600	1406	FALSE	0	4.6733398	0.0021362	0.0325127	0	0
1965	1988	Female	1600	1406	FALSE	0	4.6733398	0.0021362	0.0325127	0	0
1966	1991	Female	1262	1405	FALSE	0	6.722168	0.0743408	0.1833597	0.2651367	0.0099792
1967	1990	Female	1262	1244	FALSE	0	6.222168	0.0743408	0.0582117	0.0117188	0.0099792
1968	1991	Male	1262	1404	FALSE	0	6.722168	0.0743408	0.1833597	0.2651367	0.0099792
1969	1990	Female	1262	1404	FALSE	0	6.722168	0.0743408	0.1833597	0.2651367	0.0099792
1970	1991	Female	1262	1268	FALSE	0	6.8408203	0.043026	0.1089625	0.0480957	0.0099792
1971	2003	Male	814	825	TRUE	0.001367054	5.9995117	0.0083008	0.038457	0.0021973	0
1972	2003	Male	814	962	TRUE	0.001367054	6.5893555	0.0758362	0.0657589	0.0273743	0
1973	1990	Female	814	1593	FALSE	0	5.6308594	0.0073242	0.0530492	0.0234375	0
1974	1990	Female	814	1253	FALSE	0	6.4515381	0.0226765	0.0624666	0.0138741	0
1975	2003	Female	1399	1599	TRUE	0.001367054	6.5456238	0.0784101	0.0892239	0.034668	0.0365677
1976	1988	Male	1260	920	FALSE	0	6.0472412	0.0361557	0.0958694	0.0351563	0.0798531
1977	1991	Female	1115	741	FALSE	0	5.1459961	0.0009155	0.037315	0	0.0376587
1978	1991	Male	1115	741	FALSE	0	5.1459961	0.0009155	0.037315	0	0.0376587
1979	1992	Male	104	1253	FALSE	0	6.1116943	0.0326271	0.0606635	0.0138741	0.0146484
1980	1990	Male	1265	1257	FALSE	0	6.6748047	0.1437531	0.2306966	0.0598145	0.1831665
1981	1991	Male	1265	1242	FALSE	0	6.4624023	0.0628357	0.1999951	0.0532227	0.1831665
1982	1990	Male	1262	1107	FALSE	0	6.4682617	0.0405273	0.0971613	0.0722656	0.0099792
1983	1991	Female	1240	1114	FALSE	0	6.4619141	0.0561218	0.1507624	0.0259399	0.0976563
1984	1991	Male	1265	1257	FALSE	0	6.6748047	0.1437531	0.2306966	0.0598145	0.1831665
1985	1992	Male	104	962	FALSE	0	6.2495117	0.0013733	0.0639557	0.0273743	0.0146484
1986	1992	Male	104	1594	FALSE	0	6.2319336	0.0013733	0.0648739	0.0238342	0.0146484
1987	1989	Male	700	1083	FALSE	0	5.9911499	0.0236154	0.0775513	0.0422974	0.0021973
1988	1993	Female	104	1117	FALSE	0	6.1435547	0.1306458	0.0485556	0.0117798	0.0146484
1989	1993	Male	104	1253	FALSE	0	6.1116943	0.0326271	0.0606635	0.0138741	0.0146484
1990	1993	Male	104	1116	FALSE	0	6.2289429	0.0807362	0.0722686	0.0277424	0.0146484
1991	1990	Male	700	1256	FALSE	0	6.1865234	0.1362	0.0436163	0.0376587	0.0021973
1992	1994	Male	104	1254	FALSE	0	5.9682617	0.0018921	0.0657511	0.0449829	0.0146484
1993	1995	Female	104	1254	FALSE	0	5.9682617	0.0018921	0.0657511	0.0449829	0.0146484
1994	1992	Female	1596	1585	FALSE	0	5.9941406	0.0414581	0.0544209	0.0030518	0.0181885
1995	1994	Male	814	825	FALSE	0	5.9995117	0.0083008	0.038457	0.0021973	0
1996	1994	Male	1115	1263	FALSE	0	6.6352539	0.0523758	0.0922035	0.0259399	0.0376587
1997	1988	Male	769	457	FALSE	0	5.9174805	0.0105286	0.0933084	0.0351563	0.055603
1998	1995	Male	1115	1117	FALSE	0	6.6704102	0.0745621	0.0555181	0.0117798	0.0376587
1999	1995	Female	1115	1114	FALSE	0	6.6352539	0.0523758	0.0922035	0.0259399	0.0376587
2000	1992	Male	1125	1580	FALSE	0	6.2757225	0.0121566	0.0749412	0.0427246	0.0286455
2001	1996	Female	814	1252	FALSE	0	6.5048218	0.0185099	0.1018535	0.093668	0
2002	1996	Male	814	1254	FALSE	0	6.3081055	0.0125122	0.0675543	0.0449829	0
2003	1996	Male	814	1253	FALSE	0	6.4515381	0.0226765	0.0624666	0.0138741	0
2004	1989	Male	769	1113	FALSE	0	6.215332	0.0077133	0.0872905	0.0020142	0.055603
2005	1998	Male	814	1253	FALSE	0	6.4515381	0.0226765	0.0624666	0.0138741	0
2006	1998	Male	814	825	FALSE	0	5.9995117	0.0083008	0.038457	0.0021973	0
2007	1993	Male	1262	1406	FALSE	0	5.6494141	0.0120049	0.0510187	0	0.0099792
2008	1989	Male	1598	1572	FALSE	0	5.3519287	0.0655823	0.0585676	0.03125	0.0251656
2009	1998	Female	1264	1227	FALSE	0	6.7053833	0.0105004	0.1926775	0.0809336	0.1074219
2010	1989	Male	769	928	FALSE	0	6.0882568	0.0342388	0.080508	0.0045166	0.055603
2011	1996	Male	909	765	FALSE	0	5.1181641	0.013916	0.0299192	0.0007324	0.0236816
2012	1992	Male	1401	1605	FALSE	0	6.7827148	0.0445786	0.1085573	0.0394897	0.0170898

2013	1995	Male	1399	1613	FALSE	0	6.6007996	0.0206208	0.0717799	0.0120049	0.0365677
2014	1990	Male	1228	1447	FALSE	0	6.5014038	0.0160871	0.1152968	0.0394897	0.0174866
2015	1996	Female	899	1582	FALSE	0	5.084684	0.0068752	0.0151855	0.0029297	0.0055404
2016	1996	Male	909	879	FALSE	0	5.1035156	0.0097656	0.029065	0	0.0236816
2017	1991	Male	1228	1447	FALSE	0	6.5014038	0.0160871	0.1152968	0.0394897	0.0174866
2018	1993	Female	1286	1576	FALSE	0	6.4097208	0.0522798	0.0516019	0.0351715	0.001301
2019	1998	Male	1440	1434	FALSE	0	7.3026346	0.0251954	0.203397	0.1437531	0.0024304
2020	1993	Male	1390	1432	FALSE	0	6.8544006	0.1700155	0.135702	0.0361557	0.0595665
2021	1996	Female	1423	1434	FALSE	0	7.3408203	0.0913391	0.2297997	0.1437531	0.0371399
2022	1989	Female	1610	898	FALSE	0	5.4624836	0.2576532	0.0338781	0.0119221	0.0119221
2023	1992	Male	971	1626	FALSE	0	4.4114826	0.0178019	0.0130384	0	0.0124893
2024	1990	Male	1627	1267	FALSE	0	6.8203125	0.0929413	0.1517733	0.0330811	0.0662231
2025	1992	Female	881	1580	FALSE	0	6.743083	0.0064318	0.0918194	0.0427246	0.040494
2026	1998	Male	1269	1428	FALSE	0	7.1929932	0.171015	0.0903351	0.0226765	0.0411091
2027	1989	Female	899	442	FALSE	0	3.5026528	0	0.0117732	0	0.0055404
2028	1997	Male	0	1613	FALSE	0	3.324707	0	0.0312055	0.0120049	
2029	1992	Male	1286	546	FALSE	0	5.8873819	0.0488493	0.0298797	0.0234375	0.001301
2030	1992	Male	1286	1576	FALSE	0	6.4097208	0.0522798	0.0516019	0.0351715	0.001301
2031	2001	Male	1269	1428	TRUE	0.001367054	7.1929932	0.171015	0.0903351	0.0226765	0.0411091
2032	1993	Male	1286	1575	FALSE	0	6.4097208	0.0522798	0.0516019	0.0351715	0.001301
2033	1993	Male	1286	660	FALSE	0	5.7194132	0.2526036	0.0125304	0.0078125	0.001301
2034	1998	Male	1407	1130	FALSE	0	6.9648438	0.0853195	0.1179688	0.0662231	0.0150452
2035	1993	Female	881	895	FALSE	0	7.0206098	0.0017229	0.0990574	0.0342388	0.040494
2036	1994	Female	1286	1284	FALSE	0	6.4935139	0.1352	0.0551757	0.0195824	0.001301
2037		Male	881	785	FALSE	0	6.8480024	0.0064511	0.0858248	0.0430012	0.040494
2038	2000	Female	1436	1421	TRUE	0.001367054	7.5312195	0.0488398	0.1337389	0.0308189	0.0120339
2039	2000	Male	1436	1603	TRUE	0.001367054	7.4070587	0.0887814	0.1755193	0.0227695	0.0120339
2040	2000	Male	1436	1397	TRUE	0.001367054	6.8373718	0.0462894	0.1373174	0.0639648	0.0120339
2041	2000	Female	1436	1611	TRUE	0.001367054	7.5109558	0.0299438	0.19152	0.0743408	0.0120339
2042	2000	Female	1436	1614	TRUE	0.001367054	6.9745789	0.0517325	0.1006908	0.0120049	0.0120339
2043	1995	Female	1286	1581	FALSE	0	4.7897257	0.0039876	0.0164367	0	0.001301
2044	1990	Female	899	896	FALSE	0	5.1935708	0.0106385	0.0175519	0.0081787	0.0055404
2045	1996	Male	1286	1284	FALSE	0	6.4935139	0.1352	0.0551757	0.0195824	0.001301
2046	2004	Male	1140	1118	TRUE	0.001367054	6.8615112	0.0544951	0.0730534	0.0277424	0.010376
2047		Male	1125	1417	FALSE	0	6.5615881	0.0067947	0.0745158	0.0009984	0.0286455
2048	1997	Male	1286	1284	FALSE	0	6.4935139	0.1352	0.0551757	0.0195824	0.001301
2049	1990	Male	899	442	FALSE	0	3.5026528	0	0.0117732	0	0.0055404
2050	2003	Male	1407	1603	TRUE	0.001367054	7.2845306	0.0654056	0.1487212	0.0227695	0.0150452
2051	2002	Male	1399	1606	TRUE	0.001367054	6.6069031	0.0279926	0.0685575	0.0008545	0.0365677
2052	2005	Female	814	1437	TRUE	0.001367054	6.729248	0.0209198	0.087898	0.0578156	0
2053	2005	Male	1414	1615	TRUE	0.001367054	7.1520996	0.092638	0.0753077	0.0013733	0.0150452
2054	2001	Female	1140	1117	TRUE	0.001367054	6.776123	0.0715218	0.0493404	0.0117798	0.010376
2055	2001	Male	1140	1118	TRUE	0.001367054	6.8615112	0.0544951	0.0730534	0.0277424	0.010376
2056	2003	Female	1140	1118	TRUE	0.001367054	6.8615112	0.0544951	0.0730534	0.0277424	0.010376
2057	2003	Male	814	1621	TRUE	0.001367054	6.6195984	0.073586	0.0821347	0.0172596	0
2058	2004	Female	1143	1625	TRUE	0.001367054	7.1245117	0.0870342	0.0558399	0.0013733	0.0083008
2059	2003	Male	1140	1114	TRUE	0.001367054	6.7409668	0.0470009	0.0860257	0.0259399	0.010376
2060	2004	Female	1143	1444	TRUE	0.001367054	7.1245117	0.0870342	0.0558399	0.0013733	0.0083008
2061	2003	Male	1143	1145	TRUE	0.001367054	7.1245117	0.0870342	0.0558399	0.0013733	0.0083008
2062	2004	Male	1140	1622	TRUE	0.001367054	7.2077332	0.0617706	0.0827841	0.0431027	0.010376
2063	2003	Female	814	1442	TRUE	0.001367054	6.6195984	0.073586	0.0821347	0.0172596	0
2064	2003	Male	1140	1623	TRUE	0.001367054	7.1474609	0.0580235	0.0931997	0.0523758	0.010376
2065	2004	Female	1414	1437	TRUE	0.001367054	7.2194824	0.0322666	0.106242	0.0578156	0.0150452
2066	2003	Female	1140	103	TRUE	0.001367054	6.4899902	0.0068436	0.0673685	0.0166016	0.010376
2067	2003	Female	1140	1144	TRUE	0.001367054	6.8813477	0.0991402	0.1038759	0.1296387	0.010376
2068	2004	Female	1140	1409	TRUE	0.001367054	6.9528809	0.0075569	0.1325344	0.1049194	0.010376

2069	1994	Male	1431	1405	FALSE	0	7.1586914	0.0753174	0.2267851	0.2651367	0.043026
2070	2005	Female	1436	1595	TRUE	0.001367054	6.8881531	0.0301547	0.2226202	0.2651367	0.0120339
2071	2005	Female	1436	1603	TRUE	0.001367054	7.4070587	0.0887814	0.1755193	0.0227695	0.0120339
2072	1991	Female	899	1590	FALSE	0	5.1925652	0.0152949	0.02355	0.0129982	0.0055404
2073	1991	Male	899	1578	FALSE	0	5.8458535	0.0031972	0.048138	0.0444565	0.0055404
2074	1992	Male	850	98	FALSE	0	5.1381836	0.003418	0.0173662	0	0.0008545
2075	1991	Female	1423	1608	FALSE	0	7.2346191	0.0803757	0.1842885	0.0628357	0.0371399
2076	1992	Female	850	103	FALSE	0	6.0756836	0.0121613	0.0575038	0.0166016	0.0008545
2077	1991	Male	899	1389	FALSE	0	5.5060097	0.0031691	0.0227842	0.0022125	0.0055404
2078	2006	Female	1436	1595	TRUE	0.001367054	6.8881531	0.0301547	0.2226202	0.2651367	0.0120339
2079	2006	Female	1436	1603	TRUE	0.001367054	7.4070587	0.0887814	0.1755193	0.0227695	0.0120339
2080	2001	Male	1286	1284	TRUE	0.001367054	6.4935139	0.1352	0.0551757	0.0195824	0.001301
2081	1992	Male	850	1263	FALSE	0	6.3266602	0.0277481	0.076161	0.0259399	0.0008545
2082	2007	Female	1436	1603	TRUE	0.001367054	7.4070587	0.0887814	0.1755193	0.0227695	0.0120339
2083	1994	Male	104	1612	FALSE	0	6.3513184	0.0652771	0.0621505	0.0125122	0.0146484
2084	1993	Male	850	959	FALSE	0	5.9814453	0.1274261	0.0252669	0.0014648	0.0008545
2085	1994	Male	850	1080	FALSE	0	5.8128662	0.0374575	0.047345	0.0240479	0.0008545
2086	1994	Male	104	1136	FALSE	0	5.6767578	0.0686035	0.0399173	0.0026855	0.0146484
2087	1992	Male	971	896	FALSE	0	5.2625569	0.0093171	0.0178406	0.0081787	0.0124893
2088	1992	Male	899	1389	FALSE	0	5.5060097	0.0031691	0.0227842	0.0022125	0.0055404
2089	1990	Male	732	1146	FALSE	0	4.703125	0.0018311	0.044548	0.0195313	0.0351563
2090	2009	Male	1436	1603	TRUE	0.001367054	7.4070587	0.0887814	0.1755193	0.0227695	0.0120339
2091	1995	Female	1115	1612	FALSE	0	6.8781738	0.1025314	0.0691131	0.0125122	0.0376587
2092	1990	Female	1624	898	FALSE	0	4.5483499	0.1279805	0.0232717	0.0119221	0
2093	2005	Female	1143	1625	TRUE	0.001367054	7.1245117	0.0870342	0.0558399	0.0013733	0.0083008
2094	1995	Female	1423	1434	FALSE	0	7.3408203	0.0913391	0.2297997	0.1437531	0.0371399
2095	1990	Female	906	879	FALSE	0	4.6149902	0.0079041	0.0128602	0	0.0059509
2096	1995	Female	1115	1617	FALSE	0	6.976532	0.0921996	0.0887415	0.0185099	0.0376587
2097	1997	Male	104	1612	FALSE	0	6.3513184	0.0652771	0.0621505	0.0125122	0.0146484
2098	1994	Female	1619	1618	FALSE	0	6.8727722	0.0955433	0.1222405	0.0611386	0.0758209
2099	1996	Female	1423	1242	FALSE	0	6.4916992	0.0403442	0.1106645	0.0532227	0.0371399
2100	1996	Male	104	979	FALSE	0	6.1970215	0.069519	0.0457592	0.0083008	0.0146484
2101	1996	Female	850	103	FALSE	0	6.0756836	0.0121613	0.0575038	0.0166016	0.0008545
2102	1997	Male	1140	1252	FALSE	0	6.7975464	0.083708	0.1008351	0.093668	0.010376
2103	1997	Female	1140	1253	FALSE	0	6.7442627	0.0454526	0.0614483	0.0138741	0.010376
2104	1997	Male	1423	1608	FALSE	0	7.2346191	0.0803757	0.1842885	0.0628357	0.0371399
2105	1994	Female	881	1580	FALSE	0	6.743083	0.0064318	0.0918194	0.0427246	0.040494
2106	1997	Male	1140	1114	FALSE	0	6.7409668	0.0470009	0.0860257	0.0259399	0.010376
2107	1992	Female	909	898	FALSE	0	5.6316324	0.0163569	0.0460041	0.0119221	0.0236816
2108	1998	Female	1264	1449	FALSE	0	6.8816223	0.005769	0.1708503	0.0055017	0.1074219
2109	1998	Female	1436	1608	FALSE	0	7.381073	0.0697352	0.1946173	0.0628357	0.0120339
2110	1993	Male	909	840	FALSE	0	5.8853293	0.0142442	0.0359066	0.0028568	0.0236816
2111	1994	Male	909	879	FALSE	0	5.1035156	0.0097656	0.029065	0	0.0236816
2112	1994	Female	909	840	FALSE	0	5.8853293	0.0142442	0.0359066	0.0028568	0.0236816
2113	1994	Female	909	898	FALSE	0	5.6316324	0.0163569	0.0460041	0.0119221	0.0236816
2114	2013	Female	1609	1136	TRUE	0.001367054	6.9522552	0.0729247	0.0823518	0.0026855	0.0540786
2115	1995	Male	909	1059	FALSE	0	5.1269531	0.0072021	0.0329713	0.0078125	0.0236816
2116	1996	Female	1630	1629	FALSE	0	6.3497902	0.1294938	0.0269425	0.0031691	0.0106385
2117	2001	Male	1636	1633	TRUE	0.001367054	7.7174358	0.031305	0.1301173	0.005769	0.0026385
2118	2004	Female	1414	1464	TRUE	0.001367054	7.3149261	0.108835	0.10265	0.0413141	0.0150452
2119	1996	Male	1431	999	FALSE	0	7.8444113	0.0204323	0.1195112	0.0064511	0.043026
2120	2001	Male	1636	1424	TRUE	0.001367054	7.3290918	0.0742708	0.0906321	0.0009984	0.0026385
2121	1992	Female	1301	660	FALSE	0	5.7689045	0.0486643	0.0221141	0.0078125	0.0035027
2122	1992	Female	1301	140	FALSE	0	6.0345295	0.0591332	0.0811351	0	0.0035027
2123	1998	Female	1269	1631	FALSE	0	6.9503784	0.0488536	0.1058679	0.0147705	0.0411091
2124	1998	Female	1040	1299	FALSE	0	5.9857178	0.0169353	0.0478167	0.0148315	0.0139313

2125	1993	Male	1301	140	FALSE	0	6.0345295	0.0591332	0.0811351	0	0.0035027
2126	1999	Female	1105	1633	FALSE	0	7.2146759	0.050958	0.1472296	0.005769	0.0438576
2127	2008	Female	1454	1282	TRUE	0.001367054	7.3128662	0.0929842	0.1147928	0.0648499	0.0158577
2128	2000	Male	1269	1299	TRUE	0.001367054	6.6381226	0.0039563	0.0825612	0.0148315	0.0411091
2129	2002	Female	1040	1091	TRUE	0.001367054	5.7413025	0.0148153	0.0428351	0.0225983	0.0139313
2130	1999	Male	1058	1006	FALSE	0	7.4529696	0.0059417	0.0751245	0.0017229	0.0142442
2131	2001	Male	1035	1130	TRUE	0.001367054	6.5342854	0.0007337	0.0892835	0.0662231	0.0106385
2132	2001	Male	1269	1653	TRUE	0.001367054	6.5263062	0.0025365	0.0701812	0.013916	0.0411091
2133	2002	Male	1035	1130	TRUE	0.001367054	6.5342854	0.0007337	0.0892835	0.0662231	0.0106385
2134	1995	Male	1125	1451	FALSE	0	6.9331221	0.044121	0.0720452	0.0064511	0.0286455
2135	2000	Female	1040	1153	TRUE	0.001367054	6.6863608	0.0070957	0.0625576	0.0064318	0.0139313
2136	1998	Male	1082	1645	FALSE	0	6.2619019	0.0333302	0.0624598	0.02005	0.01651
2137	2006	Female	1636	999	TRUE	0.001367054	7.7006259	0.044163	0.0881616	0.0064511	0.0026385
2138	2002	Female	1655	1647	TRUE	0.001367054	8.1966553	0.0734885	0.2674367	0.0753174	0.0803757
2139	2002	Female	1655	1614	TRUE	0.001367054	7.4420166	0.0468106	0.1561314	0.0120049	0.0803757
2140	2002	Male	1655	1397	TRUE	0.001367054	7.3048096	0.0806961	0.1927581	0.0639648	0.0803757
2141	2001	Male	1152	999	TRUE	0.001367054	7.3666658	0.0113038	0.0706807	0.0064511	0.0142442
2142	1997	Male	1058	785	FALSE	0	6.8141856	0.0117726	0.0675822	0.0430012	0.0142442
2143	2004	Male	1035	1130	TRUE	0.001367054	6.5342854	0.0007337	0.0892835	0.0662231	0.0106385
2144	2004	Male	1636	999	TRUE	0.001367054	7.7006259	0.044163	0.0881616	0.0064511	0.0026385
2145	1997	Male	909	1000	FALSE	0	5.4316406	0.0096436	0.0329713	0.0078125	0.0236816
2146	1997	Female	1058	1275	FALSE	0	7.1329856	0.014887	0.0653087	0.0129225	0.0142442
2147	1997	Female	1125	1634	FALSE	0	6.9331221	0.044121	0.0720452	0.0064511	0.0286455
2148	1997	Male	1058	0	FALSE	0	3.4426646	0	0.0248196		0.0142442
2149	2002	Male	1152	1006	TRUE	0.001367054	7.4529696	0.0059417	0.0751245	0.0017229	0.0142442
2150	2005	Male	1035	1130	TRUE	0.001367054	6.5342854	0.0007337	0.0892835	0.0662231	0.0106385
2151	1998	Male	1023	1634	FALSE	0	6.7758078	0.0084453	0.0669069	0.0064511	0.0005035
2152	2007	Female	1636	999	TRUE	0.001367054	7.7006259	0.044163	0.0881616	0.0064511	0.0026385
2153	1998	Male	1023	1000	FALSE	0	5.3830566	0.0083618	0.024952	0.0078125	0.0005035
2154	2004	Female	1297	1412	TRUE	0.001367054	7.250946	0.0773807	0.0867743	0.0150452	0.0266709
2155	2004	Male	1143	1298	TRUE	0.001367054	7.3878174	0.1481266	0.0818859	0.0715218	0.0083008
2156	2005	Female	1297	1623	TRUE	0.001367054	7.5412292	0.0979781	0.1139622	0.0523758	0.0266709
2157	2007	Male	1297	1443	TRUE	0.001367054	7.4493713	0.1012992	0.0859505	0.0226765	0.0266709
2158	2007	Male	1297	1412	TRUE	0.001367054	7.250946	0.0773807	0.0867743	0.0150452	0.0266709
2159	2008	Female	1297	1145	TRUE	0.001367054	7.3483582	0.0728285	0.0767078	0.0013733	0.0266709
2160	2009	Male	1297	1623	TRUE	0.001367054	7.5412292	0.0979781	0.1139622	0.0523758	0.0266709
2161	2010	Female	1297	1642	TRUE	0.001367054	7.7850037	0.0760445	0.1358131	0.0588112	0.0266709
2162	2011	Male	1297	1616	TRUE	0.001367054	7.5202332	0.0936841	0.1053772	0.07201	0.0266709
2163	2012	Male	1297	1642	TRUE	0.001367054	7.7850037	0.0760445	0.1358131	0.0588112	0.0266709
2164	2013	Female	1297	1616	TRUE	0.001367054	7.5202332	0.0936841	0.1053772	0.07201	0.0266709
2165	2013	Female	1297	1453	TRUE	0.001367054	7.7505798	0.0910081	0.1405855	0.10779	0.0266709
2166	2013	Female	1297	1642	TRUE	0.001367054	7.7850037	0.0760445	0.1358131	0.0588112	0.0266709
2167	2001	Male	1040	1282	TRUE	0.001367054	6.3690186	0.0050654	0.0860087	0.0648499	0.0139313
2168	2001	Female	1040	959	TRUE	0.001367054	5.8807373	0.0107417	0.0255342	0.0014648	0.0139313
2169	2001	Male	1040	1462	TRUE	0.001367054	6.745575	0.0063049	0.075511	0.0544951	0.0139313
2170	2001	Male	1040	1617	TRUE	0.001367054	6.5672302	0.0056243	0.0729663	0.0185099	0.0139313
2171	2003	Female	1040	1651	TRUE	0.001367054	6.6152344	0.0097357	0.0796485	0.0699615	0.0139313
2172	2002	Female	1040	1246	TRUE	0.001367054	5.552124	0.000823	0.0393239	0.0361328	0.0139313
2173	2002	Female	1040	1437	TRUE	0.001367054	6.506958	0.004544	0.0772821	0.0578156	0.0139313
2174	2002	Female	1040	1464	TRUE	0.001367054	6.6024017	0.0069227	0.0736901	0.0413141	0.0139313
2175	2002	Female	1040	825	TRUE	0.001367054	5.7772217	0.0130248	0.0278411	0.0021973	0.0139313
2176	2002	Female	1040	1443	TRUE	0.001367054	6.5405884	0.0056546	0.0555906	0.0226765	0.0139313
2177	2003	Female	1040	1621	TRUE	0.001367054	6.3973083	0.0086685	0.0715188	0.0172596	0.0139313
2178	2002	Female	1040	979	TRUE	0.001367054	6.3145752	0.0077856	0.0369465	0.0083008	0.0139313
2179	2003	Male	1143	1644	TRUE	0.001367054	7.2112732	0.1084164	0.0931463	0.0806446	0.0083008
2180	2004	Female	1297	1412	TRUE	0.001367054	7.250946	0.0773807	0.0867743	0.0150452	0.0266709

2181	2006	Female	1035	1130	TRUE	0.001367054	6.5342854	0.0007337	0.0892835	0.0662231	0.0106385
2182	1999	Male	1023	1638	FALSE	0	6.6326007	0.0120923	0.0614478	0.0067947	0.0005035
2183	1999	Male	1023	1424	FALSE	0	6.4042738	0.0075471	0.0693774	0.0009984	0.0005035
2184	1999	Male	1023	1637	FALSE	0	6.1659546	0.0145235	0.0631485	0.035728	0.0005035
2185	1999	Female	1058	1154	FALSE	0	7.4678926	0.0056925	0.0858251	0.0026394	0.0142442
2186	2004	Male	1636	1633	TRUE	0.001367054	7.7174358	0.031305	0.1301173	0.005769	0.0026385
2187	1991	Male	814	1004	FALSE	0	5.6035156	0.0043945	0.0416327	0.0078125	0
2188	1991	Male	814	1290	FALSE	0	5.972168	0.0053711	0.0351585	0.003418	0
2189	2000	Female	1023	785	TRUE	0.001367054	6.2233276	0.0052874	0.0638083	0.0430012	0.0005035
2190	2016	Male	1454	1288	TRUE	0.001367054	6.817749	0.0150876	0.0642208	0.013916	0.0158577
2191	2000	Male	1027	1025	TRUE	0.001367054	6.1049703	0.0209387	0.0393567	0.0163569	0
2192	2000	Male	1027	1638	TRUE	0.001367054	6.5699481	0.0084822	0.0489545	0.0067947	0
2193	2002	Male	1269	1288	TRUE	0.001367054	6.5263062	0.0025365	0.0701812	0.013916	0.0411091
2194	2001	Male	1027	1637	TRUE	0.001367054	6.103302	0.008695	0.0506552	0.035728	0
2195	1992	Male	814	1290	FALSE	0	5.972168	0.0053711	0.0351585	0.003418	0
2196	1992	Male	850	1290	FALSE	0	5.8505859	0.1273041	0.0242754	0.003418	0.0008545
2197	2006	Male	1636	1637	TRUE	0.001367054	7.0907726	0.0155916	0.0844032	0.035728	0.0026385
2198	1993	Female	971	1654	FALSE	0	3.3216389	0	0.0120619	0	0.0124893
2199	2008	Male	1636	1637	TRUE	0.001367054	7.0907726	0.0155916	0.0844032	0.035728	0.0026385
2200	2003	Female	1269	1288	TRUE	0.001367054	6.5263062	0.0025365	0.0701812	0.013916	0.0411091
2201	2009	Female	1636	1637	TRUE	0.001367054	7.0907726	0.0155916	0.0844032	0.035728	0.0026385
2202	1995	Male	1040	1618	FALSE	0	6.4412537	0.0045743	0.0879254	0.0611386	0.0139313
2203	1996	Female	1459	1083	FALSE	0	6.5910034	0.0314949	0.0935076	0.0422974	0.0016708
2204	1992	Male	909	1059	FALSE	0	5.1269531	0.0072021	0.0329713	0.0078125	0.0236816
2205	1998	Female	1264	1646	FALSE	0	6.2679443	0.0005188	0.126311	0.0004101	0.1074219
2206	1995	Female	769	999	FALSE	0	7.2086692	0.0115278	0.1065157	0.0064511	0.055603
2207	1998	Female	1635	1242	FALSE	0	6.7690753	0.0295628	0.0919101	0.0532227	0.0067947
2208	1994	Male	909	1000	FALSE	0	5.4316406	0.0096436	0.0329713	0.0078125	0.0236816
2209	2004	Female	1023	1000	TRUE	0.001367054	5.3830566	0.0083618	0.024952	0.0078125	0.0005035
2210	2002	Female	1652	1608	TRUE	0.001367054	7.5502777	0.1822293	0.2090853	0.0628357	0.053371
2211	2002	Female	1652	1242	TRUE	0.001367054	6.8073578	0.2899911	0.1354613	0.0532227	0.053371
2212	2002	Male	1040	1279	TRUE	0.001367054	6.2400126	0.0037509	0.0526716	0.0025846	0.0139313
2213	2005	Male	1304	1059	TRUE	0.001367054	5.4423828	0.0067749	0.0250547	0.0078125	0.0096436
2214	1995	Male	909	1322	FALSE	0	5.7653809	0.0198135	0.0437495	0.0099487	0.0236816
2215	2006	Female	1304	1324	TRUE	0.001367054	6.6376038	0.0071728	0.0599647	0.0005069	0.0096436
2216	1980	Male	1205	1194	FALSE	0	7.22993	0.0277218	0.0768753	0.0102167	0.0248988
2217	2006	Male	1304	1663	TRUE	0.001367054	7.3991532	0.0729231	0.0617413	0.0113038	0.0096436
2218	2006	Female	1304	1059	TRUE	0.001367054	5.4423828	0.0067749	0.0250547	0.0078125	0.0096436
2219	2001	Male	1058	1664	TRUE	0.001367054	7.4598391	0.0118469	0.0861971	0.0389626	0.0142442
2220	2013	Male	1469	1457	TRUE	0.001367054	7.2562092	0.0935644	0.1681818	0.1352	0.0632836
2221	2005	Male	1297	1671	TRUE	0.001367054	7.5750427	0.0394606	0.0838485	0.0076851	0.0266709
2222	2002	Female	1666	1664	TRUE	0.001367054	8.0147404	0.0422706	0.1004286	0.0389626	0.0053417
2223	2006	Female	1323	1139	TRUE	0.001367054	6.7804748	0.2523912	0.0576966	0.0021238	0.0037206
2224	2011	Male	1205	1194	TRUE	0.001367054	7.22993	0.0277218	0.0768753	0.0102167	0.0248988
2225	1991	Male	971	1465	FALSE	0	5.5906208	0.0057642	0.0223326	0.0007019	0.0124893
2226	2006	Female	1318	1303	TRUE	0.001367054	6.5200422	0.0147246	0.0424207	0.0005951	0.0039026
2227	2006	Female	1318	1279	TRUE	0.001367054	6.7299034	0.0105213	0.0626397	0.0025846	0.0039026
2228	2004	Male	1197	1677	TRUE	0.001367054	3.9104834	0	0.0276134	0	0.0181582
2229	1996	Female	1480	1614	FALSE	0	6.3881836	0.0026054	0.0511735	0.0120049	0.0072021
2230	1996	Male	1480	1595	FALSE	0	6.3017578	0	0.1731029	0.2651367	0.0072021
2231	2009	Male	1297	1473	TRUE	0.001367054	7.139801	0.0149233	0.0774448	0.0063114	0.0266709
2232	2008	Male	1318	1482	TRUE	0.001367054	6.3681868	0.0132225	0.0436633	0.0072021	0.0039026
2233	2009	Female	1314	1444	TRUE	0.001367054	7.3314209	0.089609	0.0713271	0.0013733	0.0082101
2234	2012	Male	1315	1668	TRUE	0.001367054	7.7740326	0.0575985	0.1227457	0.0750617	0.0014963
2235	2007	Male	1304	1084	TRUE	0.001367054	6.2445679	0.0013452	0.0845858	0.0422974	0.0096436
2236	2012	Female	1205	1473	TRUE	0.001367054	7.0146632	0.0154457	0.075468	0.0063114	0.0248988

2237	1992	Male	1286	1465	FALSE	0	5.9493326	0.0158234	0.0188949	0.0007019	0.001301
2238	2007	Male	1318	1676	TRUE	0.001367054	7.1002119	0.0113849	0.0847241	0.0314949	0.0039026
2239	2007	Male	1314	1441	TRUE	0.001367054	7.1907959	0.0860699	0.0724659	0.0018921	0.0082101
2240	2012	Female	1660	1633	TRUE	0.001367054	7.0042877	0.0116423	0.1077848	0.005769	0.0072021
2241	1989	Female	644	1656	FALSE	0	5.5615234	0.0301819	0.0368481	0.0009766	0.0040283
2242	2008	Female	1318	1083	TRUE	0.001367054	6.3334577	0.0085362	0.0871327	0.0422974	0.0039026
2243	2010	Female	1467	1482	TRUE	0.001367054	6.8825378	0.072757	0.0630634	0.0072021	0.0039563
2244	2002	Male	1040	1657	TRUE	0.001367054	5.8782959	0.010746	0.0336953	0.0072021	0.0139313
2245	2011	Female	1205	1144	TRUE	0.001367054	7.1499782	0.0072926	0.1226615	0.1296387	0.0248988
2246	2011	Male	1467	1482	TRUE	0.001367054	6.8825378	0.072757	0.0630634	0.0072021	0.0039563
2247	2011	Male	1609	1673	TRUE	0.001367054	7.4131317	0.0241708	0.0829752	0.0107417	0.0540786
2248	2009	Female	1313	1159	TRUE	0.001367054	7.2816772	0.0843787	0.1061966	0.069519	0.006644
2249	2005	Male	1206	1634	TRUE	0.001367054	7.4764863	0.0132137	0.0755968	0.0064511	0.0209387
2250	2010	Male	1205	1464	TRUE	0.001367054	7.3860469	0.0105538	0.1020732	0.0413141	0.0248988
2251	2001	Male	1667	1663	TRUE	0.001367054	7.8854698	0.0260723	0.0787933	0.0113038	0.0075471
2252	1988	Female	644	1168	FALSE	0	5.5732422	0.0454102	0.0346022	0	0.0040283
2253	2002	Female	1678	1433	TRUE	0.001367054	7.1071777	0.0005379	0.1591497	0.0628357	0.0166931
2254	2002	Male	1197	999	TRUE	0.001367054	7.3344846	0.0161406	0.0734745	0.0064511	0.0181582
2255	1989	Male	644	1659	FALSE	0	5.5458984	0.02005	0.0265684	0.0009766	0.0040283
2256	1998	Female	1286	1466	FALSE	0	6.9518533	0.0907656	0.0651171	0.0515657	0.001301
2257	2007	Male	1304	1658	TRUE	0.001367054	6.5864716	0.0160592	0.0496563	0.0148153	0.0096436
2258	2003	Female	1197	1661	TRUE	0.001367054	7.5675864	0.0147419	0.0694856	0.010897	0.0181582
2259	2003	Female	1197	1006	TRUE	0.001367054	7.4207883	0.0130473	0.0779182	0.0017229	0.0181582
2260	2007	Female	1315	1623	TRUE	0.001367054	7.430603	0.0847889	0.1087801	0.0523758	0.0014963
2261	2007	Female	1315	1642	TRUE	0.001367054	7.6743774	0.0586895	0.1306311	0.0588112	0.0014963
2262	2009	Female	1297	1673	TRUE	0.001367054	7.1639709	0.0599996	0.062088	0.0107417	0.0266709
2263	2009	Female	1314	1641	TRUE	0.001367054	7.7124023	0.077316	0.1132782	0.0787144	0.0082101
2264	2009	Female	1314	1453	TRUE	0.001367054	7.7336426	0.0929404	0.1352049	0.10779	0.0082101
2265	2009	Female	1314	1668	TRUE	0.001367054	7.8677216	0.1570108	0.1225471	0.0750617	0.0082101
2266	2012	Female	1297	1672	TRUE	0.001367054	7.5963898	0.0878723	0.0847571	0.0063049	0.0266709
2267	2015	Male	1315	1669	TRUE	0.001367054	7.8376617	0.0713648	0.1281766	0.1012992	0.0014963
2268	2015	Male	1315	1642	TRUE	0.001367054	7.6743774	0.0586895	0.1306311	0.0588112	0.0014963
2269	2001	Female	1040	1210	TRUE	0.001367054	6.0557861	0.0129268	0.0315276	0.0050049	0.0139313
2270	1999	Male	1187	785	FALSE	0	6.6579784	0.0110904	0.0654487	0.0430012	0.0071462
2271	1999	Male	1187	1451	FALSE	0	7.2104586	0.0113514	0.0685473	0.0064511	0.0071462
2272	2005	Male	1197	1176	TRUE	0.001367054	6.6512814	0.0131473	0.0468123	0.0098812	0.0181582
2273	1991	Female	1115	1168	FALSE	0	5.8881836	0.0407715	0.0458294	0	0.0376587
2274	2000	Female	1187	898	TRUE	0.001367054	6.0176991	0.0127998	0.0396252	0.0119221	0.0071462
2275	2000	Male	1187	1451	TRUE	0.001367054	7.2104586	0.0113514	0.0685473	0.0064511	0.0071462
2276	1991	Female	104	1659	FALSE	0	5.3339844	0.0064087	0.0308331	0.0009766	0.0146484
2277	2005	Male	1318	1084	TRUE	0.001367054	6.3334577	0.0085362	0.0871327	0.0422974	0.0039026
2278	2014	Female	1468	1308	TRUE	0.001367054	6.6260173	0.0096326	0.0568033	0.0064511	0.0063477
2279	2010	Male	1205	1657	TRUE	0.001367054	6.6619411	0.0164004	0.0620783	0.0072021	0.0248988
2280	2011	Female	1675	1665	TRUE	0.001367054	6.8761022	0.0132343	0.0519618	0.0178537	0.0083618
2281	2001	Male	1187	1000	TRUE	0.001367054	5.8177074	0.0089393	0.0265924	0.0078125	0.0071462
2282	2001	Male	1286	1662	TRUE	0.001367054	6.9376931	0.1013954	0.0778657	0.0386327	0.001301
2283	2013	Male	1205	1464	TRUE	0.001367054	7.3860469	0.0105538	0.1020732	0.0413141	0.0248988
2284	2008	Female	1197	1176	TRUE	0.001367054	6.6512814	0.0131473	0.0468123	0.0098812	0.0181582
2285	2007	Male	1297	1674	TRUE	0.001367054	7.3946838	0.0436962	0.0743839	0.0044289	0.0266709
2286	2002	Female	1187	1471	TRUE	0.001367054	7.3529502	0.0120309	0.0622979	0.014887	0.0071462
2287	2001	Male	1304	1632	TRUE	0.001367054	7.1430206	0.0017365	0.1624715	0.1700155	0.0096436
2288	1994	Female	814	1659	FALSE	0	5.6738281	0.0100708	0.0326363	0.0009766	0
2289	2004	Male	1469	1457	TRUE	0.001367054	7.2562092	0.0935644	0.1681818	0.1352	0.0632836
2290	2004	Female	1469	1640	TRUE	0.001367054	7.2562092	0.0935644	0.1681818	0.1352	0.0632836
2291	2008	Female	1469	1662	TRUE	0.001367054	7.2667947	0.1546785	0.1459654	0.0386327	0.0632836
2292	2005	Male	1469	1466	TRUE	0.001367054	7.2809549	0.1313435	0.1332167	0.0515657	0.0632836

2293	2005 Male	1469	1458	TRUE	0.001367054	7.2562092	0.0935644	0.1681818	0.1352	0.0632836
2294	2004 Female	1197	1661	TRUE	0.001367054	7.5675864	0.0147419	0.0694856	0.010897	0.0181582
2295	2006 Female	1469	1639	TRUE	0.001367054	7.0019714	0.1560192	0.1202182	0.0166271	0.0632836
2296	2006 Female	1469	1457	TRUE	0.001367054	7.2562092	0.0935644	0.1681818	0.1352	0.0632836
2297	2012 Female	1205	1483	TRUE	0.001367054	4.0984645	0	0.0421103	0	0.0248988
2298	2010 Female	1469	1639	TRUE	0.001367054	7.0019714	0.1560192	0.1202182	0.0166271	0.0632836
2299	2007 Female	1469	1640	TRUE	0.001367054	7.2562092	0.0935644	0.1681818	0.1352	0.0632836
2300	2007 Male	1469	1662	TRUE	0.001367054	7.2667947	0.1546785	0.1459654	0.0386327	0.0632836
2301	2007 Male	1469	1466	TRUE	0.001367054	7.2809549	0.1313435	0.1332167	0.0515657	0.0632836
2302	2007 Female	1469	1457	TRUE	0.001367054	7.2562092	0.0935644	0.1681818	0.1352	0.0632836
2303	2013 Male	1205	1464	TRUE	0.001367054	7.3860469	0.0105538	0.1020732	0.0413141	0.0248988
2304	1999 Male	1187	1139	FALSE	0	6.5351674	0.0093552	0.0553922	0.0021238	0.0071462
2305	2004 Male	1197	1657	TRUE	0.001367054	6.4739599	0.0148865	0.0475814	0.0072021	0.0181582
2306	1999 Male	1304	1432	FALSE	0	6.7394409	0.0020986	0.0854279	0.0361557	0.0096436
2307	1998 Female	1678	1613	FALSE	0	6.7006836	0.0020409	0.0652232	0.0120049	0.0166931
2308	2008 Male	1469	1640	TRUE	0.001367054	7.2562092	0.0935644	0.1681818	0.1352	0.0632836
2309	2013 Male	1205	1464	TRUE	0.001367054	7.3860469	0.0105538	0.1020732	0.0413141	0.0248988
2310	1980 Female	1182	1306	FALSE	0	2.3339844	0	0	0	0
2311	2010 Female	1469	1457	TRUE	0.001367054	7.2562092	0.0935644	0.1681818	0.1352	0.0632836
2312	1994 Female	909	1166	FALSE	0	5.4746094	0.0127563	0.0329637	0.0039063	0.0236816
2313	2011 Male	1323	1309	TRUE	0.001367054	7.2989074	0.0134878	0.0699664	0.0007337	0.0037206
2314	1989 Male	125	1465	FALSE	0	5.3080444	0.0258827	0.0141769	0.0007019	0.0078125
2315	2006 Female	1481	1461	TRUE	0.001367054	7.7001419	0.0238687	0.1268524	0.0052921	0.000665
2316	2006 Male	1481	1449	TRUE	0.001367054	7.4854279	0.014204	0.1289396	0.0055017	0.000665
2317	2014 Male	1468	1309	TRUE	0.001367054	6.4546427	0.0059101	0.0577927	0.0007337	0.0063477
2318	2006 Female	1304	1700	TRUE	0.001367054	6.7307964	0.0320621	0.0672786	0.0663502	0.0096436
2319	1992 Female	1331	1413	FALSE	0	6.1756654	0.0007163	0.0604597	0.0258789	0.0122211
2320	1993 Female	1331	923	FALSE	0	6.0076967	0.0069533	0.0313064	0.0019531	0.0122211
2321	2009 Male	1205	1493	TRUE	0.001367054	7.2961755	0.0180692	0.063191	0.0079816	0.0248988
2322	1994 Female	1331	1403	FALSE	0	6.5457216	0.0025793	0.0684763	0.0110779	0.0122211
2323	2008 Male	1205	1498	TRUE	0.001367054	7.6194389	0.0230972	0.0788172	0.0117696	0.0248988
2324	1992 Male	1331	1396	FALSE	0	5.8397279	0.0001961	0.0275448	0.0175781	0.0122211
2325	1989 Male	1377	1416	FALSE	0	5.7924805	0.0006409	0.0698498	0.0345459	0.0234375
2326	2005 Male	1693	1637	TRUE	0.001367054	7.2336102	0.0134727	0.081499	0.035728	0.008369
2327	2017 Male	1362	1701	TRUE	0.001367054	7.6948305	0.0077165	0.0715379	0.0134878	0.0277774
2328	2010 Male	1691	1687	TRUE	0.001367054	7.8649533	0.1348266	0.0652953	0.0161406	0.0079816
2329	1992 Female	1331	1428	FALSE	0	6.877997	0.0035324	0.0567814	0.0226765	0.0122211
2330	2016 Female	1379	1194	TRUE	0.001367054	7.1282745	0.0153851	0.0661054	0.0102167	0.0171319
2331	2007 Male	1197	1698	TRUE	0.001367054	7.3540325	0.0192536	0.069077	0.0104639	0.0181582
2332	2016 Male	1504	1701	TRUE	0.001367054	7.2456204	0.0037583	0.0515061	0.0134878	0.0016036
2333	2015 Female	1467	1686	TRUE	0.001367054	7.0803031	0.0090254	0.0571965	0.0037445	0.0039563
2334	1993 Male	1331	1130	FALSE	0	6.5897279	0.0005453	0.0901998	0.0662231	0.0122211
2335	2002 Female	1494	1638	TRUE	0.001367054	7.9366367	0.0500997	0.0860145	0.0067947	0.0082214
2336	2007 Male	1680	1657	TRUE	0.001367054	6.7737863	0.0449769	0.0473262	0.0072021	0.0119151
2337	2008 Male	1344	1698	TRUE	0.001367054	8.0845616	0.0252079	0.0869864	0.0104639	0.0158104
2338	1992 Female	1331	966	FALSE	0	6.1756654	0.0007163	0.0604597	0.0258789	0.0122211
2339	2008 Female	1344	1337	TRUE	0.001367054	8.3186258	0.0303442	0.0942356	0.0402919	0.0158104
2340	2005 Male	1197	1358	TRUE	0.001367054	7.1207931	0.0096486	0.0549716	0.0119151	0.0181582
2341	2019 Female	1708	1324	TRUE	0.001367054	7.4319074	0.1346639	0.0848149	0.0005069	0.0120457
2342	2008 Female	1344	1293	TRUE	0.001367054	8.2032683	0.0169532	0.1145298	0.0870342	0.0158104
2343	2010 Male	1330	1324	TRUE	0.001367054	7.5942205	0.0196625	0.1467377	0.0005069	0.0334404
2344	2012 Female	1297	1689	TRUE	0.001367054	7.5966187	0.0380589	0.0925258	0.04261	0.0266709
2345	2008 Female	1492	1118	TRUE	0.001367054	7.6726897	0.0201073	0.0952515	0.0277424	0.0158104
2346	2008 Female	1344	1144	TRUE	0.001367054	7.6925261	0.0119098	0.1260739	0.1296387	0.0158104
2347	1992 Female	971	1711	FALSE	0	5.9756611	0.0035331	0.0319082	0.0258827	0.0124893
2348	2017 Female	1430	1327	TRUE	0.001367054	7.1641265	0.0173489	0.1334091	0.0334404	0.0111939

2349	2003	Female	1494	1059	TRUE	0.001367054	6.3824052	0.0045059	0.0495187	0.0078125	0.0082214
2350	2014	Male	1379	1320	TRUE	0.001367054	3.996809	0	0.0313404	0	0.0171319
2351	2003	Male	1494	785	TRUE	0.001367054	7.5273637	0.0194428	0.088375	0.0430012	0.0082214
2352	2004	Male	1494	1638	TRUE	0.001367054	7.9366367	0.0500997	0.0860145	0.0067947	0.0082214
2353	2010	Female	1344	1144	TRUE	0.001367054	7.6925261	0.0119098	0.1260739	0.1296387	0.0158104
2354	2011	Female	1197	1695	TRUE	0.001367054	7.2522147	0.0134103	0.0721782	0.0471884	0.0181582
2355	1992	Female	1125	1366	FALSE	0	5.1761131	0.0008886	0.0574341	0.0625	0.0286455
2356	2010	Female	1467	1333	TRUE	0.001367054	7.056366	0.071789	0.0657452	0.0127563	0.0039563
2357	2010	Female	1344	1604	TRUE	0.001367054	7.7022612	0.0236711	0.0998665	0.0185785	0.0158104
2358	2011	Female	1205	1498	TRUE	0.001367054	7.6194389	0.0230972	0.0788172	0.0117696	0.0248988
2359	2014	Female	1379	1488	TRUE	0.001367054	6.0741405	0.0072263	0.0507926	0	0.0171319
2360	2017	Female	1508	1649	TRUE	0.001367054	7.7547834	0.0108981	0.1577676	0.0298486	0.0120457
2361	2009	Female	1369	1441	TRUE	0.001367054	7.2006795	0.0140698	0.060783	0.0018921	0.0100948
2362	2010	Male	1205	1374	TRUE	0.001367054	7.6087077	0.0124522	0.0983944	0.0093955	0.0248988
2363	2010	Female	1496	1412	TRUE	0.001367054	7.3977051	0.0868206	0.0811423	0.0150452	0.037426
2364	2017	Male	1507	1488	TRUE	0.001367054	6.2201868	0.0051007	0.0460465	0	0.0065747
2365	2009	Male	1205	1485	TRUE	0.001367054	7.7890555	0.0177038	0.0813878	0.0104691	0.0248988
2366	2011	Male	1328	1279	TRUE	0.001367054	7.3201896	0.0070872	0.0746545	0.0025846	0.0135524
2367	2014	Female	1344	1293	TRUE	0.001367054	8.2032683	0.0169532	0.1145298	0.0870342	0.0158104
2368	2013	Female	1684	1690	TRUE	0.001367054	6.4571912	0.0052174	0.0749503	0.0672188	0
2369	1997	Female	1511	1424	FALSE	0	6.9351576	0.0051624	0.0796798	0.0009984	0.0198135
2370	2003	Female	1510	1333	TRUE	0.001367054	6.8949071	0.0078161	0.0744836	0.0127563	0.0191974
2371	1998	Male	1058	1366	FALSE	0	5.6096568	0.0009323	0.0560696	0.0625	0.0142442
2372	2003	Female	1510	1482	TRUE	0.001367054	6.721079	0.0061956	0.0718018	0.0072021	0.0191974
2373	2011	Male	1205	1485	TRUE	0.001367054	7.7890555	0.0177038	0.0813878	0.0104691	0.0248988
2374	2003	Female	1510	1166	TRUE	0.001367054	6.2318212	0.0071499	0.0557324	0.0039063	0.0191974
2375	2012	Female	1297	1345	TRUE	0.001367054	7.3626404	0.1348806	0.0676436	0.005188	0.0266709
2376	2013	Female	1297	1345	TRUE	0.001367054	7.3626404	0.1348806	0.0676436	0.005188	0.0266709
2377	2014	Male	1297	1345	TRUE	0.001367054	7.3626404	0.1348806	0.0676436	0.005188	0.0266709
2378	2003	Female	1140	1373	TRUE	0.001367054	6.6804199	0.0419922	0.0518571	0.0170898	0.010376
2379	2004	Female	1510	1333	TRUE	0.001367054	6.8949071	0.0078161	0.0744836	0.0127563	0.0191974
2380	2012	Male	1507	1485	TRUE	0.001367054	7.8334462	0.0140739	0.0658717	0.0104691	0.0065747
2381	2004	Male	1510	1482	TRUE	0.001367054	6.721079	0.0061956	0.0718018	0.0072021	0.0191974
2382	2004	Female	1197	1336	TRUE	0.001367054	6.2972021	0.0204043	0.0536143	0.0083008	0.0181582
2383	1999	Male	844	1340	FALSE	0	5.9517419	0.0114068	0.0408762	0.0039301	0.0220947
2384	1990	Male	769	1697	FALSE	0	4.9516602	0.0009766	0.0606546	0	0.055603
2385	2012	Male	1507	1358	TRUE	0.001367054	7.353165	0.009864	0.0539525	0.0119151	0.0065747
2386	1999	Male	1023	1366	FALSE	0	5.0187988	0.0017319	0.0522958	0.0625	0.0005035
2387	1989	Female	1377	1267	FALSE	0	5.9160156	0.0011597	0.089164	0.0330811	0.0234375
2388	2009	Male	1379	1703	TRUE	0.001367054	7.0629186	0.0243075	0.0666024	0.0162979	0.0171319
2389	2010	Male	1379	1650	TRUE	0.001367054	7.0556114	0.0292258	0.0501548	0.0120383	0.0171319
2390	2009	Female	1379	1471	TRUE	0.001367054	7.5633018	0.0168517	0.0709522	0.014887	0.0171319
2391	1995	Male	1401	1685	FALSE	0	6.1290346	0.0017213	0.0487869	0.0061106	0.0170898
2392	2013	Female	1468	1485	TRUE	0.001367054	6.878091	0.0151992	0.0520943	0.0104691	0.0063477
2393	2005	Male	1510	1333	TRUE	0.001367054	6.8949071	0.0078161	0.0744836	0.0127563	0.0191974
2394	2005	Female	1436	1682	TRUE	0.001367054	6.7643738	0.0444331	0.0900327	0.0003471	0.0120339
2395	2000	Female	1027	1340	TRUE	0.001367054	5.8063257	0.0102059	0.0202963	0.0039301	0
2396	2013	Male	1507	1308	TRUE	0.001367054	7.5813726	0.0094172	0.0705807	0.0064511	0.0065747
2397	2006	Male	1197	1347	TRUE	0.001367054	7.1741111	0.0133225	0.0532921	0.0098865	0.0181582
2398	2005	Male	1510	1482	TRUE	0.001367054	6.721079	0.0061956	0.0718018	0.0072021	0.0191974
2399	2010	Male	1712	1679	TRUE	0.001367054	8.0345116	0.0907114	0.1284213	0.0101728	0.0441696
2400	2006	Female	1436	1682	TRUE	0.001367054	6.7643738	0.0444331	0.0900327	0.0003471	0.0120339
2401	2007	Female	1197	1336	TRUE	0.001367054	6.2972021	0.0204043	0.0536143	0.0083008	0.0181582
2402	2011	Male	1496	1412	TRUE	0.001367054	7.3977051	0.0868206	0.0811423	0.0150452	0.037426
2403	2006	Male	1510	1333	TRUE	0.001367054	6.8949071	0.0078161	0.0744836	0.0127563	0.0191974
2404	2010	Female	1496	1373	TRUE	0.001367054	7.2209473	0.074173	0.0669876	0.0170898	0.037426

2405	2001 Male	1494	1638	TRUE	0.001367054	7.9366367	0.0500997	0.0860145	0.0067947	0.0082214
2406	2008 Male	1291	1347	TRUE	0.001367054	7.2062924	0.0292342	0.0504983	0.0098865	0.0142442
2407	2007 Female	1436	1682	TRUE	0.001367054	6.7643738	0.0444331	0.0900327	0.0003471	0.0120339
2408	2018 Male	1507	1488	TRUE	0.001367054	6.2201868	0.0051007	0.0460465	0	0.0065747
2409	2003 Female	1023	1340	TRUE	0.001367054	5.8689783	0.0134497	0.0327896	0.0039301	0.0005035
2410	2008 Female	1636	1687	TRUE	0.001367054	7.943867	0.0343838	0.086515	0.0161406	0.0026385
2411	2002 Male	1494	1664	TRUE	0.001367054	8.1730172	0.1407443	0.1069899	0.0389626	0.0082214
2412	2018 Female	1708	1485	TRUE	0.001367054	8.200715	0.021956	0.0852761	0.0104691	0.0120457
2413	2005 Female	1206	1694	TRUE	0.001367054	7.0987875	0.0326043	0.0552324	0.0111995	0.0209387
2414	2014 Male	1684	1658	TRUE	0.001367054	5.9198835	0.0102408	0.049563	0.0148153	0
2415	2018 Male	1507	1650	TRUE	0.001367054	7.2016576	0.0216584	0.0454087	0.0120383	0.0065747
2416	2008 Male	1315	1373	TRUE	0.001367054	6.963562	0.0805221	0.0674375	0.0170898	0.0014963
2417	2006 Female	1469	1696	TRUE	0.001367054	7.4853789	0.0808701	0.1517099	0.0907656	0.0632836
2418	2006 Male	1469	1692	TRUE	0.001367054	7.4853789	0.0808701	0.1517099	0.0907656	0.0632836
2419	2009 Female	1636	1687	TRUE	0.001367054	7.943867	0.0343838	0.086515	0.0161406	0.0026385
2420	2007 Male	1503	1025	TRUE	0.001367054	7.1639396	0.0493141	0.0610908	0.0163569	0.0133812
2421	2011 Male	1712	1679	TRUE	0.001367054	8.0345116	0.0907114	0.1284213	0.0101728	0.0441696
2422	2007 Male	1205	1703	TRUE	0.001367054	7.1645741	0.0748856	0.0773723	0.0162979	0.0248988
2423	2007 Male	1469	1696	TRUE	0.001367054	7.4853789	0.0808701	0.1517099	0.0907656	0.0632836
2424	1998 Male	814	1699	FALSE	0	6.3269043	0.0115356	0.0509615	0.0241699	0
2425	2018 Female	1688	1704	TRUE	0.001367054	7.7050426	0.0307801	0.0659547	0.0154457	0.0079816
2426	2009 Male	1503	1477	TRUE	0.001367054	7.4319349	0.0285788	0.0622333	0.012702	0.0133812
2427	2011 Female	1496	1373	TRUE	0.001367054	7.2209473	0.074173	0.0669876	0.0170898	0.037426
2428	2015 Male	1379	1488	TRUE	0.001367054	6.0741405	0.0072263	0.0507926	0	0.0171319
2429	1995 Male	1717	1411	FALSE	0	5.3916016	0.013916	0.0282745	0	0
2430	1989 Female	769	1745	FALSE	0	6.0199585	0.0176077	0.0706047	4.768E-05	0.055603
2431	2015 Male	1533	1528	TRUE	0.001367054	8.7276013	0.1006572	0.1744111	0.081022	0.0090883
2432	1993 Female	1714	1403	FALSE	0	6.671814	0.0119147	0.0832112	0.0110779	0.0011597
2433	1994 Male	1555	1604	FALSE	0	6.6729494	0.0069538	0.0678735	0.0185785	0.0074227
2434	2015 Female	1533	1525	TRUE	0.001367054	8.8168196	0.019032	0.1295146	0.0252831	0.0090883
2435	2015 Male	1533	1527	TRUE	0.001367054	8.8325319	0.0841965	0.1575268	0.0196607	0.0090883
2436	2009 Female	1740	1637	TRUE	0.001367054	6.7518359	0.0109335	0.0707748	0.035728	0.0049152
2437	1994 Male	1717	1577	FALSE	0	6.1365967	0.0129948	0.0754312	0.041626	0
2438	1994 Male	1555	1601	FALSE	0	6.8670411	0.0018386	0.1262287	0.1458397	0.0074227
2439	2012 Male	1537	1159	TRUE	0.001367054	7.7245178	0.0896589	0.1217119	0.069519	0.0734531
2440	2013 Female	1379	1764	TRUE	0.001367054	7.5787788	0.0167023	0.0850366	0.0493141	0.0171319
2441	2007 Male	1547	1738	TRUE	0.001367054	7.4249919	0.0095743	0.0707595	0.0204043	0.0355812
2442	1991 Female	1555	1213	FALSE	0	5.8018986	0.2538723	0.0206407	0.0006437	0.0074227
2443	1994 Female	1555	926	FALSE	0	6.1379457	0.0025572	0.062948	0.041626	0.0074227
2444	1994 Female	1717	1261	FALSE	0	6.5911865	0.00741	0.0830122	0.0555	0
2445	1993 Male	1555	1685	FALSE	0	5.860364	0.1320343	0.0227829	0.0061106	0.0074227
2446	2001 Female	1058	1532	TRUE	0.001367054	6.5307212	0.0094668	0.0539555	0.0008886	0.0142442
2447	1993 Female	1714	1763	FALSE	0	6.1855469	0.1354218	0.057324	0.027832	0.0011597
2448	1994 Male	1717	1416	FALSE	0	6.3696289	0.0778046	0.0698269	0.0345459	0
2449	2002 Female	1494	1522	TRUE	0.001367054	7.2438992	0.0137823	0.0747483	0.0008886	0.0082214
2450	2012 Female	1507	1559	TRUE	0.001367054	6.9581006	0.0206735	0.0388189	0.0073708	0.0065747
2451	1996 Male	1514	1130	FALSE	0	7.2540314	0.0091909	0.1196062	0.0662231	0.0036822
2452	1996 Female	1514	1402	FALSE	0	7.2347138	0.0165645	0.0818452	0.003417	0.0036822
2453	1996 Female	1514	1428	FALSE	0	7.5423005	0.0281335	0.0861878	0.0226765	0.0036822
2454	2016 Female	1533	1719	TRUE	0.001367054	8.4162045	0.0109814	0.1133765	0.0024699	0.0090883
2455	2013 Female	1538	1668	TRUE	0.001367054	7.7437592	0.0517345	0.1063089	0.0750617	0.0027952
2456	2017 Female	1737	1681	TRUE	0.001367054	8.1637996	0.0214681	0.0855767	0.0311776	0.010716
2457	1996 Male	1514	1601	FALSE	0	7.571872	0.0279679	0.1570235	0.1458397	0.0036822
2458	2019 Male	1724	1713	TRUE	0.001367054	8.7593584	0.0283307	0.1275279	0.0503962	0.0142475
2459	2009 Male	1740	999	TRUE	0.001367054	7.3616891	0.0171011	0.0745332	0.0064511	0.0049152
2460	1995 Male	1715	1602	FALSE	0	7.1469421	0.0573918	0.0966527	0.0085063	0.0005798

2461	2017	Female	1533	1709	TRUE	0.001367054	8.6618213	0.0180702	0.1987124	0.0935644	0.0090883
2462	2007	Female	1548	1308	TRUE	0.001367054	7.6579154	0.0071245	0.0748077	0.0064511	0.0267128
2463	1995	Male	1717	1213	FALSE	0	5.8005496	0.0039075	0.0197606	0.0006437	0
2464	2003	Female	1494	1532	TRUE	0.001367054	7.2438992	0.0137823	0.0747483	0.0008886	0.0082214
2465	2016	Male	1737	1722	TRUE	0.001367054	8.3094265	0.016215	0.0865973	0.035853	0.010716
2466	1994	Female	1331	1716	FALSE	0	6.3734193	0.011168	0.0315158	0.0023193	0.0122211
2467	2012	Male	1538	1536	TRUE	0.001367054	7.6197968	0.1519204	0.0988084	0.0863297	0.0027952
2468	2009	Male	1467	1738	TRUE	0.001367054	7.4676623	0.0142296	0.0795578	0.0204043	0.0039563
2469	1993	Female	1286	1563	FALSE	0	4.1803507	0	0.0086242	0	0.001301
2470	2019	Female	1729	1727	TRUE	0.001367054	8.2038913	0.0700516	0.063501	0.0036419	0.0103261
2471	1996	Female	1715	1607	FALSE	0	7.3491211	0.0312443	0.1053679	0.0308189	0.0005798
2472	1996	Male	1715	1602	FALSE	0	7.1469421	0.0573918	0.0966527	0.0085063	0.0005798
2473	1996	Male	1715	1570	FALSE	0	6.5146484	0.1129837	0.2105348	0.2929688	0.0005798
2474	2009	Male	1335	1757	TRUE	0.001367054	7.800766	0.158848	0.1444959	0.0399272	0.038833
2475	2012	Male	1561	1194	TRUE	0.001367054	6.9735065	0.0079083	0.0541182	0.0102167	0.0138588
2476	2011	Female	1740	1208	TRUE	0.001367054	6.6292163	0.0073501	0.0452247	0.0083618	0.0049152
2477	2021	Male	1205	1560	TRUE	0.001367054	7.1721683	0.0183837	0.064513	0.0062427	0.0248988
2478	2012	Female	1507	1753	TRUE	0.001367054	7.7533289	0.0063735	0.0946902	0.074173	0.0065747
2479	2016	Female	1533	1710	TRUE	0.001367054	8.6618213	0.0180702	0.1987124	0.0935644	0.0090883
2480	2020	Female	1736	1546	TRUE	0.001367054	6.902171	0.0100557	0.0524358	0.0081406	0.0174069
2481	2010	Female	1467	1751	TRUE	0.001367054	7.6796008	0.0457117	0.0818717	0.0061956	0.0039563
2482	2014	Male	1538	1536	TRUE	0.001367054	7.6197968	0.1519204	0.0988084	0.0863297	0.0027952
2483	2014	Male	1538	1689	TRUE	0.001367054	7.455719	0.081554	0.070907	0.04261	0.0027952
2484	1996	Male	1715	1592	FALSE	0	6.5849609	0.0952682	0.0806982	0.0234375	0.0005798
2485	1995	Female	1515	1421	FALSE	0	7.0268555	0.0323601	0.075632	0.0308189	0.0023193
2486	2014	Female	1740	1687	TRUE	0.001367054	7.6049302	0.0126302	0.0728867	0.0161406	0.0049152
2487	2016	Female	1735	1732	TRUE	0.001367054	8.4454863	0.0133531	0.0860904	0.0115946	0.0102488
2488	1996	Male	1515	1255	FALSE	0	6.4243164	0.04953	0.0819483	0.0541992	0.0023193
2489	2019	Female	1729	1725	TRUE	0.001367054	8.5819561	0.0161913	0.0842142	0.0230972	0.0103261
2490	1996	Female	1515	1095	FALSE	0	5.7392578	0.0771484	0.0309096	0.0390625	0.0023193
2491	2012	Male	1467	1746	TRUE	0.001367054	7.4676623	0.0142296	0.0795578	0.0204043	0.0039563
2492	2017	Female	1205	1512	TRUE	0.001367054	7.8925129	0.0186448	0.0878664	0.0164408	0.0248988
2493	2011	Female	1561	1159	TRUE	0.001367054	6.9405518	0.0108132	0.0754017	0.069519	0.0138588
2494	1996	Male	1058	1517	FALSE	0	6.6684947	0.0006995	0.0712154	0.0009766	0.0142442
2495	2021	Female	1736	1560	TRUE	0.001367054	7.0967244	0.0137065	0.0568479	0.0062427	0.0174069
2496	1997	Female	1058	1522	FALSE	0	6.5307212	0.0094668	0.0539555	0.0008886	0.0142442
2497	2012	Female	1537	1298	TRUE	0.001367054	8.0140686	0.0800487	0.1243299	0.0715218	0.0734531
2498	2015	Male	1362	1519	TRUE	0.001367054	7.5732852	0.0065844	0.1082593	0.0399272	0.0277774
2499	2009	Male	1503	1524	TRUE	0.001367054	6.93618	0.0077916	0.0594224	0.0008886	0.0133812
2500	2015	Female	1362	1720	TRUE	0.001367054	7.9071275	0.0121288	0.0935276	0.0258458	0.0277774
2501	2013	Male	1454	1733	TRUE	0.001367054	8.1304301	0.095382	0.1097217	0.0124346	0.0158577
2502	2015	Female	1205	1751	TRUE	0.001367054	7.459004	0.0129677	0.0808866	0.0061956	0.0248988
2503	2018	Female	1362	1728	TRUE	0.001367054	7.8305786	0.0168023	0.0978149	0.0466036	0.0277774
2504	2003	Male	1407	1517	TRUE	0.001367054	6.7531738	0.0266857	0.0890828	0.0009766	0.0150452
2505	2007	Female	1297	1539	TRUE	0.001367054	7.2759094	0.0803264	0.0693672	0.0106437	0.0266709
2506	2009	Female	1297	1741	TRUE	0.001367054	7.3870544	0.1427837	0.0750418	0.0115356	0.0266709
2507	2012	Female	1297	1742	TRUE	0.001367054	7.7522278	0.0644332	0.102234	0.0525908	0.0266709
2508	2012	Female	1297	1741	TRUE	0.001367054	7.3870544	0.1427837	0.0750418	0.0115356	0.0266709
2509	2013	Female	1297	1743	TRUE	0.001367054	7.8964539	0.0887087	0.1057471	0.0651109	0.0266709
2510	2014	Male	1297	1742	TRUE	0.001367054	7.7522278	0.0644332	0.102234	0.0525908	0.0266709
2511	2013	Female	1297	1741	TRUE	0.001367054	7.3870544	0.1427837	0.0750418	0.0115356	0.0266709
2512	2014	Male	1744	1672	TRUE	0.001367054	8.0541077	0.0720571	0.1373701	0.0063049	0.1348806
2513	2014	Male	1744	1453	TRUE	0.001367054	8.2082977	0.1117451	0.1931985	0.10779	0.1348806
2514	2016	Female	1744	1672	TRUE	0.001367054	8.0541077	0.0720571	0.1373701	0.0063049	0.1348806
2515	2016	Female	1744	1742	TRUE	0.001367054	8.2099457	0.0821361	0.154847	0.0525908	0.1348806
2516	2013	Female	1197	1758	TRUE	0.001367054	7.5136295	0.0134979	0.0667415	0.0292342	0.0181582

2517	2012 Female	1561	1308	TRUE	0.001367054	7.2805583	0.0134175	0.0633397	0.0064511	0.0138588
2518	1999 Male	1187	1522	FALSE	0	6.3745139	0.0078666	0.051822	0.0008886	0.0071462
2519	1999 Male	1187	1734	FALSE	0	6.5912858	0.0062698	0.051161	0.0009323	0.0071462
2520	2014 Female	1468	1512	TRUE	0.001367054	6.9815484	0.0183744	0.0585729	0.0164408	0.0063477
2521	2019 Female	1736	1650	TRUE	0.001367054	7.081823	0.0186074	0.0532596	0.0120383	0.0174069
2522	2018 Female	1205	1512	TRUE	0.001367054	7.8925129	0.0186448	0.0878664	0.0164408	0.0248988
2523	2019 Female	1736	1560	TRUE	0.001367054	7.0967244	0.0137065	0.0568479	0.0062427	0.0174069
2524	2009 Female	1648	1553	TRUE	0.001367054	6.9864204	0.0141157	0.0856553	0.0060093	0.0522922
2525	2015 Female	1523	1526	TRUE	0.001367054	8.1921692	0.0325222	0.0995378	0.0124522	0.0428074
2526	2009 Female	1761	1556	TRUE	0.001367054	6.5661281	0.0106408	0.043394	0.0150617	0.0020294
2527	2009 Female	1205	1546	TRUE	0.001367054	6.9776149	0.0142408	0.0601009	0.0081406	0.0248988
2528	2020 Male	1430	1728	TRUE	0.001367054	7.2768912	0.0324851	0.0930198	0.0466036	0.0111939
2529	2010 Male	1430	1560	TRUE	0.001367054	6.5653932	0.0306144	0.0478904	0.0062427	0.0111939
2530	2009 Female	1379	1550	TRUE	0.001367054	6.8584118	0.0223255	0.0512109	0.016222	0.0171319
2531	2009 Female	1379	1759	TRUE	0.001367054	7.3413317	0.0227131	0.0858167	0.0492846	0.0171319
2532	2010 Male	1205	1559	TRUE	0.001367054	6.9137099	0.0308521	0.0543349	0.0073708	0.0248988
2533	2010 Female	1561	1551	TRUE	0.001367054	6.9807923	0.0166543	0.0540512	0.0161836	0.0138588
2534	2013 Female	1430	1731	TRUE	0.001367054	7.1460766	0.0331348	0.0685745	0.0179523	0.0111939
2535	2021 Female	1362	1728	TRUE	0.001367054	7.8305786	0.0168023	0.0978149	0.0466036	0.0277774
2536	2018 Male	1737	1731	TRUE	0.001367054	8.3720503	0.0209359	0.0857047	0.0179523	0.010716
2537	2006 Male	1291	1738	TRUE	0.001367054	7.0912657	0.0179765	0.061282	0.0204043	0.0142442
2538	2000 Female	1058	1522	TRUE	0.001367054	6.5307212	0.0094668	0.0539555	0.0008886	0.0142442
2539	2006 Female	1436	1517	TRUE	0.001367054	6.8757019	0.044857	0.115881	0.0009766	0.0120339
2540	2016 Male	1454	1733	TRUE	0.001367054	8.1304301	0.095382	0.1097217	0.0124346	0.0158577
2541	2011 Female	1205	1512	TRUE	0.001367054	7.8925129	0.0186448	0.0878664	0.0164408	0.0248988
2542	2014 Female	1430	1531	TRUE	0.001367054	7.0952488	0.0175053	0.059876	0.0107064	0.0111939
2543	2015 Male	1468	1731	TRUE	0.001367054	6.8418871	0.0495631	0.0559037	0.0179523	0.0063477
2544	2016 Male	1533	1525	TRUE	0.001367054	8.8168196	0.019032	0.1295146	0.0252831	0.0090883
2545	2006 Female	1436	1517	TRUE	0.001367054	6.8757019	0.044857	0.115881	0.0009766	0.0120339
2546	2021 Male	1542	1726	TRUE	0.001367054	7.1943207	0.0188064	0.0943847	0.071789	0.0136385
2547	2008 Male	1503	1524	TRUE	0.001367054	6.93618	0.0077916	0.0594224	0.0008886	0.0133812
2548	2015 Female	1762	1722	TRUE	0.001367054	7.4175079	0.030358	0.0646774	0.035853	0.0112391
2549	1999 Female	1058	1524	FALSE	0	6.5307212	0.0094668	0.0539555	0.0008886	0.0142442
2550	2011 Male	1323	1519	TRUE	0.001367054	7.559673	0.0236993	0.1029671	0.0399272	0.0037206
2551	2017 Male	1430	1528	TRUE	0.001367054	7.185574	0.020038	0.1241914	0.081022	0.0111939
2552	2008 Male	1436	1517	TRUE	0.001367054	6.8757019	0.044857	0.115881	0.0009766	0.0120339
2553	2009 Male	1323	1519	TRUE	0.001367054	7.559673	0.0236993	0.1029671	0.0399272	0.0037206
2554	2011 Male	1755	1748	TRUE	0.001367054	7.7517472	0.015394	0.0755904	0.0204043	0.0292342
2555	2016 Female	1670	1536	TRUE	0.001367054	8.412384	0.2039669	0.1857396	0.0863297	0.0910081
2556	2013 Male	1747	1495	TRUE	0.001367054	8.4876066	0.0196557	0.1319082	0.0872439	0.0292342
2557	2011 Male	1538	1444	TRUE	0.001367054	7.2074585	0.0354408	0.055089	0.0013733	0.0027952
2558	2011 Female	1538	1539	TRUE	0.001367054	7.1350098	0.1643037	0.0477484	0.0106437	0.0027952
2559	2019 Male	1754	1765	TRUE	0.001367054	8.3066727	0.0335621	0.0828983	0.0208251	0.0185659
2560	2012 Male	1197	1543	TRUE	0.001367054	6.8449725	0.0103981	0.0505125	0.0134497	0.0181582
2561	2021 Female	1750	1531	TRUE	0.001367054	8.1946996	0.0246808	0.0791204	0.0107064	0.0264095
2562	2013 Female	1197	1543	TRUE	0.001367054	6.8449725	0.0103981	0.0505125	0.0134497	0.0181582
2563	2008 Male	1323	1757	TRUE	0.001367054	7.559673	0.0236993	0.1029671	0.0399272	0.0037206
2564	2012 Female	1507	1752	TRUE	0.001367054	7.8417078	0.0085775	0.1070533	0.0868206	0.0065747
2565	2019 Female	1754	1308	TRUE	0.001367054	7.7895169	0.0182865	0.0792018	0.0064511	0.0185659
2566	2013 Female	1541	1748	TRUE	0.001367054	7.7517472	0.015394	0.0755904	0.0204043	0.0292342
2567	2019 Male	1507	1560	TRUE	0.001367054	7.216559	0.0183983	0.048997	0.0062427	0.0065747
2568	2007 Male	1756	1549	TRUE	0.001367054	6.8073569	0.0182463	0.0438983	0.009571	0.009695
2569	2006 Female	1760	1643	TRUE	0.001367054	6.1521324	0.0038238	0.031885	0.0028529	0.0066071
2570	2008 Female	1542	1759	TRUE	0.001367054	7.0106604	0.0272949	0.0824538	0.0492846	0.0136385
2571	2001 Male	1304	1513	TRUE	0.001367054	6.4416504	0.0006123	0.0675442	0.0009766	0.0096436
2572	2008 Male	1548	1471	TRUE	0.001367054	7.7858909	0.0134452	0.0704329	0.014887	0.0267128

2573	2014 Female	1708	1559	TRUE	0.001367054	7.3253693	0.0185444	0.0582233	0.0073708	0.0120457
2574	2019 Male	1702	1731	TRUE	0.001367054	7.8520981	0.0284963	0.0641676	0.0179523	0.0079816
2575	2017 Female	1538	1539	TRUE	0.001367054	7.1350098	0.1643037	0.0477484	0.0106437	0.0027952
2576	2016 Female	1508	1721	TRUE	0.001367054	4.510124	0	0.0459987	0	0.0120457
2577	2010 Female	1561	1553	TRUE	0.001367054	6.6781921	0.0109219	0.0454056	0.0060093	0.0138588
2578	2019 Female	1754	1485	TRUE	0.001367054	8.0415906	0.0386204	0.0744927	0.0104691	0.0185659
2579	2011 Female	1379	1550	TRUE	0.001367054	6.8584118	0.0223255	0.0512109	0.016222	0.0171319
2580	2011 Male	1561	1650	TRUE	0.001367054	6.9008434	0.0099042	0.0381676	0.0120383	0.0138588
2581	2011 Male	1561	1706	TRUE	0.001367054	7.65153	0.010136	0.076089	0.0474121	0.0138588
2582	2012 Female	1561	1554	TRUE	0.001367054	7.3190223	0.0220325	0.068311	0.0336794	0.0138588
2583	2017 Male	1533	1683	TRUE	0.001367054	8.3986684	0.016583	0.1119583	0.0105213	0.0090883
2584	2012 Male	1507	1546	TRUE	0.001367054	7.0220056	0.0119548	0.0445849	0.0081406	0.0065747
2585	2014 Male	1787	1324	TRUE	0.001367054	7.5268265	0.0305566	0.0723245	0.0005069	0.0042112
2586	2015 Male	1787	1683	TRUE	0.001367054	7.9699948	0.0283195	0.0697593	0.0105213	0.0042112
2587	2013 Female	1379	1817	TRUE	0.001367054	7.3844182	0.0152514	0.0690422	0.0337498	0.0171319
2588	2013 Male	1507	1814	TRUE	0.001367054	6.840365	0.0075316	0.0354633	0.0031357	0.0065747
2589	2022 Male	1205	1769	TRUE	0.001367054	7.7170647	0.0348333	0.08309	0.0127126	0.0248988
2590	2016 Female	1798	1553	TRUE	0.001367054	6.7548711	0.0107709	0.046324	0.0060093	0.0034083
2591	2021 Male	1791	1488	TRUE	0.001367054	7.2831421	0.052616	0.14004	0	0.1061264
2592	2007 Male	1609	1785	TRUE	0.001367054	4.4727631	0	0.0649744	0	0.0540786
2593	2022 Female	1737	1767	TRUE	0.001367054	9.0814638	0.0251396	0.1713741	0.1006572	0.010716
2594	2021 Male	1736	1776	TRUE	0.001367054	7.6984524	0.0183106	0.0742284	0.0149614	0.0174069
2595	2019 Female	1791	1813	TRUE	0.001367054	8.4115166	0.0055034	0.1343477	0.0051604	0.1061264
2596	1991 Male	1801	1397	FALSE	0	5.9453125	0.0755005	0.0890828	0.0639648	0.0263672
2597	2000 Male	1805	1059	TRUE	0.001367054	5.3537434	0.0067539	0.0342136	0.0078125	0.0378542
2598	2017 Female	1430	1786	TRUE	0.001367054	7.6294811	0.0235604	0.1174382	0.0432047	0.0111939
2599	2021 Female	1736	1827	TRUE	0.001367054	7.5125238	0.0168914	0.0668836	0.0129098	0.0174069
2600	2021 Male	1736	1818	TRUE	0.001367054	7.4394859	0.0146438	0.0602261	0.0115148	0.0174069
2601	2005 Male	1717	1790	TRUE	0.001367054	6.4799805	0.1220551	0.0654863	0.0771484	0
2602	2010 Male	1774	1628	TRUE	0.001367054	8.2732669	0.0122662	0.1554841	0.0251954	0.0137823
2603	2020 Female	1736	1822	TRUE	0.001367054	7.8329389	0.0497175	0.0863508	0.0305687	0.0174069
2604	1998 Male	1407	1773	FALSE	0	7.4460786	0.0759171	0.0723932	0.0042548	0.0150452
2605	2021 Male	1542	1775	TRUE	0.001367054	7.3035338	0.0529908	0.0740413	0.013938	0.0136385
2606	2021 Female	1780	1718	TRUE	0.001367054	8.7365328	0.0620502	0.1433773	0.0158758	0.0135489
2607	2016 Female	1205	1768	TRUE	0.001367054	7.9165092	0.0183507	0.0800854	0.0115958	0.0248988
2608	2015 Male	1820	1707	TRUE	0.001367054	7.5040271	0.0118477	0.0654684	0.0169171	0.0074084
2609	1994 Male	1286	1772	FALSE	0	5.3561319	0.0118418	0.0125304	0	0.001301
2610	2020 Male	1820	1705	TRUE	0.001367054	7.9080722	0.0126443	0.083544	0.0105538	0.0074084
2611	2019 Female	1770	1806	TRUE	0.001367054	8.2409807	0.0162759	0.1036853	0.0102499	0.0182211
2612	2022 Female	1789	1650	TRUE	0.001367054	7.9785149	0.0230123	0.0621861	0.0120383	0.0115856
2613	2017 Male	1825	1560	TRUE	0.001367054	7.6735403	0.0118813	0.0725307	0.0062427	0.0452458
2614	2020 Female	1729	1777	TRUE	0.001367054	8.1299408	0.0138267	0.0813205	0.0038638	0.0103261
2615	1988 Female	1781	1573	FALSE	0	3.8504639	0	0.076285	0.0523071	0
2616	2021 Male	1541	1771	TRUE	0.001367054	8.3981805	0.0476508	0.0943466	0.0403073	0.0292342
2617	2002 Male	1655	1788	TRUE	0.001367054	8.1307373	0.0583365	0.1776982	0.0323601	0.0803757
2618	2002 Male	1655	1782	TRUE	0.001367054	7.7838135	0.0583954	0.1862808	0.0472412	0.0803757
2619	2003 Male	1609	1819	TRUE	0.001367054	7.7510681	0.0319164	0.1280615	0.0094719	0.0540786
2620	2021 Male	1736	1824	TRUE	0.001367054	7.5526944	0.0165151	0.0682788	0.015133	0.0174069
2621	2022 Male	1796	1827	TRUE	0.001367054	8.1146769	0.0299685	0.0756097	0.0129098	0.0140485
2622	2004 Female	1035	1779	TRUE	0.001367054	7.2238011	0.0032774	0.0778508	0.0091909	0.0106385
2623	2020 Male	1783	1710	TRUE	0.001367054	8.7683414	0.1743262	0.2135383	0.0935644	0.0166911
2624	2020 Female	1533	1793	TRUE	0.001367054	9.1298013	0.0625243	0.1401187	0.0325222	0.0090883
2625	2015 Female	1744	1797	TRUE	0.001367054	8.5531998	0.135551	0.1752693	0.0475638	0.1348806
2626	2021 Female	1736	1828	TRUE	0.001367054	7.3519492	0.0150991	0.067396	0.0165755	0.0174069
2627	2019 Female	1820	1705	TRUE	0.001367054	7.9080722	0.0126443	0.083544	0.0105538	0.0074084
2628	2020 Male	1737	1778	TRUE	0.001367054	7.8711306	0.0146294	0.074563	0.0272039	0.010716

2629	2018	Female	1362	1768	TRUE	0.001367054	7.8634215	0.0120345	0.0682577	0.0115958	0.0277774
2630	2009	Male	1379	1814	TRUE	0.001367054	6.6943188	0.0123695	0.0402094	0.0031357	0.0171319
2631	2022	Female	1205	1776	TRUE	0.001367054	7.7738963	0.0325948	0.0818935	0.0149614	0.0248988
2632	2014	Female	1787	1525	TRUE	0.001367054	8.3881459	0.0300054	0.0873156	0.0252831	0.0042112
2633	2014	Female	1787	1327	TRUE	0.001367054	8.2774801	0.0451085	0.1414297	0.0334404	0.0042112
2634	2014	Male	1787	1739	TRUE	0.001367054	8.1602073	0.0415018	0.0661956	0.0115689	0.0042112
2635	2014	Female	1787	1528	TRUE	0.001367054	8.2989276	0.0586234	0.1322121	0.081022	0.0042112
2636	2014	Male	1787	1327	TRUE	0.001367054	8.2774801	0.0451085	0.1414297	0.0334404	0.0042112
2637	2019	Female	1770	1458	TRUE	0.001367054	7.7820133	0.0142023	0.1269505	0.1352	0.0182211
2638	2014	Male	1787	1327	TRUE	0.001367054	8.2774801	0.0451085	0.1414297	0.0334404	0.0042112
2639	2014	Female	1787	1528	TRUE	0.001367054	8.2989276	0.0586234	0.1322121	0.081022	0.0042112
2640	2011	Female	1205	1802	TRUE	0.001367054	7.8355154	0.0148348	0.0739391	0.008765	0.0248988
2641	2021	Female	1736	1769	TRUE	0.001367054	7.6416208	0.0203906	0.0754249	0.0127126	0.0174069
2642	2018	Female	1798	1730	TRUE	0.001367054	7.0288134	0.0083001	0.0457277	0.0051007	0.0034083
2643	2022	Female	1800	1828	TRUE	0.001367054	7.8184318	0.0133609	0.0653892	0.0165755	0.0129098
2644	2018	Male	1820	1705	TRUE	0.001367054	7.9080722	0.0126443	0.083544	0.0105538	0.0074084
2645	2018	Male	1729	1777	TRUE	0.001367054	8.1299408	0.0138267	0.0813205	0.0038638	0.0103261
2646	2000	Male	1792	1663	TRUE	0.001367054	7.8008084	0.0171883	0.0772672	0.0113038	0.0008292
2647	2011	Female	1541	1809	TRUE	0.001367054	8.3401971	0.110691	0.0709569	0.008765	0.0292342
2648	2017	Male	1820	1705	TRUE	0.001367054	7.9080722	0.0126443	0.083544	0.0105538	0.0074084
2649	2017	Female	1820	1707	TRUE	0.001367054	7.5040271	0.0118477	0.0654684	0.0169171	0.0074084
2650	2013	Female	1140	1795	TRUE	0.001367054	7.0652075	0.0190291	0.0656448	0.0257114	0.010376
2651	2002	Female	1794	1749	TRUE	0.001367054	6.4039916	0.0071752	0.0375156	0.0173889	0.0004883
2652	2011	Male	1197	1807	TRUE	0.001367054	7.2911481	0.0092033	0.0515891	0.007105	0.0181582
2653	2021	Male	1723	1826	TRUE	0.001367054	8.4493349	0.0133069	0.0976865	0.0082034	0.0125601
2654	2012	Male	1811	1748	TRUE	0.001367054	7.5710873	0.1369928	0.066655	0.0204043	0.0103981
2655	2021	Male	1504	1808	TRUE	0.001367054	7.1437911	0.0059271	0.0484174	0.0175053	0.0016036
2656	2015	Male	1538	1810	TRUE	0.001367054	7.7206573	0.1062372	0.0945292	0.0803264	0.0027952
2657	2008	Female	1344	1819	TRUE	0.001367054	7.9193175	0.0130996	0.1086098	0.0094719	0.0158104
2658	2012	Male	1197	1807	TRUE	0.001367054	7.2911481	0.0092033	0.0515891	0.007105	0.0181582
2659	2017	Male	1205	1768	TRUE	0.001367054	7.9165092	0.0183507	0.0800854	0.0115958	0.0248988
2660	2019	Female	1507	1815	TRUE	0.001367054	7.7465845	0.0081825	0.070883	0.0354408	0.0065747
2661	2019	Female	1507	1799	TRUE	0.001367054	7.7144147	0.0262155	0.075934	0.0235567	0.0065747
2662	2017	Female	1774	1661	TRUE	0.001367054	8.2790527	0.0302717	0.0856224	0.010897	0.0137823
2663	2017	Female	1798	1730	TRUE	0.001367054	7.0288134	0.0083001	0.0457277	0.0051007	0.0034083
2664	2018	Male	1820	1707	TRUE	0.001367054	7.5040271	0.0118477	0.0654684	0.0169171	0.0074084
2665	2014	Male	1787	1324	TRUE	0.001367054	7.5268265	0.0305566	0.0723245	0.0005069	0.0042112
2666	2018	Female	1737	1810	TRUE	0.001367054	8.3556179	0.0105931	0.1146786	0.0803264	0.010716
2667	2019	Female	1205	1776	TRUE	0.001367054	7.7738963	0.0325948	0.0818935	0.0149614	0.0248988
2668	2014	Female	1197	1807	TRUE	0.001367054	7.2911481	0.0092033	0.0515891	0.007105	0.0181582
2669	2002	Male	1027	1795	TRUE	0.001367054	6.5245275	0.0142496	0.0508726	0.0257114	0
2670	2021	Male	1736	1815	TRUE	0.001367054	7.6267499	0.0089114	0.0787339	0.0354408	0.0174069
2671	2020	Male	1736	1799	TRUE	0.001367054	7.5945801	0.0487836	0.083785	0.0235567	0.0174069
2672	2012	Male	1774	1628	TRUE	0.001367054	8.2732669	0.0122662	0.1554841	0.0251954	0.0137823
2673		Female	1804	1812	FALSE	0	1	0	0	0	0
2674		Male	1816	1620	FALSE	0	4.3581543	0.0257721	0.0204664	0	0.0263062
2675	2011	Male	1803	1821	TRUE	0.001367054	6.8969765	0.0734259	0.088025	0.0426845	0.0326812
2676	2011	Male	1507	1814	TRUE	0.001367054	6.840365	0.0075316	0.0354633	0.0031357	0.0065747
2677	2002	Male	1652	1784	TRUE	0.001367054	7.1887054	0.0424536	0.1367901	0.0771484	0.053371
2678	2012	Female	1561	1814	TRUE	0.001367054	6.5395508	0.0140054	0.0282222	0.0031357	0.0138588
2679	2019	Male	1823	1766	TRUE	0.001367054	8.1811135	0.0246085	0.0807354	0.0142754	0.02511
